# Near-complete Middle Eastern genomes refine autozygosity and enhance disease-causing and population-specific variant discovery

**DOI:** 10.1038/s41588-025-02173-7

**Published:** 2025-05-05

**Authors:** Mohammadmersad Ghorbani, Shabir Moosa, Zenab Siddig, Radi Farhad, Haroon Naeem, William T. Harvey, Francesco Kumara Mastrorosa, Katherine M. Munson, Rozaimi Mohamad Razali, Elbay Aliyev, Ilhame Diboun, Rawan Abouelhassan, Melissa Tauro, Sondoss Hassan, Rebecca Mathew, Muna Al Hashmi, Lisa S. Mathew, Kun Wang, Abdul Rahman Salhab, Fazulur Rehaman Vempalli, Ahmed El Khouly, Qatar Genome Program Research Consortium, Said I. Ismail, Wadha Al-Muftah, Radja Badji, Hamdi Mbarek, Dima Darwish, Tasnim Fadl, Heba Yasin, Maryem Ennaifar, Rania Abdellatif, Fatima Alkuwari, Muhammad Alvi, Yasser Al-Sarraj, Chadi Saad, Asmaa Althani, Eleni Fethnou, Fatima Qafoud, Eiman Alkhayat, Nahla Afifi, Wei Liu, Stephan Lorenz, Sara Tomei, Fazulur Rehaman Vempalli, Najeeb Syed, Hakeem Almabrazi, Ramzi Temanni, Ahmed El Khouly, Tariq Abu Saqri, Mohammedhusen Khatib, Mehshad Hamza, Tariq Abu Zaid, Tushar Pathare, Shafeeq Poolat, Rashid Al-Ali, Hamdi Mbarek, Omar Albagha, Souhaila Al-Khodor, Mashael Alshafai, Ramin Badii, Lotfi Chouchane, Xavier Estivill, Khalid Fakhro, Jithesh V. Puthen, Karsten Suhre, Zohreh Tatari, Younes Mokrab, Iman Alazwani, Sara Tomei, Khalid A. Fakhro, Alia Satti, Ruba Benini, Arang Rhie, Evan E. Eichler, Younes Mokrab

**Affiliations:** 1 Sidra Medicine, Doha, Qatar; 2 Department of Genome Sciences, University of Washington School of Medicine, Seattle, WA USA; 3 Department of Biomedical Science, College of Health Sciences, Qatar University, Doha, Qatar; 4 Department of Genetic Medicine, Weill Cornell Medicine, Doha, Qatar; 5 College of Health and Life Sciences, Hamad Bin Khalifa University, Doha, Qatar; 6 National Human Genome Research Institute, Bethesda, MD USA; 7 Howard Hughes Medical Institute, University of Washington, Seattle, WA USA; 8 Qatar Genome Program, Qatar Foundation Research Development and Innovation, Qatar Foundation, Doha, Qatar; 9 Qatar Biobank for Medical Research, Qatar Foundation, Doha, Qatar; 10 Molecular Genetics Lab, Hamad Medical Corporation, Doha, Qatar; 11 Bioinformatics Core, Weill Cornell Medicine, Doha, Qatar; 12 Department of Biophysics and Physiology, Weill Cornell Medicine, New York City, NY USA

**Keywords:** Medical genetics, Genomics

## Abstract

Advances in long-read sequencing have enabled routine complete assembly of human genomes, but much remains to be done to represent broader populations and show impact on disease-gene discovery. Here, we report highly accurate, near-complete and phased genomes from six Middle Eastern (ME) family trios (*n* = 18) with neurodevelopmental conditions, representing ancestries from Sudan, Jordan, Syria, Qatar and Afghanistan. These genomes revealed 42.2 Mb of new sequence (13.8% impacting known genes), 75 new HLA/KIR alleles and strong signals of inbreeding, with ROH covering up to one-third of chromosomes 6 and 12 in one individual. Using assembly-based variant calling, we identified 23 de novo and recessive variants as strong candidates for causing previously unresolved symptoms in the probands. The ME genomes revealed unique variation relative to existing references, showing enhanced mappability and variant calling. These results underscore the value of de novo assembly for disease variant discovery and the need for sampled ME-specific references to better characterize population-relevant variation.

## Abstract (web summary)

Generation and analysis of high-quality, genome assemblies from Middle Eastern trios demonstrate the utility of ancestry-matched data and assembly-based variation analysis.

## Main

Advances in long-read sequencing and bioinformatics have enabled the generation of high-quality genome assemblies from various ancestries, ranging from Korea^1^ to Puerto Rico^2,3^. Recently, over a hundred near-complete and fully phased genomes have been produced by the Human Pangenome Research Consortium (HPRC)^4^ and the Chinese Pangenome Consortium (CPC)^5^, in addition to complete telomere-to-telomere (T2T) genomes for a haploid genome of primarily European ancestry (CHM13)^6^ and a diploid Han Chinese male (CHN1)^7^. This has allowed major gaps in GRCh38 to be filled, especially in complex and repetitive regions^6,8^, capturing previously inaccessible variation^9^ and uncovering genome mechanistic insights^9,10^. Nevertheless, complete genome assembly is yet to be leveraged in disease-gene discovery, especially in under-represented populations where there is a particular need for understanding haplotype diversity and structure, which is important in assessing variant pathogenicity.

The Middle Eastern (ME) populations lie at a historical intersection of human migration and civilization^11–14^. They are generally characterized by isolated structures and high consanguinity, leading to increased incidence of genetic conditions impacting normal development^15–17^, often due to founder mutations. Recent studies have identified peculiar ancestral groups such as Peninsular Arabs who were found to be the closest to the ancient population that migrated out of Africa^11,14^ and harbor a differential mutational spectrum of Mendelian disorders and cancer predisposition^18,19^. Currently, ME populations are heavily under-represented in genomic literature, lacking high-quality assemblies^20,21^ needed to shed light on how inbreeding shapes genome structure and disease architecture^20,21^.

**Extended Data Fig. 1:**
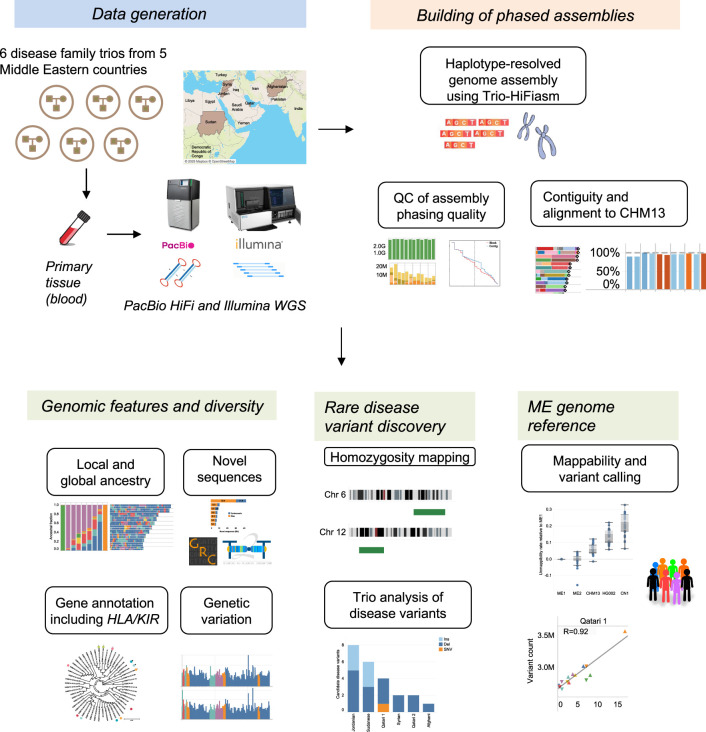
Study overview. The displayed map is from Mapbox and OpenStreetMap, used under the Open Database License (ODbL).

In this study, we use a de novo genome assembly approach to characterize six family trios from diverse ME ancestries (Sudan, Jordan, Syria, Qatar and Afghanistan), involving probands with various unresolved neurodevelopmental conditions whereby we (1) assemble near-complete genomes and map their high-resolution features, (2) apply assembly approach to identify disease-causing variants and (3) assess the value of ME-specific genetic reference in improving read mapping and variant calling (Extended Data Fig. 1). We assemble near-complete phased genomes which we interrogate for various structural features, comparing against the latest genome references. Using trio analyses, we identify previously undetected deleterious variants. Finally, we demonstrate the value of population-specific references, emphasizing the need for a carefully sampled pangenome for ME ancestries.

## Results

### Study samples and sequencing

**Fig. 1:**
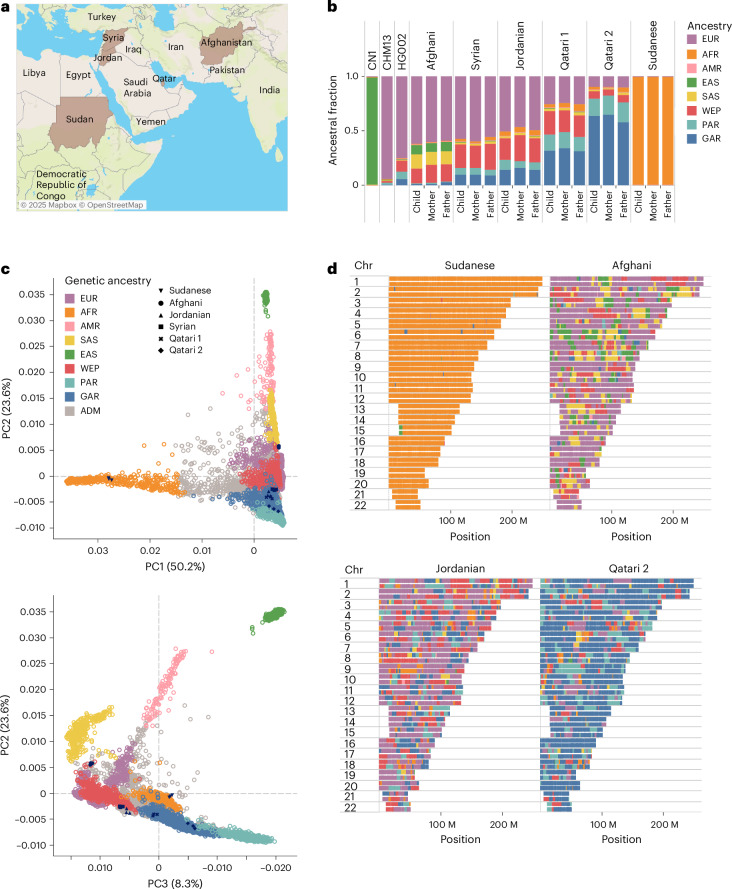
Genetic ancestry of study samples. **a**, Geographic location of the study cohort. **b**, Global ancestral composition of the individual participants alongside that of CN1, CHM13 and HG002. **c**, Principal component analysis showing the study samples and reference dataset from QGP and 1KG. **d**, Local ancestry analysis showing the genetic ancestral makeup of each chromosome for the Sudanese, Afghani, Jordanian and Qatari 2 child participants. The displayed map is from Mapbox and OpenStreetMap, used under the ODbL. ADM, admixed; AFR, Africans; AMR, American; EAS, East Asian; EUR, Europeans; SAS, South Asian; ODbL, Open Database License. Source data

**Extended Data Fig. 2:**
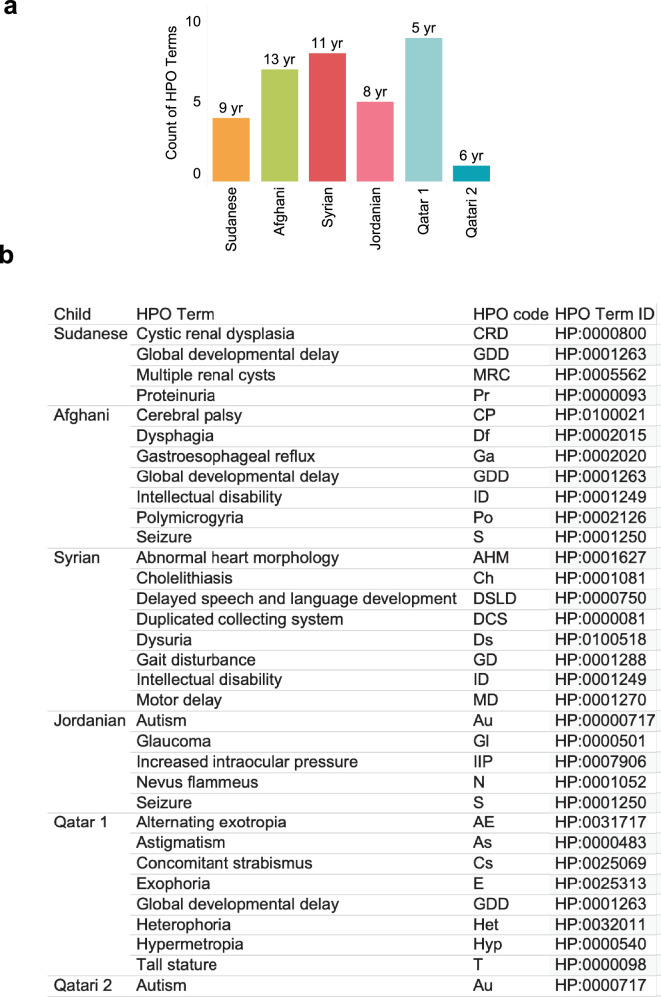
Clinical phenotypes of the children from the family trios in this study. **(a)** Age and count of Human Phenotype Ontology (HPO) terms per child. **(b)** Detailed list of HPO terms and HPO IDs. Source data

Our study samples consist of six parent–child trios (*n* = 18) from five different nationalities spanning the wide ME region: two trios from Qatar (Qatari 1 and Qatari 2) and single trios from Sudan, Jordan, Syria and Afghanistan (Fig. 1a). The children, male (XY) except for the Afghani female (XX), are aged 3–15 years and were ascertained with multiple symptoms of developmental disorders (Extended Data Fig. 2). Three of these families (Sudanese, Jordanian, Qatari 2) have undergone genetic testing previously based on microarrays and selected panels, returning negative results. Subsequent analysis using whole-genome sequencing from Illumina (Methods) did not identify plausible pathogenic variants except for Qatari 1, where we found a likely pathogenic variant (a de novo SNV in splice donor site) in the transcription factor nuclear factor IX (*NFIX*) reported to cause Malan syndrome^22^. This finding explained some of this patient’s symptoms, namely global developmental delay and tall stature.

Given the lack of diagnosis of these diverse ME families, we used the latest technologies of long-read sequencing and de novo assembly methods to gain insight into high-resolution genomic structure and disease-causing genetic variants. For each participant, we generated high-quality PacBio HiFi long-read and Illumina short-read data and subsequently processed them using established pipelines (Methods). For the long-read data, the average read length is 19 kb (range = 15.1–21.3 kb), while the average coverage is 38.8× for the children and 37.2× for the parents (Supplementary Fig. 1). For the short-read data, the average coverage is 31–38× (Supplementary Fig. 2).

### Genetic ancestry

To explore the detailed genetic ancestry of the ME trios, we conducted admixture inference of global and local ancestry based on a diverse population dataset as a reference panel based on 1000 Genomes (1KG) and Qatar Genome Program (QGP) phase 1 cohort (Methods). As expected, CHM13 is predominantly of European ancestry (Fig. 1b). The Sudanese trio has >99% African ancestry, while the Afghani, Syrian, Jordanian, Qatari 1 and Qatari 2 participants in this order showed a prominent trend of increasing General Arab (GAR) and Peninsular Arab (PAR) ancestries and decreasing European ancestry. The highest proportion of GAR was shown in Qatari 2 participants (63%), the highest PAR was in Qatari 1 and Qatari 2 (15% and 16%, respectively), while the highest West Eurasian and Persian (WEP) ancestry was found in the Syrian, Jordanian and Qatari 1 (20–22%; Fig. 1b). The highest East Asian and South Asian ancestries were found in the Afghani participants (8% and 12% respectively). Consistent with this, an overlay of the dominant global ancestries on principal components showed that the family trios co-clustered with samples of similar ancestries, namely GAR, PAR, WEP^14^ (for Syrian, Jordanian, Qatari 1 and Qatari 2), Africans (for Sudanese), while the Afghanis are found between Europeans, West Eurasians and Asians (Fig. 1c). Next, we conducted local ancestry inference to assign ancestry components to individual chromosomal segments (Fig. 1d and Supplementary Fig. 3). We could see relatively high ancestral homogeneity for the Sudanese participants as well as CHM13, while more admixture is seen for the remaining participants. The average ancestry segment length across the samples is 570 kb. Notably, the longest non-African stretches were found in Qatari 2 (PAR), Syrian (GAR) and Qatari 1 (WEP).

### Genome assembly and phasing

**Fig. 2:**
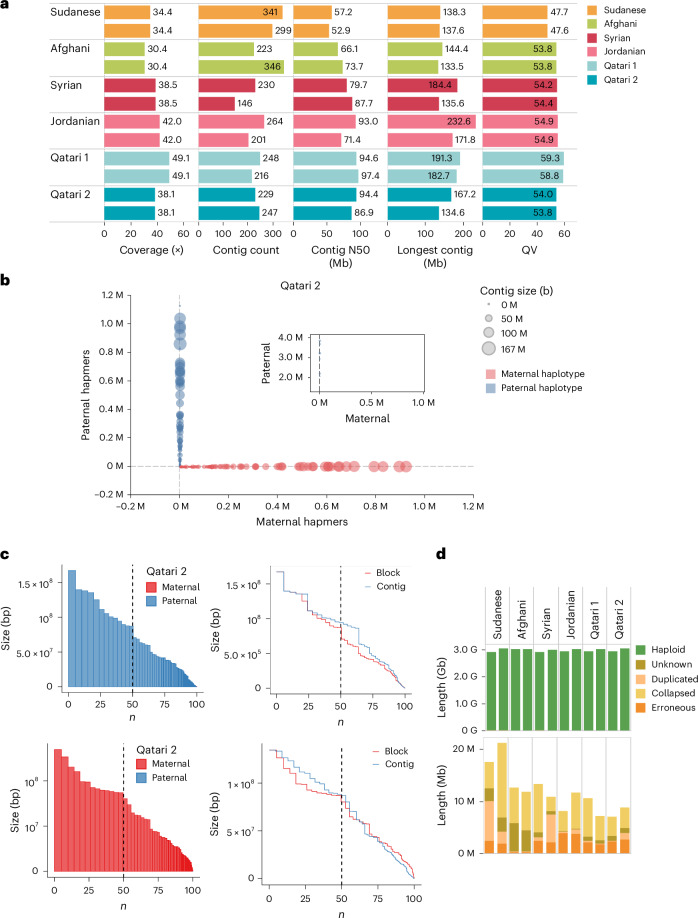
Assembly, phasing results and QC. **a**, QC metrics of each child assembly showing coverage, depth, total contigs, contig N50, maximum contig length and QV. **b**, Hapmer blob plot of Qatari 2 child sample showing a clear separation of maternal (red) and paternal (blue) haplotypes. Blob size is proportional to contig size, and each blob/contig is plotted according to the count of parental hapmers. **c**, Phase block NG plots (left) of haplotype-resolved assembly for paternal (top) and maternal (bottom) contigs sorted by size. The *x* axis represents the percentage of genome size and *y* axis represents the block size. Incorrectly phased haplotype blocks are virtually absent. Phase block NG and contig NG plots (right) showing the phase block sizes being similar to contig sizes due to low switch error. **d**, Reliability of the assemblies using read mapping evaluation with Flagger. Source data

We generated de novo phased assemblies for the six ME trios using trioHifiasm^23^, incorporating PacBio HiFi long reads and parental Illumina short reads, as per the best recommended workflow for phased genome assembly^23^. Standard quality check (QC) metrics were calculated to evaluate assembly contiguity and accuracy (Fig. 2a, Supplementary Fig. 4 and Supplementary Table 1). The obtained assemblies were of high quality as indicated by the various metrics, the ranges and averages of which are as follows: coverage (30.4–49.1×, 37.7×), contig N50 (52.9–98.2 Mb, 77.3 Mb), contig count (146–372, 263.4), longest contig (133.5–232.6 Mb, 169.2 Mb) and per-base accuracy quality value (QV; 44.0–59.3, 53.7). Of note, the children were better than the parents for most metrics, reflecting the higher mean coverage (38.8 versus 34.9), higher average HiFi read length (19.9 kb versus 18.8 kb) and the complementary use of parental data for assembling and phasing. The assembly with the best contig N50 was for Qatari 1 child (94.6 Mb for haplotype 1 and 97.4 Mb for haplotype 2) which also had the best QV (59.3 for haplotype 1 and 58.8 for haplotype 2), the best coverage (49.1×) and second highest HiFi read length average (21.0 kb). The longest contig was 232.6 Mb, belonging to the Jordanian child participant.

Using the parental short reads, we evaluated the phasing accuracy for the child assemblies using haplotype-specific markers (hapmers)^24^, whereby contigs are plotted as a function of hapmers assigned from maternal and paternal libraries (Fig. 2b and Supplementary Fig. 5). All assemblies show a clear segregation of the parental haplotypes indicating the high quality of phasing. Furthermore, N* plots indicate low levels of haplotype switch errors (Fig. 2c and Supplementary Fig. 6). In addition, inherited hapmer plots show balanced distributions of *k*-mers private to children, fathers, mothers and those shared between the parents (Supplementary Fig. 7). Furthermore, we performed an independent evaluation of the accuracy of the assemblies using the Flagger pipeline which assesses coverage and variants from the alignment of HiFi reads to the respective assemblies^4^. We found that >99% of the assemblies’ sequences consisted of reliable (haploid) blocks (Fig. 2d).

### Comparison with T2T assemblies and new sequences

**Fig. 3:**
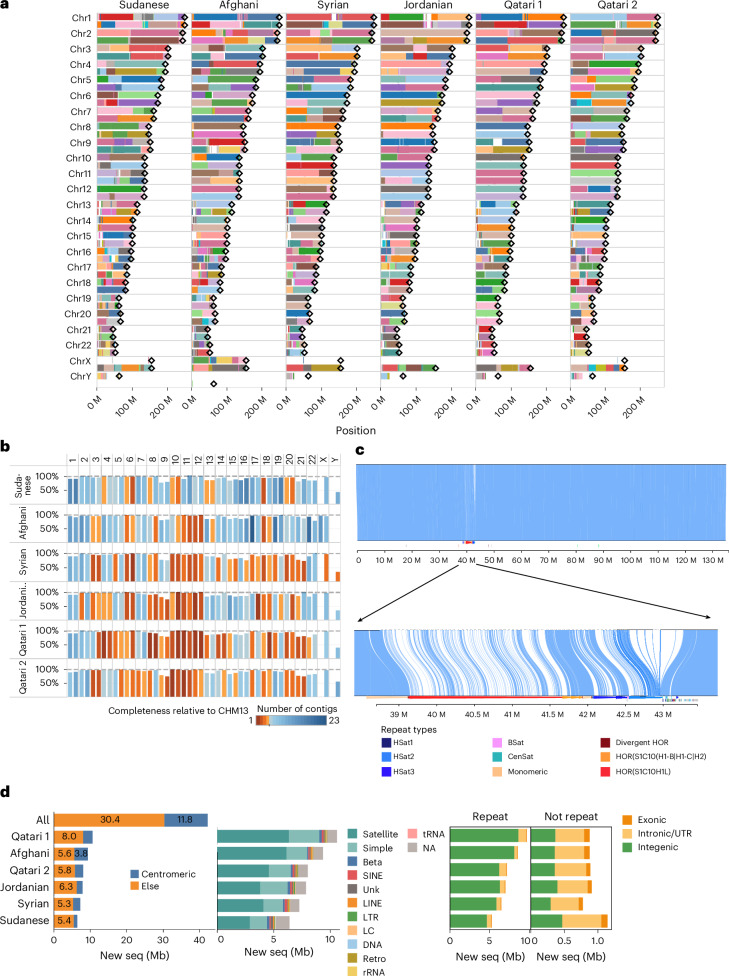
Contiguity, haplotype alignment to CHM13 and new sequences. **a**, Alignment of child assemblies to CHM13 for individual chromosomes. Individual contigs are delineated with distinct colors per chromosomal haplotype. Diamond symbols denote the end points of CHM13 haplotypes. **b**, Percentage of completeness (*y* axis) relative to CHM13 per chromosome (*x* axis), colored by the number of contigs in the alignment. **c**, Alignment of chromosome 10 of Qatari 2 (top) child assembly to CHM13 (bottom) showing a singular contig spanning the entire chromosome with notable centromeric variation. **d**, Length of new sequences identified across samples, highlighting location in centromeric regions (left), repetitive regions (middle) and intergenic, intronic/UTR and exonic regions either inside or outside repetitive regions (right). LC, low complexity; LTR, long terminal repeats; SINE, short interspersed nuclear element; Unk, unknown. Source data

Alignment of the child assemblies to CHM13 using Minimap2 (ref. ^25^) showed high mappability across various chromosomes, with an average of 91% of contigs with high alignment coverage mapping to a unique location (Fig. 3a and Supplementary Fig. 8). Using CHM13 as reference, the ratio of completeness per chromosome was 93% on average, with 43 chromosomes from all six participants having >99% completeness including 14 T2T chromosomes, which we verified confirming the presence of telomeric repeats (Fig. 3b). Qatari 2 shows the highest overall completeness of 92%. Notably, 14 chromosomes were spanned by a single contig covering the centromeric region, including a T2T single contig for chromosome 10 from a Qatari 2 child (Fig. 3c and Supplementary Fig. 9).

We checked the sequences of insertions identified from the alignments of the assemblies to CHM13 against a diverse set of highly curated calls of structural variations from HPRC^8^, HG002 (Ashkenazi Jews) and CN1 (Han Chinese)^7^, from which we excluded any regions identified with Flagger to be unreliable. This led to the identification of 42.2 Mb of new sequences across all the samples, ranging from 6.4 Mb for the Sudanese to 10.6 Mb for Qatari 1 (Fig. 3d). Of these sequences, 10–40% were in the centromeres, highlighting the relatively higher divergence and mutation rate in these regions^26^. By size, 86.2% of the new sequences are found in intergenic regions, while 12.6% are in intronic or untranslated regions (intronic/UTR) and 1.2% in exonic regions (Supplementary Fig. 10a). Also, 89.7% of the new sequences are found in repetitive regions, notably satellite (58.1%) and simple repeats (24.1%; Fig. 3d, middle). New sequences outside repetitive regions are enriched for exonic and intronic/UTR (Fig. 3d, right). We also noticed most new sequences (79.3%) occur outside segmental duplications (Supplementary Fig. 10b). For those inside segmental duplications, nearly a quarter overlap with intronic/UTR and 3.9% overlap with exonic regions (Supplementary Fig. 10b).

Consistent with the closer genetic distance to HG002 (ref. ^27^), the sequences from our assemblies, which were also absent from CHM13 and HPRC, have more shared sequences with HG002 than CN1 (Supplementary Fig. 10c). The new sequences are well spread across the chromosomes (Supplementary Fig. 10d,e).

### Gene coverage

**Fig. 4:**
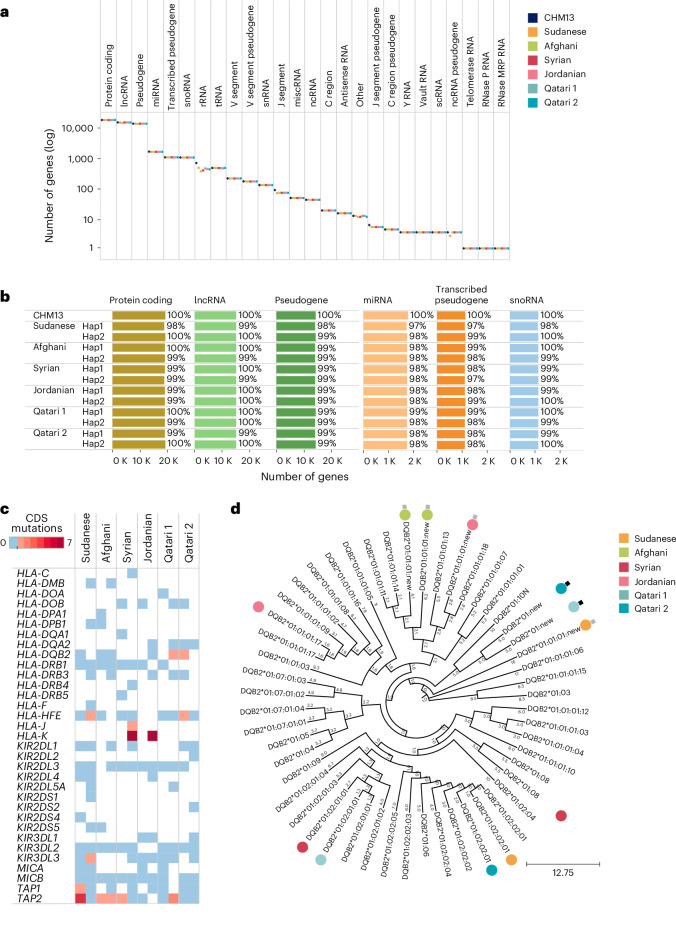
Gene coverage and HLA and KIR gene annotation. **a**, Gene counts across various gene categories. **b**, Coverage for the largest gene categories. **c**, New alleles in HLA and KIR genes in the child assembly haplotypes, highlighting the number of mutations in the CDS relative to a reference dataset of 220 pre-annotated reference haplotypes. **d**, Phylogenetic tree based on neighbor-joining method for the *HLA-DQB2* locus showing clustering pattern of the alleles in the child assemblies. New alleles are labeled, showing those with mutations in the CDS region (black squares) and those with mutations in other part of the sequence (gray squares). Next to each leaf-node connecting branch, the corresponding evolutionary distance is marked. Length key is shown in the bottom right. CDS, coding DNA sequence. Source data

We annotated the assemblies with genes of various types based on the publicly available CHM13 annotation. The distribution of genes identified across the chromosomes was found to be proportionate and similar to CHM13 (Supplementary Fig. 11a). Stratifying per-gene category, the number of genes across the assemblies is relatively uniform and similar to CHM13 for all established 27 gene categories, except for rRNA genes known to vary in number of copies between individuals^28^ (Fig. 4a). Specifically, the assemblies show 97–100% coverage for the six largest gene categories in the genome namely protein coding, LncRNA, pseudogenes, miRNA, transcribed pseudogenes and snoRNA (Fig. 4b). The number of genes observed for sex chromosome was consistent with the sample sex (Supplementary Fig. 11b,c). Per participant, Qatari 1 and 2 haplotypes demonstrate exceptionally high coverage, exceeding 99% for protein-coding genes.

### HLA gene family and KIR gene family annotations

**Extended Data Fig. 3:**
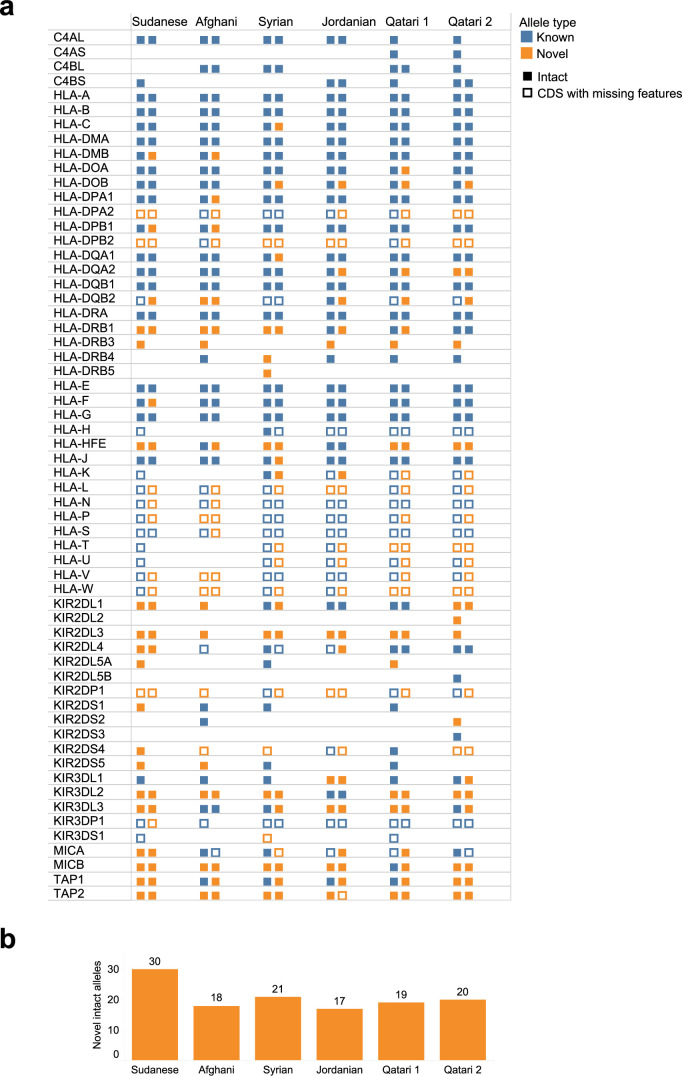
HLA and KIR gene annotations. **a**, Known and new alleles in HLA and KIR genes in the child assemblies’ haplotypes, highlighting intact sequences and those having CDS with missing features. **b**, Count of new alleles with intact CDS regions in each child assembly. Source data

We annotated HLA and KIR genes in the assemblies using a recent whole-genome-assembly-based method and compared the annotated genes against a set of 220 pre-annotated assembly haplotypes, including HLA assemblies, HPRC, CPC, CHM13, CN-T2T and GRCh38 (ref. ^29^). The ME child assemblies had alleles in 47 genes, all of which had one copy except *C4B* which had two copies (Supplementary Fig. 12). The genes harbored 233 alleles, including previously reported alleles in the *HLA-A* locus, which are consistently found to be largely common or well documented in populations of ME ancestry^30^ (Supplementary Fig. 13). Furthermore, amongst the 47 genes, we found 33 genes that contained 75 new alleles (that is absent from the Immuno Polymorphism Database (IPD)) with intact coding DNA sequence (CDS) region (Extended Data Fig. 3a). These alleles, of which there are 17–30 per assembly (Extended Data Fig. 1b), are found in regions of reliable assembly as determined using Flagger. They include 11 unique alleles, each having one to seven mutations in CDS regions (Fig. 4c and Supplementary Fig. 14a). In terms of impact on protein sequence, ten are new missense, five are known missense and ten are synonymous (Supplementary Fig. 14b,c). The DNA and protein sequence alignments highlighting these mutations are shown in Supplementary Fig. 15. Phylogenetic trees show the known alleles in the study participants are spread over multiple diverged lineages while some of the new alleles are found to be closely clustered, pointing to potential common founder lineages (Fig. 4d and Supplementary Fig. 16).

### Genetic variation

**Fig. 5:**
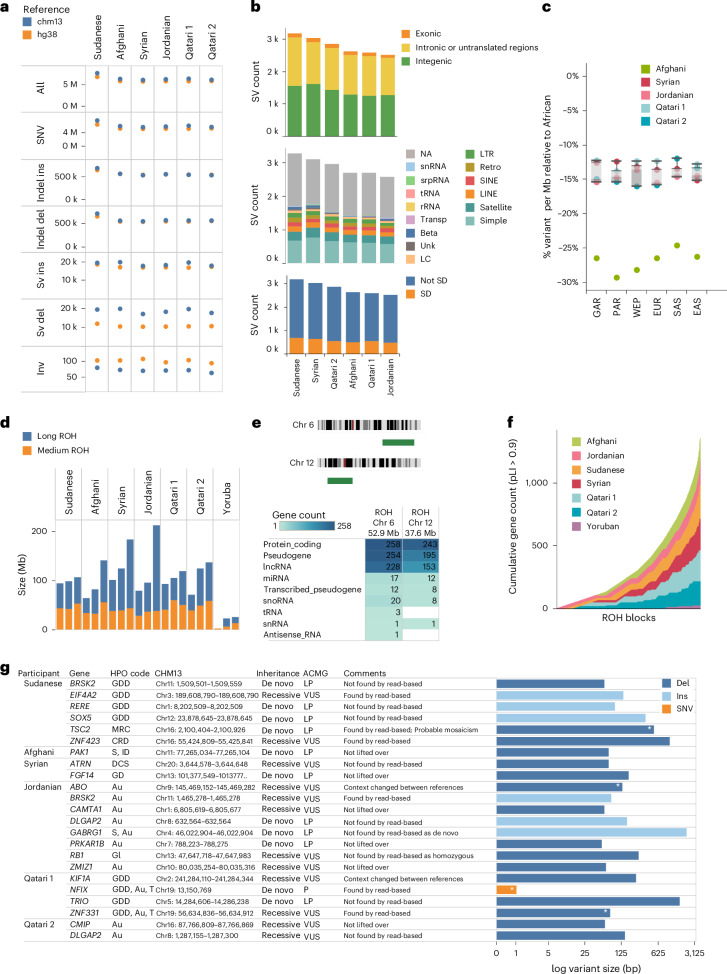
Genetic variation, ROH and candidate disease-causing variants. **a**, SV count against CHM13 and GRCh38 for each child assembly haplotype. **b**, Count of SV variants (deletions and insertions) in the family trios called against CHM13 and found to be absent from the HPRC dataset, highlighting their spread across intergenic, intronic/UTR and exonic regions (top), repetitive regions (middle) and segmental duplications (bottom). **c**, Box plot showing median counts of variants per MB relative to African segments in the same participants aggregated per family (*n* = 15), for various ancestries. **d**, Cumulative sizes of long and medium ROH of the ME assemblies and the Yoruba 1KG trio. **e**, Location and count of genes within the long ROH segments for chromosomes 6 and 12 of the Jordanian father. **f**, Cumulative number of genes (pLI > 0.9) over contigs per child assembly. **g**, Candidate disease-causing variants in the probands. Shown are the variants, impacted genes, ascertained phenotypes in the child participants and associated details. The comments column indicates whether the variant was identified with read-based calling. Exonic deletions are denoted by an asterisk on the bars. SD, segmental duplication; HPO, Human Phenotype Ontology; Au, autism; CRD, cystic renal dysplasia; DCS, duplicated collecting system; GD, gait disturbance; GI, glaucoma; GDD, global developmental delay; ID, intellectual disability; MRC, multiple renal cysts; S, seizure; T, tall stature; P, pathogenic; LP, likely pathogenic. Source data

To gain insight into the genetic variation in the ME assemblies, we compared them against the widely used genome references GRCh38 and CHM13, whereby we performed assembly-based alignment and called single-nucleotide variants (SNVs), short insertions and deletions (indels) and various types of structural variants (SVs). Assembly-based alignment relies on mapping assembled contigs against a given reference instead of individual reads in classical read-based alignment, and was shown previously to lead to better accuracy^31^. Consistent with this, using this approach, we observe more accurate calls than using read-based alignments (Supplementary Fig. 17). Overall, there were an average of 6.6% more variants against CHM13 than GRCh38, the largest being shown for Sudanese (10.3%; Fig. 5a). This is most prominent for SV deletions which are 61.5–87.2% higher. The exception to this trend is for inversions, whereby, on average, they are 43.2% less against CHM13 than GRCh38. Comparing the assemblies, the Sudanese has 18.6% more SNVs and indels on average than those of other participants, whereas SV counts are more uniform (Supplementary Fig. 18). We found that 1,177–1,568 deletions and 1,325–1,641 insertions across the six families were entirely absent from the HPRC dataset. Also, more than half of them (51–56%) fall in repetitive regions, notably simple repeats, while 19–22% overlap with segmental duplications (Fig. 5b).

Next, we overlaid the generic variants on local ancestry segments. The aggregated counts per ancestry were reflective of the global ancestry of individual participants, rather than showing specific enrichment (Supplementary Fig. 19). When we plotted the number of variants per 1 Mb of ancestral segment as a function of chromosomal coordinate, we saw that the counts across segments fluctuated between ancestries, as illustrated for the near-complete chromosome 10 (Supplementary Fig. 20a). When we aggregated that, we observe 12–30% lower variant counts relative to African segments (Fig. 5c), in line with population distances (Supplementary Fig. 20b).

### Runs of homozygosity (ROH)

**Extended Data Fig. 4:**
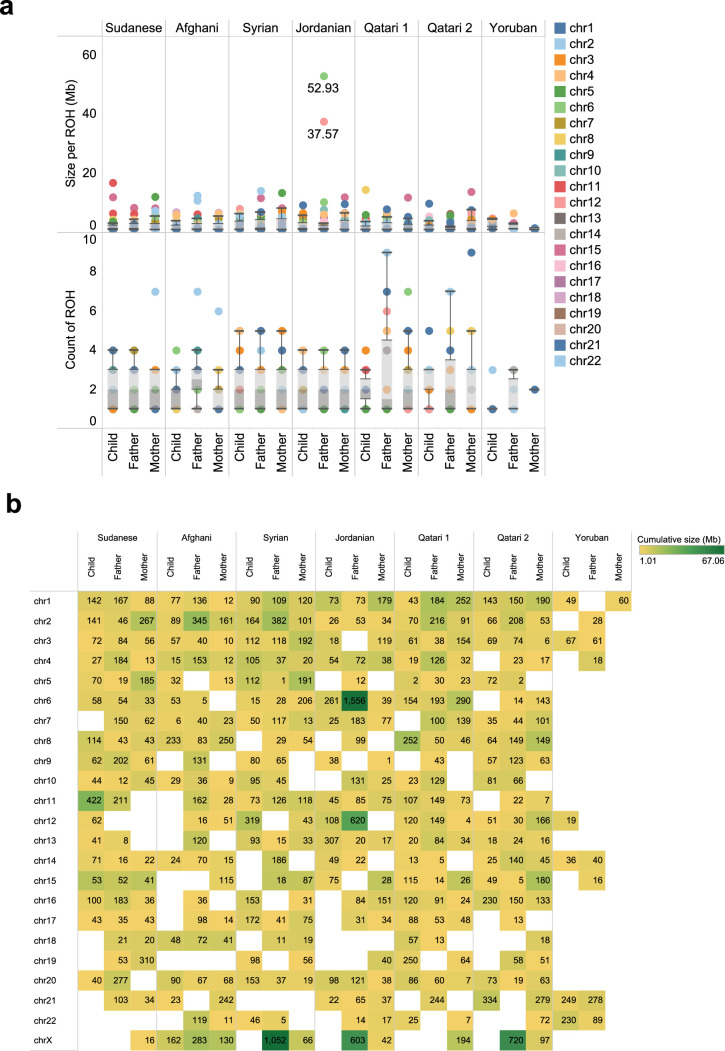
Runs of homozygosity (ROH) per chromosome. **a**, Size and count of ROH across chromosomes in all participants compared to YRI trios. **b**, Heatmap of ROH by chromosome and size, with color intensities reflecting the cumulative ROH size on each chromosome. Source data

**Extended Data Fig. 5:**
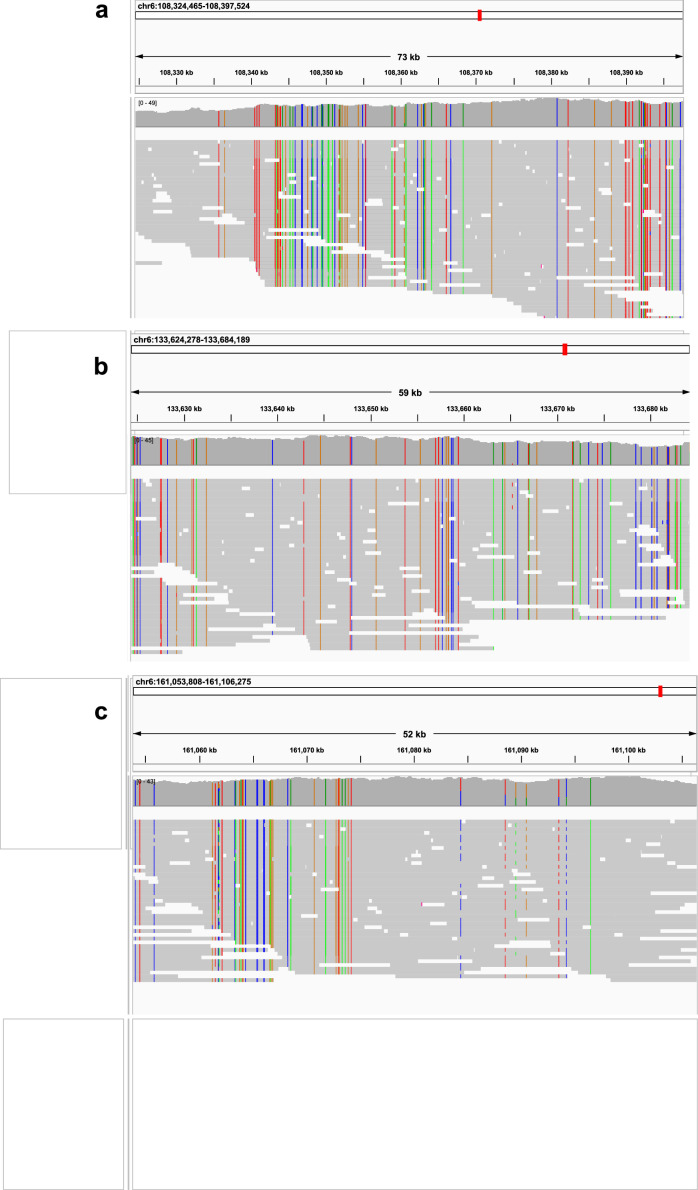
Long ROH in chr6 of the Jordanian father. **a**–**c**, IGV visualization of the HiFi read alignments against CHM13 showing the (**a**) start, (**b**) middle and (**c**) the end of the ROH region, revealing uniform homozygous region and introduction of heterozygous sites at the end of ROH.

In light of the background consanguinity known to characterize ME populations, we explored ROH in the assemblies, which we calculated from the assembly-based alignments to CHM13. We classified ROH to medium (1–3 Mb) and long (≥3 Mb), which respectively reflect distant and recent inbreeding^32,33^. ROH segments are found genome-wide up to nine per chromosome, with variable cumulative ROH per chromosome (Extended Data Fig. 4). Cumulatively, the ME participants had an average 43.8 Mb medium ROH and 69.1 Mb long ROH, which is fivefold greater than a reference Yoruban trio (Fig. 5d). The father in the Jordanian trio exhibits the largest cumulative ROH (212.3 Mb), of which 174.6 Mb is of the long type which is remarkable given that he has not presented any clinical phenotypes. There is a high correlation between ROH segment size and gene content (Pearson correlation coefficient, *R* = 0.8, *P* < 10^−4^; Supplementary Fig. 22). Various types of genes are found in these ROH segments, including protein coding, pseudogenes and LncRNA without any category being particularly enriched (Supplementary Fig. 21c). Notably, the Jordanian father, who is a healthy participant, harbors exceptionally long ROH segments on chromosomes 6 (52.9 Mb) and 12 (37.6 Mb), the longest amongst all participants, spanning 30% and 28% of the chromosomes and covering 794 and 620 genes, respectively (Fig. 5e). We confirmed these long ROHs by examining the alignments of HiFi reads against CHM13 which showed homozygous genotypes throughout the region and introduction of heterozygous sites at the ends (Extended Data Fig. 5). We note that of the 537 ROH segments identified in the study participants, 72% (386) encompass at least one protein-coding gene with high probability of loss-intolerance (pLI) score >0.9 (ref. ^34^; Fig. 5f).

### Genetic variants underlying disease phenotypes

We performed a trio-based analysis to search for disease-causing variants that explain the disease phenotypes in the child participants (Methods). We examined the SNVs, indels and SV variants obtained for families against CHM13. Given that only the children are affected, we considered recessive and de novo modes of inheritance. We prioritized variants that were rare in population databases (allele frequency, AF < 0.01), including gnomAD^35^, 1KG^36^, HPRC^8^ and QGP phase 1 cohort^14,37^, have high predicted functional scores and whereby the proband conditions are consistent with gene phenotypes reported in ClinGen^38^, Gene2Phenotype (G2P) panel on Developmental Disorders^39^ and DECIPHER^40^.

Overall, we shortlisted 23 candidate variants relevant to the various phenotypes across the six probands, comprising 16 deletions, 6 insertions and 1 SNV, each participant having between 1 and 8 variants (Fig. 5g and Supplementary Fig. 23). In terms of inheritance mode, 12 are recessive while 11 are de novo. Regarding their location, four are shown to impact exons, whereas the rest impact intronic regions and overlapping regulatory features, therefore providing strong candidates as disease-causing (Supplementary Table 2). Based on American College of Medical Genetics and Genomics (ACMG) guidelines, two of the variants are classified as pathogenic and one as likely pathogenic, found in the well-established genes of *NFIX* (associated with Malan Overgrowth Syndrome) and *TSC2* (associated with Tuberous Sclerosis Complex), in addition to *ABO* which is less known to be implicated in neurodevelopment. Furthermore, 20 variants were classified as variant of uncertain significance (VUS) in genes reported to have strong evidence of association in the literature (ACMG total score ≥ 0.45). These include 10 that we classified as high interest VUS (ACMG total score = 0.60–0.75; Supplementary Table 2), such as *SOX5* (delayed speech and language development) and *BRSK2* (neurodevelopmental disorders). Notably, in Qatari 1 proband who has global developmental delay, we identified a 234 bp Del in intron 5 of *KIF1A,* which encodes a motor protein essential for axonal transport of synaptic vesicle precursors. A de novo mutation in this gene has been previously reported to cause cerebellar atrophy and epilepsy in an Egyptian child from a consanguineous marriage^41^ and other variants in this gene have been classified by ClinGen and G2P to have a ‘definitive’ association with autosomal dominant syndromic intellectual disability. The largest shortlisted variant is a homozygous Del (2.2 kb) in the intronic region of *GABRG1* in the Jordanian proband who has seizures and autism. Variants in *GABRG1* are associated with seizures and global developmental delay^42^. This gene encodes the γ1 subunit of the GABA-A receptor and has been associated with epileptic encephalopathy under ‘limited’ class on G2P. Therefore, our finding supports a more assertive classification for this gene.

We observed that 50% of candidate variants we shortlisted using the assembly-based alignment approach were not called based on the classical approach of aligning long reads, either entirely or by calling the wrong zygosity (Fig. 5g). We checked that the assembly-based variants occurred in regions of high-quality assembly as indicated by Flagger. Furthermore, when we repeated the exercise of trio analysis based on variants obtained using the classical read-based approach, we shortlisted seven de novo variants for the six participants (Supplementary Table 3). We found that two of those were already detected using an assembly-based approach, including the SNV variant for *NFIX*, while the others were not valid de novo upon further examination of the read alignments, since they also appeared in the parental genomes as illustrated for the variants in *TRPM3*, *AKT3* and *KMD4B* (Supplementary Fig. 24). Notably, that became clearer when the alignments were done against closer references, as illustrated by aligning Syrian participants against Jordanian father or HG002 (Supplementary Fig. 24).

### Value of a ME population reference

**Fig. 6:**
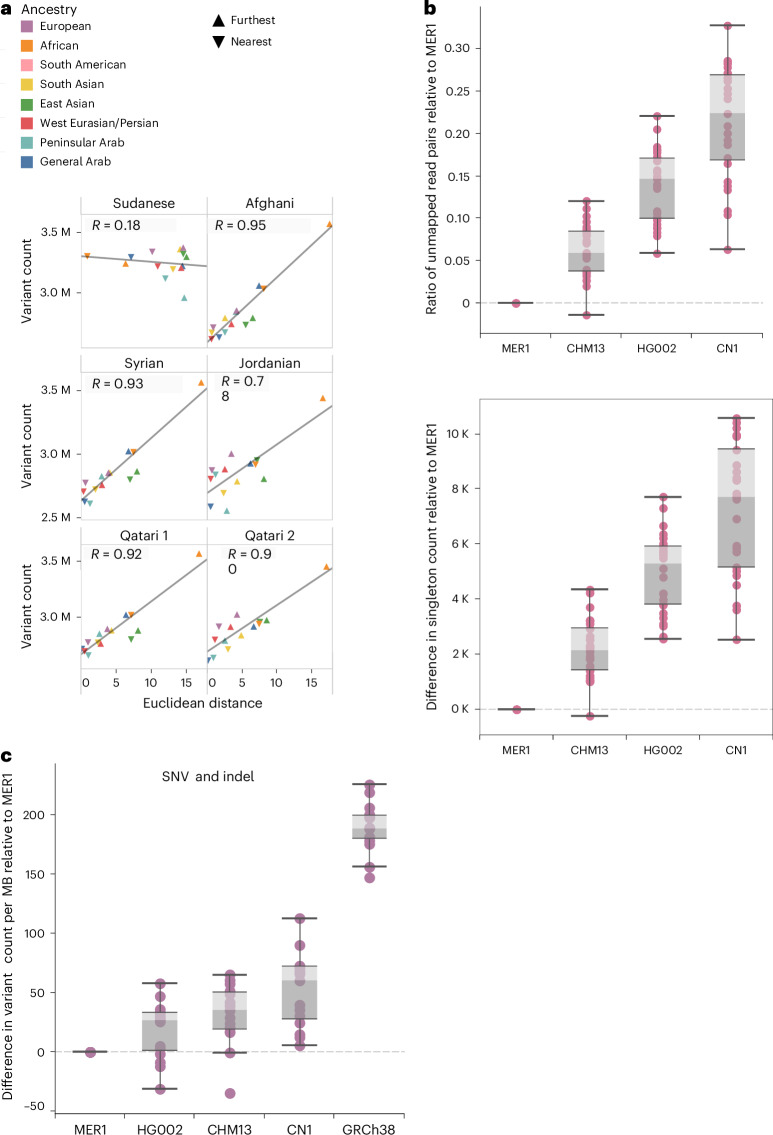
Variant calling and mappability against MER1 and other references. **a**, Euclidean distance versus variant count for each of the child assemblies. Color indicates the ancestry of the test samples. The shape of the markers differentiates the samples with maximum and minimum distance from our assembly for a given ancestry. Regression lines and coefficients of the Pearson correlations are shown. Corresponding *P* values are <10^−4^ for all except Sudanese (*P* = 0.54), calculated using two-sided *t* test. **b**, Ratio of unmapped read pairs over mapped and number of singletons relative to MER1 in the replacement chromosomes for ME query samples (*n* = 15) for various reference genomes. Values were calculated per 1 Mb regions and averaged over chromosomes. **c**, Differences in variant counts per 1 Mb for ME query samples (*n* = 15) from various ME ancestries against various reference genomes relative to MER. Source data

The assemblies generated in this study allow us to assess the impact of a ME genome reference. First, to illustrate the relationship between genetic distance to the genome reference and the number of called variants, we aligned test samples with diverse ancestries from 1KG and ME populations against each of the ME child assemblies. For each assembly-ancestry combination pair, we used two test samples per population as follows: one closest and one furthest from the assembly, as identified based on principal component analysis (PCA). A significant linear correlation was observed between the number of variants and the Euclidean distance to the test samples for all assemblies except the Sudanese (*R* = 0.18; Fig. 6a).

Next, to specifically compare the performance of a ME reference relative to the published T2T references controlling for completeness, we examined the impact on read mappability and variant calling of SNVs and SVs from aligning 27 short-read test samples with diverse ME Arab ancestries encompassing PAR, GAR and WEP against GRCh38, CHM13, HG002 and MER, a hybrid assembly based on CHM13 in which we replaced six chromosomes with diverse T2T haplotypes from Qatari 1 (chromosomes 4 and 18), Jordanian (chromosomes 10 and 12), Syrian (chromosome 6) and Qatari 2 (chromosome 11), which we denote as MER1. In terms of read mappability against the six chromosomes, MER1 shows a subtle but consistent trend of improved metrics relative to CHM13, HG002 and CN1 in this order (Fig. 6b). The difference in the normalized ratio of unmapped reads pairs over mapped relative to MER1 is highest for CN1 (median = 0.22) and lowest for CHM13 (median = 0.06). The number of singletons follows a similar trend with CN1, showing 3.6-fold more singletons relative to MER1 than CHM13. On the other hand, the metrics show a prominent improvement relative to GRCh38 (Supplementary Fig. 25). Regarding variant calls, more variants per MB relative to MER1 are obtained against other references, with HG002 being the closest. Respectively, GRCH38, CN1 and CHM13 show 7.1-, 2.3- and 1.3-fold more median variant counts in comparison to HG002 (Fig. 6c).

## Discussion

With the increasing ability to produce high-quality genome assemblies at a larger scale, generating population genome references has become more compelling than ever. However, the challenge remains in the strategy for sampling diverse admixture. While the HPRC pangenome included many East Asian genomes, the CPC pangenome revealed substantial diversity outside HPRC^4,5^. ME populations, with their distinct genetic structures and history, require more refined sampling methods. Our findings show varied admixture patterns linked to geographical dispersal, even among populations sharing language and culture. Thus, future efforts to construct accurate population references must consider historical and anthropological factors, instead of solely relying on modern delineations of regions and territories.

The use of parental information has long been considered the gold standard for phasing genome assemblies^43^; however, new methods relying on methylation, Hi-C and Strand-seq have substantially improved phasing quality for individual samples^44–46^. Nevertheless, in endogamous/consanguineous populations, parental phasing remains essential in resolving the extensive homozygosity. Trio datasets like the one presented here will help in developing population-specific recombination maps facilitating larger-scale phasing.

Our assemblies are nearly complete compared to CHM13, revealing 7–11 Mb of new sequences per participant, consistent with previous reports^47^. Some regions, such as centromeres and acrocentric p-arms, are not necessarily missing; however, they are difficult to align due to their divergence and ectopic recombination. Ninety percent of new sequences are in repetitive intergenic regions and the remaining are enriched for gene elements. Gene coverage is high with some decrease for rRNA genes, known to have multiple copies^28^. We identified 75 new HLA/KIR alleles in the assemblies, including 11 with CDS mutations. Four alleles were unique to Sudanese ancestry, consistent with its greater African genetic divergence. We map these alleles’ phylogenetic context, which will serve as a valuable addition to understanding HLA/KIR diversity and their role in immune-mediated processes across populations^48^.

Soon, assembly-based methods are expected to replace read-based methods for alignment and variant calling, leading to higher accuracy^49,50^, especially when the query is divergent from the genome reference. This is highlighted by our comparison of DeepVariant (read based)^51^ and phased assembly variant caller (PAV) (assembly based)^52^. The ME assemblies exhibited 6.6% more variants against CHM13 than GRCh38, consistent with CHM13 having 8% more sequence than GRCh38 (ref. ^6^). We observed fewer SV deletions and more inversions against GRCh38, reflecting known imbalances^8^ and misorientations in the latter^9,53^. Nearly half of the new SVs in ME genomes span intronic and exonic regions, with a balanced distribution between repetitive and non-repetitive regions, suggesting a broad biological impact.

We show that assembly-based variant calling, especially with T2T references like CHM13, enhances variant detection and inheritance analysis in rare disease trios^54^. However, while this improves the identification of disease-causing variants, it may not immediately increase genetic diagnosis yield due to limited functional annotation, especially in non-coding regions^55^. Intronic variants are increasingly implicated in rare diseases^56^ but are often classified as VUS under current ACMG criteria due to the lack of algorithms scoring complex non-coding variants^57^. This makes functional validation a bottleneck for confirming many genetic diagnoses. As sequencing advances near-complete variant landscapes, large-scale genome annotation efforts—such as the IGVF consortium^58^—are urgently needed for systematic interpretation and prioritization.

Phased genome assembly allows more accurate ROH calculation, essential when studying consanguineous populations. As expected, we observe high ROH levels in the ME trios, especially long ROH, indicative of recent inbreeding^32,33^. This includes extended ROH in healthy individuals, leading to the autozygosity of many genes, uncovering extensive benign variation. Also, it harbors recessive deleterious variants contributing to disease phenotypes^16^. While de novo variants are a major cause of developmental disorders^59^, recessive variants, as shown here, are relevant in consanguineous populations, and multiple variants can co-occur, explaining comorbidities and variable penetrance^60^.

Finally, we demonstrated that using ME assemblies as references reduces variant calls and improves mappability compared to unmatched references, highlighting the need for population-specific genome references to capture relevant variations, including pathogenic ones. As more disease cohorts undergo long-read sequencing and de novo assembly, these data could enhance pangenome efforts with informative haplotypes as long as they lack gross chromosomal abnormalities. While there is a need for a universal pangenome reference cataloging global variation^61^, its wide adoption remains challenging. That is also compounded by the shift toward direct genome comparisons using multiple alignments. Nevertheless, integrating ME pangenomes and other regional datasets would greatly enrich the genomic and biomedical landscape.

## Methods

### Study samples

The study participants consist of six parent–child family trios (*n* = 18) from the local population in Qatar recruited at Sidra Medicine, whereby children were ascertained for neurodevelopmental disorders. Previously, three families (Sudanese, Jordanian and Qatari 2) had negative genetic diagnoses based on microarrays and selected panels.

The families are from various nationalities from the greater ME region—Sudan, Jordan, Syria, Qatar (2X) and Afghanistan. Whole peripheral blood samples were collected from the individuals, stored in EDTA tubes at −80 °C and subsequently processed for DNA and RNA sequencing as described below.

Written informed consent and assent were obtained from all participants, and the study was approved by the Sidra Medicine Ethics Committee.

### Sequencing data generation and quality assessment

Whole-genome Illumina short-read sequencing was performed at Sidra Medicine. First, genomic DNA was isolated from blood samples on the QIAsymphony System (QIAGEN) using the DSP DNA Midi Kit, per the manufacturer’s instructions. DNA quantity and quality were assessed using NanoDrop 8000 (Thermo Fisher Scientific); the absorbance at 260 and 280 nm wavelengths was used to check DNA purity. A fluorescence-based quantification was performed on FlexStation 3 (Molecular Devices) using Quant-iT PicoGreen dsDNA Assay (Thermo Fisher Scientific). DNA integrity was checked on LabChip GX (PerkinElmer). Next, 150 bp paired-end read libraries were constructed and sequencing was performed on a HiSeq X System at Sidra Medicine at 30× coverage. QC was done using FastQC^62^ and MultiQC^63^. All reads that had more than an average of 450 million unique paired-end reads were retained. SAMtools v1.17 (ref. ^64^) and Mosdepth v0.3.2 (ref. ^65^) were used to calculate coverage and number of mapped reads.

PacBio HiFi sequencing was performed at the University of Washington, as previously described^66^ with some modifications. Briefly, high-molecular-weight DNA was extracted from the flash frozen peripheral blood samples using the QIAGEN MagAttract HMW DNA Kit (67563). After quality and quantity checks (Agilent FEMTO Pulse, M5330AA and FP-1002-0275; Denovix, DS-11 FX; Thermo Fisher Scientific Q32854), DNA was sheared on Megaruptor 3 (Diagenode, B06010003 and E07010003) twice using settings 29/31 to obtain a peak size around 22 kb. SMRTbell library preparation with the SMRTbell Express Template Prep Kit 2.0 (PacBio, 100-938-900) and size selection on Sage Science PippinHT (HPE7510) were performed as previously described in ref. ^66^. Next, sequencing was performed using 3–4 SMRT cells on the Sequel II platform with chemistry version 2.2, 30-h movie times and 2 h pre-extension (PacBio, 101-894-200), leading to coverage of 30–50× per sample. Data quality was evaluated using SMRTlink v.10.1 QC reports from which we used the awk v4.0.2 {print [field]} command to extract the fields relating to sequencing quality, read length distribution and coverage. CCS reads with QV ≥ 20 were retained for subsequent analysis.

### Genome assembly and quality assessment

de novo genome assembly was conducted by running the trioHiFiasm pipeline^23^ (hifiasm -o sample.asm -t32 -1 pat.yak -2 mat.yak HiFisample.fq.gz), using the parental short reads to create k-mers generated using yak/0.1 (yak count -k31 -b37 -t threads -o output.parental_yak) to construct a pair of haplotype-resolved phased assemblies. The fasta files for the assembly pipeline was generated from gfa files using awk v4.0.2 (https://www.gnu.org/software/gawk/).

Various assembly quality metrics were calculated on the haploid assemblies using stats.sh script in BBmap (v38.69)^67^. Phasing quality was evaluated using Merqury v1.3 (ref. ^24^) by examining blob plots, switch error rates and phased blocks to ensure completeness, accuracy and reliability. The QV score was used to quantify phasing accuracy and completeness. Telomer count checks were made using seqtk telo^68^, confirming the presence of telomere repeats (TTAGGG) at chromosomal ends of the assemblies.

HiFi read-based evaluation of the assemblies was performed by running the Flagger v0.3.3 pipeline following the standard workflow as previously described^4^. For each assembly, it classifies segments of the assembly sequence into five categories, namely Haploid (reliable), unknown, duplicated, collapsed and erroneous.

### Assembly alignment to GRCh38 and CHM13

Assembly-to-assembly alignment was conducted using Minimap2 (ref. ^25^) with options -x asm5 -c, which produced paf files with CIGAR string. To allow visualization with SafFire^69^, the paf files were converted to SafFire format using rustybam v0.1.31 (ref. ^70^) with the command *r*b break-paf --max-size 5000 | rb orient | rb filter --paired-len 100000 | rb stats –paf. The SafFire files were uploaded to an internally deployed SafFire server, which provided an interface to visualize the alignments of haplotypes to GRCh38 and CHM13 (v2.0).

### Variant calling

For the variant calling in ME assemblies relative to GRCh38 and CHM13, we used two approaches: One based on HiFi read alignments and the other on assembly-based alignments. For the first approach, pbmm2 1.13.0, a wrapper for minimap2 (ref. ^25^) was used to generate the alignment and DeepVariant v1.5.0 (ref. ^51^) was used to call both SNVs and indels, while PBSV was used to call SVs^71^. For the second approach, PAV v2.3.3 (ref. ^52^) was used to do both the alignment and calling of SNVs, indels and SVs including insertions, deletions (≥50 bp) and inversions.

For variant calling of various reference samples from the 1KG and QGP against the assemblies, Illumina short reads were aligned using BWA-MEM^72^ and DeepVariant v1.5.0 (ref. ^51^) was used to call SNVs and indels. Variants with the PASS filter were retained for downstream analysis.

### PCA

PCA was performed using SNVs from a dataset combining the 18 study participants, 2,504 samples from the 1KG phase 3 dataset^36^ and 6,216 samples from the QGP phase 1 (refs. ^14,37^) in addition to an internal diverse set of ME participants at Sidra Medicine (*n* = 1,693). For the published datasets, SNVs with the PASS filter were used. For the study participants, SNVs were obtained from the Illumina data by aligning to GRCh37 and calling the variants using the GATK best practice workflow^73^, retaining only SNVs with the PASS filter. The combined dataset was generated using BCFtools isec v1.17 (ref. ^64^) and contained 21.3 million SNPs. We applied a minor allele frequency (MAF) filter with a 0.01 threshold, resulting in 7.9 million variants from which principal components were calculated using PLINK v2.00a2LM 64-bit Intel (7 January 2019)^74^.

### Global and local genetic ancestry assignment

RFMix v2.0.0 (ref. ^75^) was used to assign global and local ancestry for the genomes of each study participant based on VCF file containing Illumina-derived SNPs and indels, using as reference a diverse panel from the 1KG and QGP phase 1 cohort which consists of 176 representative individuals identified as having a dominant ancestral fraction >90% for eight distinct ancestral fractions^14^. This was derived from Admixture analysis of 6,216 samples from QGP and 2,504 samples from 1KG^14^, ensuring diversity encompasses various continental populations including various ME groups. Output from RFMix consisted of ancestry estimates for chromosomal painting as well as global ancestry estimates (.Q file). RFMix requires phased genotypes, therefore, these were generated using EAGLE v2.4.1 (ref. ^76^), which was run with three iterations and by providing genetic map and default parameters.

### New sequences

Insertions from PAV alignment for the assemblies against CHM13 (2.0) were collapsed per participant using truvari^77^ *(*truvari collapse -r 500 -p 0.95 -P 0.95 -s 50 -S 100000). Unique sequences were identified by comparing with the cohort of 47 samples from HPRC for which SVs were previously called^8^ using truvari (truvari bench -r 1000 -C 1000 -O 0.8 -p 0.8 -P 0.0 -s 50 -S 15 --sizemax 100000). An 80% reciprocal overlap was used. In addition, insertions from various participants were also collapsed into a unique set of sequences using the same process starting from collapsed insertions per participant. Subsequently, the resulting sequences were checked similarly for overlap with HG002 (v. 1.0.1) and CN1.

### SV annotation with gene elements, repetitive regions and segmental duplications

We used bedtools v2.30 (ref. ^78^) with default options including overlap threshold of 1 bp to intersect SVs with bed files obtained from the CHM13 public resource, namely genetic features (https://ftp.ncbi.nlm.nih.gov/genomes/all/GCF/009/914/755/GCF_009914755.1_T2T-CHM13v2.0/GCF_009914755.1_T2T-CHM13v2.0_genomic.gtf.gz), repetitive regions (https://s3-us-west-2.amazonaws.com/human-pangenomics/T2T/CHM13/assemblies/annotation/chm13v2.0_RepeatMasker_4.1.2p1.2022Apr14.bed), segmental duplications (https://s3-us-west-2.amazonaws.com/human-pangenomics/T2T/CHM13/assemblies/annotation/chm13v2.0_SD.full.bed), centromeric and satellite repeats (https://s3-us-west-2.amazonaws.com/human-pangenomics/T2T/CHM13/assemblies/annotation/chm13v2.0_censat_v2.1.bed).

### Gene annotation

To annotate assemblies with known genes, we used Liftoff v1.6.3 (ref. ^79^), using NCBI Refseq GTF file for CHM13 (https://github.com/marbl/CHM13?tab=readme-ov-file) with default parameters. The pLI scores for individual genes were obtained from gnomAD^35^ (https://gnomad.broadinstitute.org/downloads#v4-constraint).

### Typing of HLA and KIR alleles and generation of phylogenetic trees

We used Immuannot to annotate the alleles of HLA and KIR genes per genome haplotype^29^. We typed the alleles of these genes using default options and extracted CDS mutations and CDS distance for new gene alleles. The results were compared with previously published annotations of 220 pre-annotated reference haplotypes, including HLA assemblies and those from HPRC, CPC, CHM13, CN-T2T and GRCh38.p14 downloaded from https://zenodo.org/records/8372992.

Phylogenetic trees were generated by first extracting the DNA sequences of each immune gene from the study assemblies using SAMtools v1.17 (ref. ^64^). These sequences were combined with the publicly available reference sequences into a single FASTA file per gene. This was used as input to generate a multiple-sequence alignment and a neighbor-joining phylogenetic tree using Clustal Omega^80^. The trees were plotted using TreeViewer^81^. Sequences were retrieved from the IPD^48^. Alignment figures were generated using Jalview^82^.

Allele frequencies for *HLA-A* alleles were obtained from the CIWD catalog v3.0 (ref. ^30^). Population ancestry of source samples for the closest alleles to the new alleles identified in the assemblies was obtained for the IPD^48^.

### ROH

ROH segments were identified per participant using Automap v1.2 (ref. ^83^) using default parameters and variant calls from PAV for the assemblies against CHM13 as input. GTF file was also provided as input, which resulted in the annotation of ROH segments using genes. A selection of ROH regions was visually verified using Integrative Genomics Viewer (IGV)^84^.

### Family trio analysis and variant prioritization

Using SNVs, indels and SVs produced from the trios called against CHM13, we perform trio-based analysis for each family following the workflow of Exomiser v14 (ref. ^85^), which itself does not support the CHM13 reference. This method combines variant prioritization and phenotypic concordance^86^. Given that the disease symptoms affect the children only, we considered the modes of inheritance of recessive and de novo. First, we shortlisted variants that met each mode of inheritance and then did a lift over to GRCh38 coordinates followed by running annotation using Jannovar^87^ and annotSV v3.4.2 (ref. ^88^) to retrieve various functional scores and allele frequencies from various public databases including allele frequencies from various public databases including gnomAD v4.0 (ref. ^35^), dbVar and 1KG^36^, to which we appended allele frequencies from Qatar Genome Project Phase 1 (refs. ^14,37^). For the SV annotation with annotSV v3.4.2 (ref. ^88^), we adopted minimum 80% reciprocal overlap to account for breakpoint imprecision. We selected variants with AF < 0.01 in various population databases and have genotype–phenotype association *P* vaue of <0.05 and a gene combined score and genotype–phenotype score of >0.8. Further filtering was performed based on variants with a high functional consequence. Finally, we cross-checked in ClinGen^38^, DECIPHER^38^ and Gene2Phenotype^39^ to confirm the relevance of the link obtained to the proband’s condition. We checked that variants were in regions flagged as reliable by Flagger v0.3.3 (ref. ^4^).

We performed variant classification for SNVs and Indels based on the guidelines recommended by ACMG^89^, and for SVs based on the joint recommendations by ACMG and ClinGen^90^ and as implemented in Franklin (https://franklin.genoox.com/clinical-db/home). For the latter, we classified variants as pathogenic, likely pathogenic and VUS if they have total ACMG scores ≥ 0.99, 0.90–0.98 and −0.89 to 0.89, respectively.

### Calculation of metrics of read mappability

To assess read mappability against various genome references, we selected 27 representative samples with diverse ME Arab ancestries encompassing PAR, GAR and WEP for which Illumina reads (30×) were mapped against each reference using DRAGEN^91^. Metrics of the ratio of unmapped read pairs over mapped, percentage of properly paired reads and number of singletons were calculated based on values obtained from the output of DRAGEN and were tabulated to generate the various plots.

### Statistics and reproducibility

No statistical method was used to predetermine sample size, but our sample sizes are typical of those used in the field of study. No data were excluded from the analyses. The experiments were not randomized. Investigators were not blinded to allocation during experiments and outcome assessment. Pearson correlation with two-sided significance was calculated. The tools and packages used for the processing and analysis of data in this study have been reported in the Methods and Study summary, with the specific versions allowing the reproducibility of the results. In all box plots in this study, the boxes indicate the middle 50% of the data, while whiskers indicate 1.5 times the interquartile range. The lower quartiles are dark shaded and the upper ones are light shaded.

### Reporting summary

Further information on research design is available in the Nature Portfolio Reporting Summary linked to this article.

## Online content

Any methods, additional references, Nature Portfolio reporting summaries, source data, extended data, supplementary information, acknowledgements, peer review information; details of author contributions and competing interests; and statements of data and code availability are available at 10.1038/s41588-025-02173-7.

## Supplementary information

Supplementary Information Supplementary Figs. 1–25.

Reporting Summary

Supplementary Tables Supplementary Tables 1–3.

## Source data

Source Data Fig. 1 Statistical source data.

Source Data Fig. 2 Statistical source data.

Source Data Fig. 3 Statistical source data.

Source Data Fig. 4 Statistical source data.

Source Data Fig. 5 Statistical source data.

Source Data Fig. 6 Statistical source data.

Source Data Extended Data Fig. 2 Statistical source data.

Source Data Extended Data Fig. 3 Statistical source data.

Source Data Extended Data Fig. 4 Statistical source data.

## Data Availability

Sequencing data and assemblies generated in this study are available for general research use through controlled access at dbGAP to preserve patient confidentiality (accession ID phs003917.v1.p1; https://www.ncbi.nlm.nih.gov/projects/gap/cgi-bin/study.cgi?study_id=phs003917.v1.p1). Previously published data used in the study are accessible as follows: 1000 Genomes Project https://hgdownload.cse.ucsc.edu/gbdb/hg19/1000Genomes/phase3 CHM13 assembly https://s3-us-west-2.amazonaws.com/human-pangenomics/T2T/CHM13/assemblies/analysis_set/chm13v2.0.fa.gz CHM13 annotations https://ftp.ncbi.nlm.nih.gov/genomes/all/GCF/009/914/755/GCF_009914755.1_T2T-CHM13v2.0/GCF_009914755.1_T2T-CHM13v2.0_genomic.gtf.gz https://s3-us-west-2.amazonaws.com/human-pangenomics/T2T/CHM13/assemblies/annotation/chm13v2.0_RepeatMasker_4.1.2p1.2022Apr14.bed https://s3-us-west-2.amazonaws.com/human-pangenomics/T2T/CHM13/assemblies/annotation/chm13v2.0_SD.full.bed https://s3-us-west-2.amazonaws.com/human-pangenomics/T2T/CHM13/assemblies/annotation/chm13v2.0_censat_v2.1.bed CIWD v3.0 catalog https://www.ihiw18.org/component-immunogenetics/download-common-and-well-documented-alleles-3-0/ CN1 assembly https://genome.zju.edu.cn/files/v1.0.1/CN1_pat.v1.0.1.fasta.gz https://genome.zju.edu.cn/files/v1.0.1/CN1_mat.v1.0.1.fasta.gz gnomad v4.1.0 https://gnomad.broadinstitute.org gnomad gene constraint https://storage.googleapis.com/gcp-public-data-gnomad/release/4.0/constraint/gnomad.v4.0.constraint_metrics.tsv GRCh38 assembly https://hgdownload.soe.ucsc.edu/goldenPath/hg38/bigZips/p13/hg38.p13.chromFa.tar.gz HG002 assembly https://s3-us-west-2.amazonaws.com/human-pangenomics/T2T/HG002/assemblies/hg002v1.0.1.fasta.gz immuannot IPD/KIR and CPC dataset https://zenodo.org/records/8372992/files/Data-2023Oct27.tar.gz?download=1 Qatar Biobank/Qatar Genome Project accession ID: QF-QGP-RES-PUB-007 (https://www.qatarbiobank.org.qa/research/how-apply). Source data are provided with this paper.

## Code availability

The study used previously published analysis tools as described in the Methods. No custom tools were used in this study.
