# Supplementary material for: Near-complete Middle Eastern genomes refine autozygosity and enhance disease-causing and population-specific variant discovery
Source: Nat Genet. 2025 May 5;57(5):1119–31. doi: 10.1038/s41588-025-02173-7 (PMC12081309; doi:10.1038/s41588-025-02173-7)

nature genetics

Supplementary information

https://doi.org/10.1038/s41588-025-02173-7

Near-complete Middle Eastern genomes refine autozygosity and enhance disease-causing and population-specific variant discovery

In the format provided by the authors and unedited

Contents

Supplementary Figures

Supplementary Fig. 1: HiFi read data QC metrics. **(a)** Number of SMRT cells used per sample. **(b)** Average HiFi read length per sample (bp) **(c)** Yield for each SMRT cell per sample

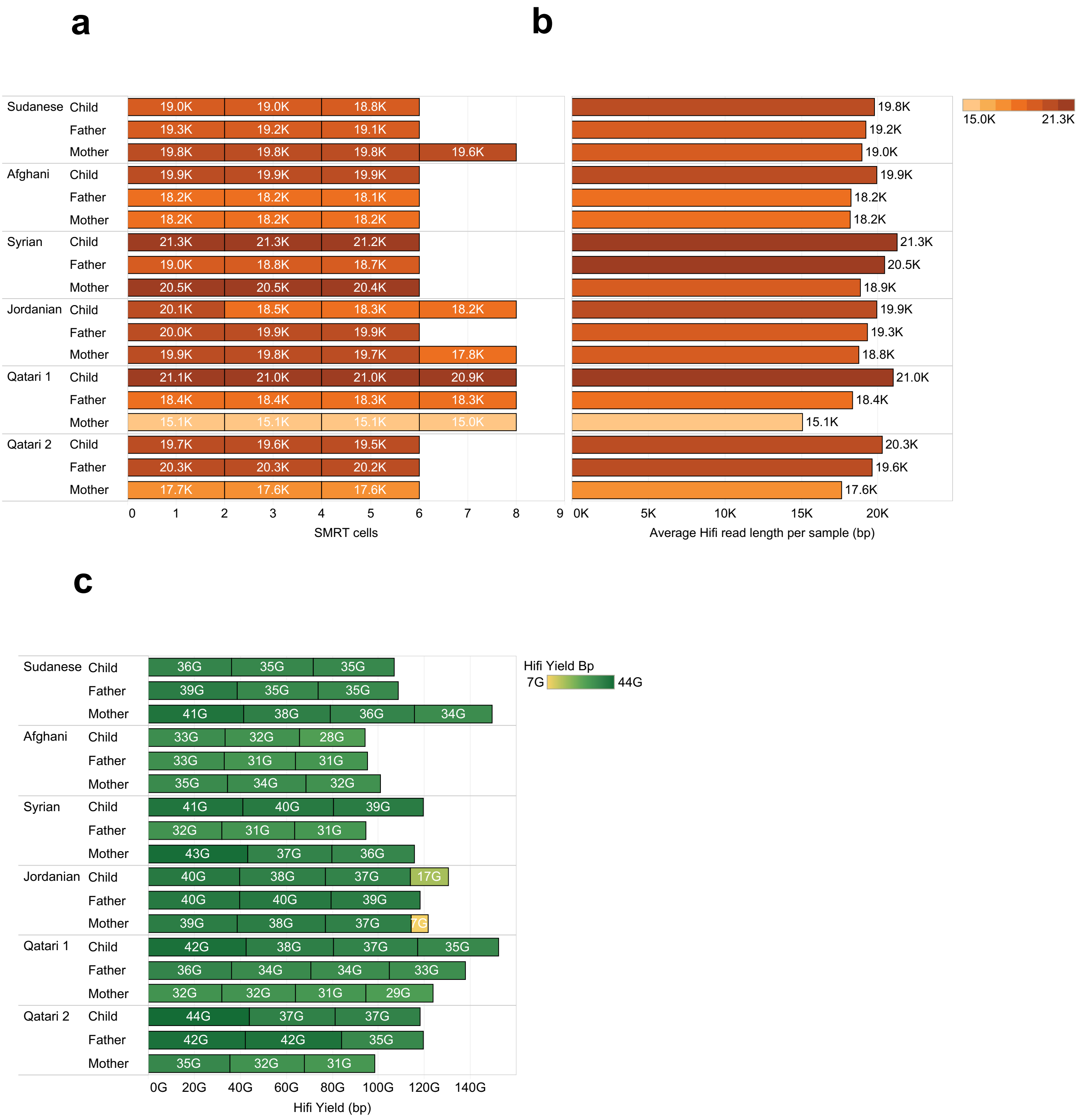

Supplementary Fig. 2: Illumina read data QC metrics. (a) Phred quality score distribution over the length of sequencing reads, indicating data quality per base position. **(b)** Distribution of the number of sequence tags over the read length, showing the consistency of tag presence across the length of the reads. **(c)** Read count and mean coverage per sample

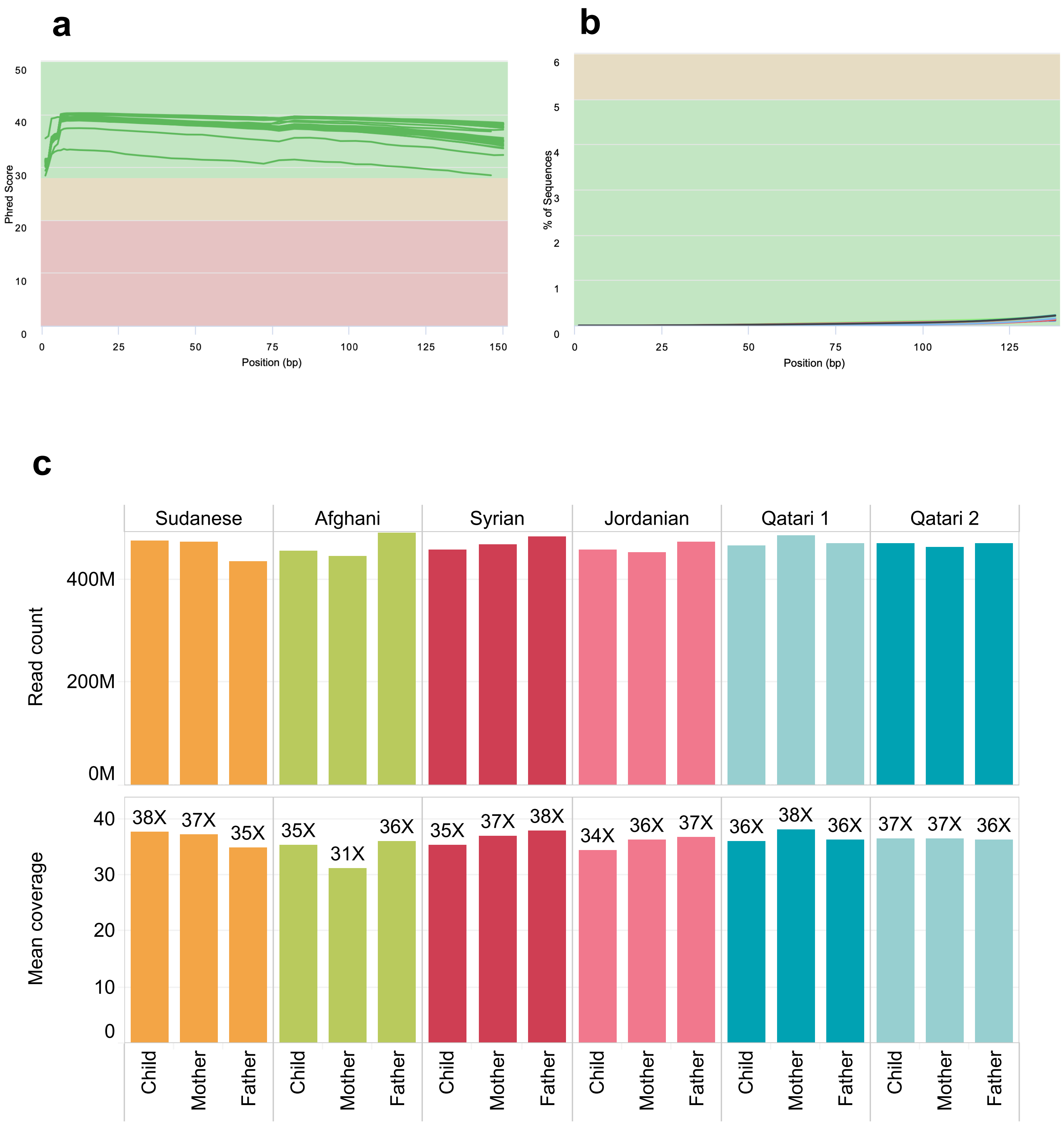

Supplementary Fig. 3: Local and global genetic ancestry of child subjects and other publicly available T2T genomes. (a) Local ancestry at chromosomal level for Syrian, Qatari 1, CHM13, HG002 and CN1. **(b)** Global ancestral assignments for all child study subjects

a

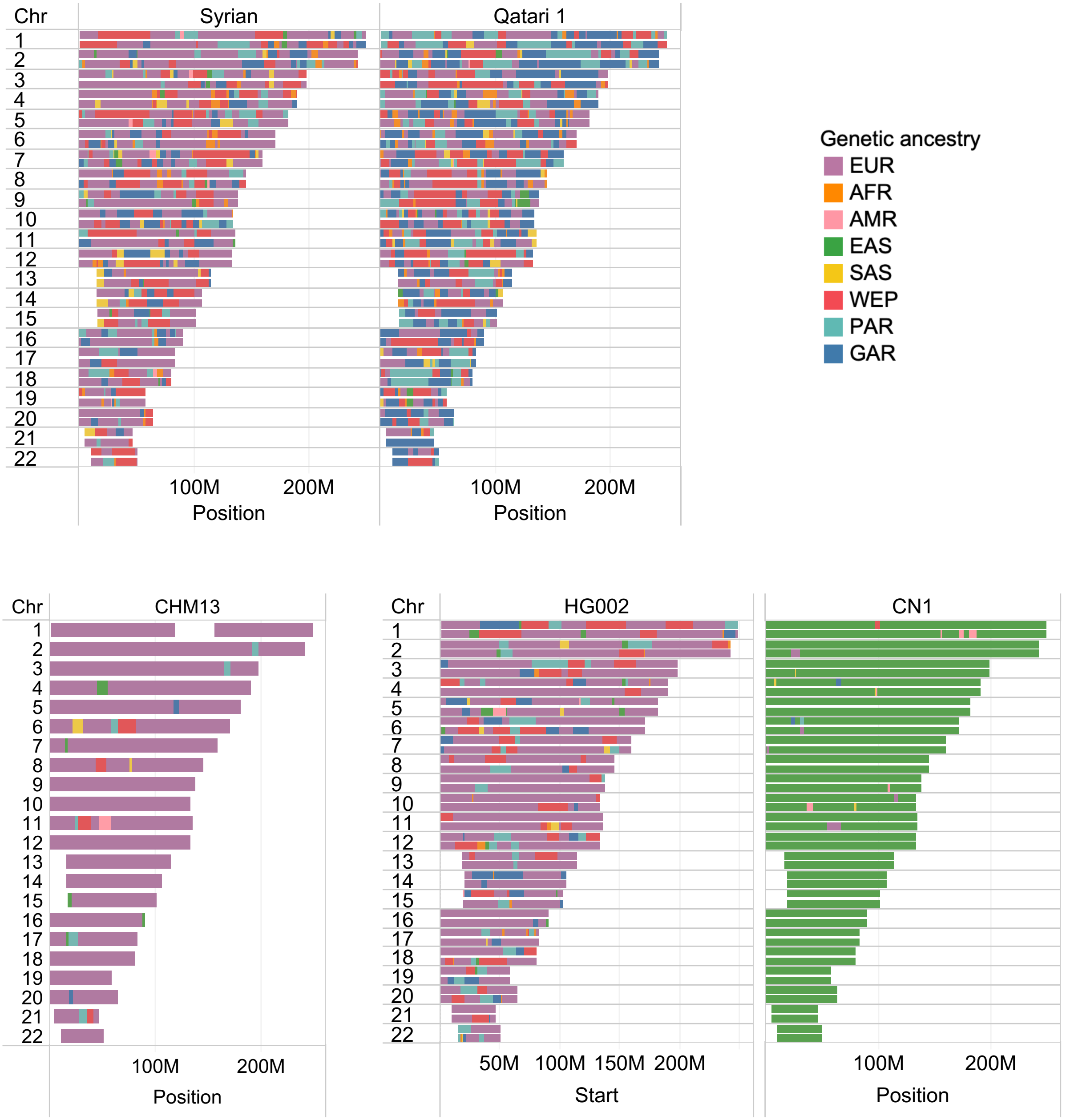

b

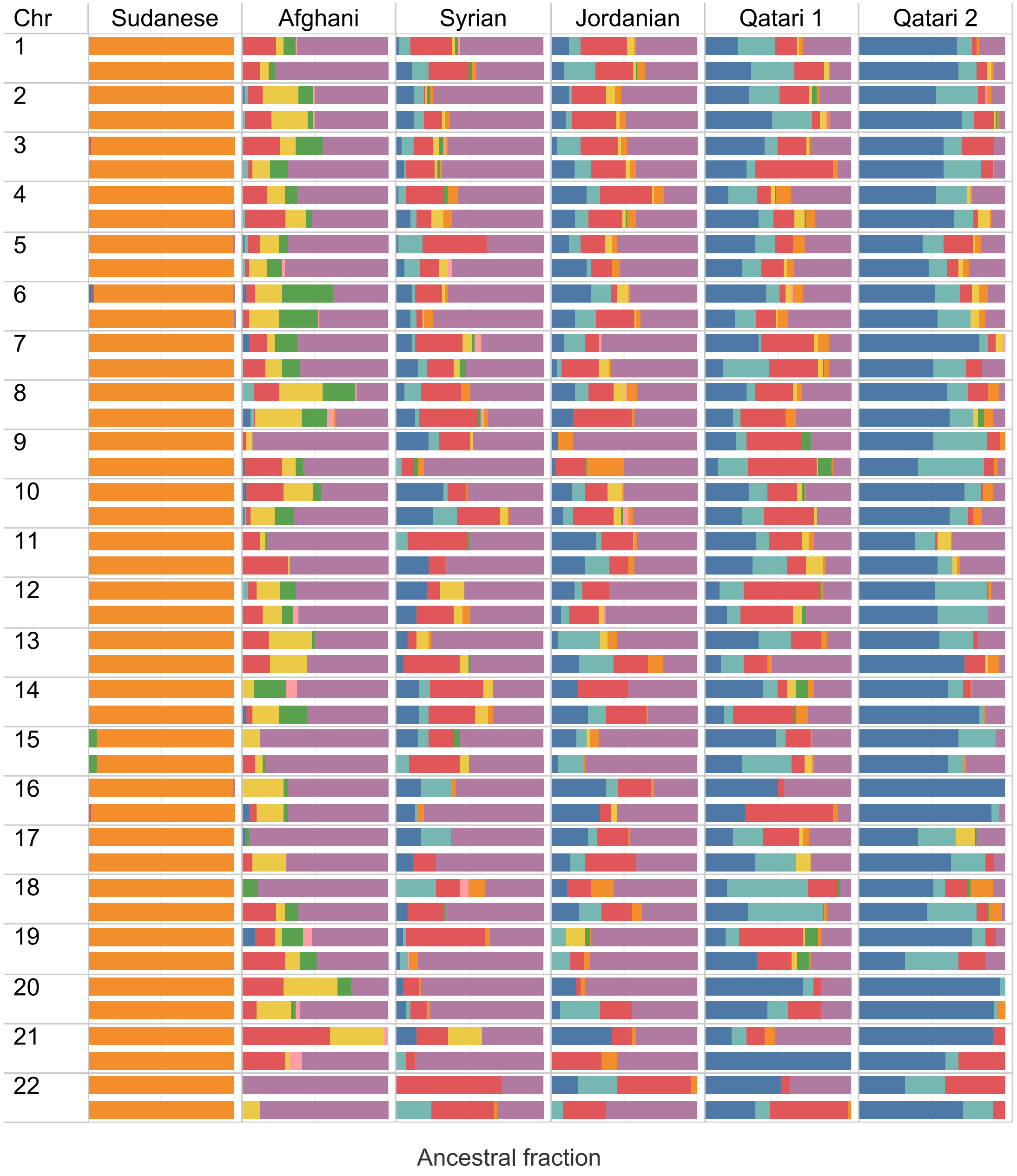

Chr
Sudanese
Afghani
Syrian
Jordanian
Qatari 1
Qatari 2
1
2
3
4
5
6
7
8
9
10
11
12
13
14
15
16
17
18
19
20
21
22
Ancestral fraction

Supplementary Fig. 4: Assembly results and QC. (a) Assembly QC of parental subjects, including coverage depth, total contigs, contig N50, maximum contig length and QV. **(b)** Comparison of QC metrics of child and parental assemblies.

a

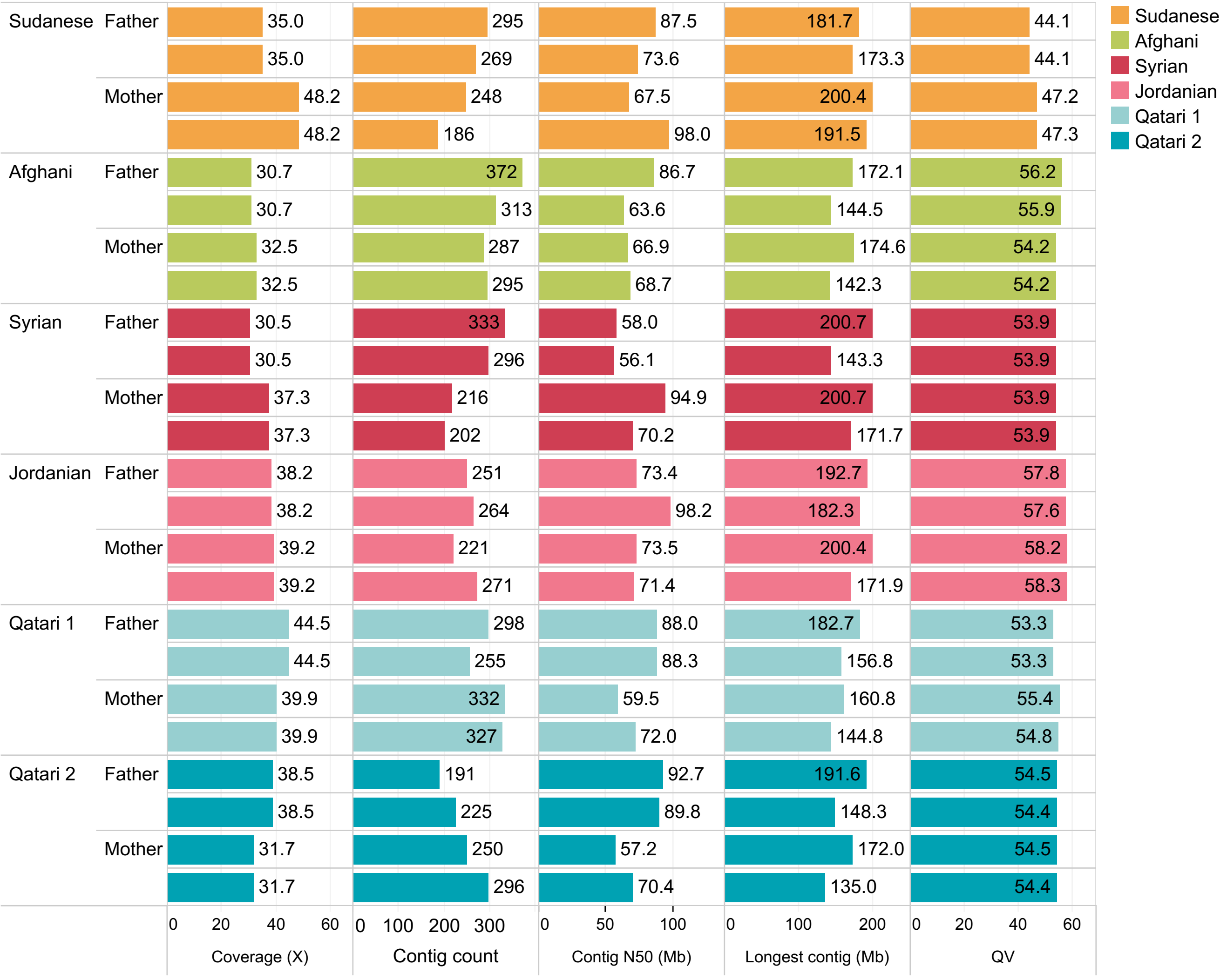

b

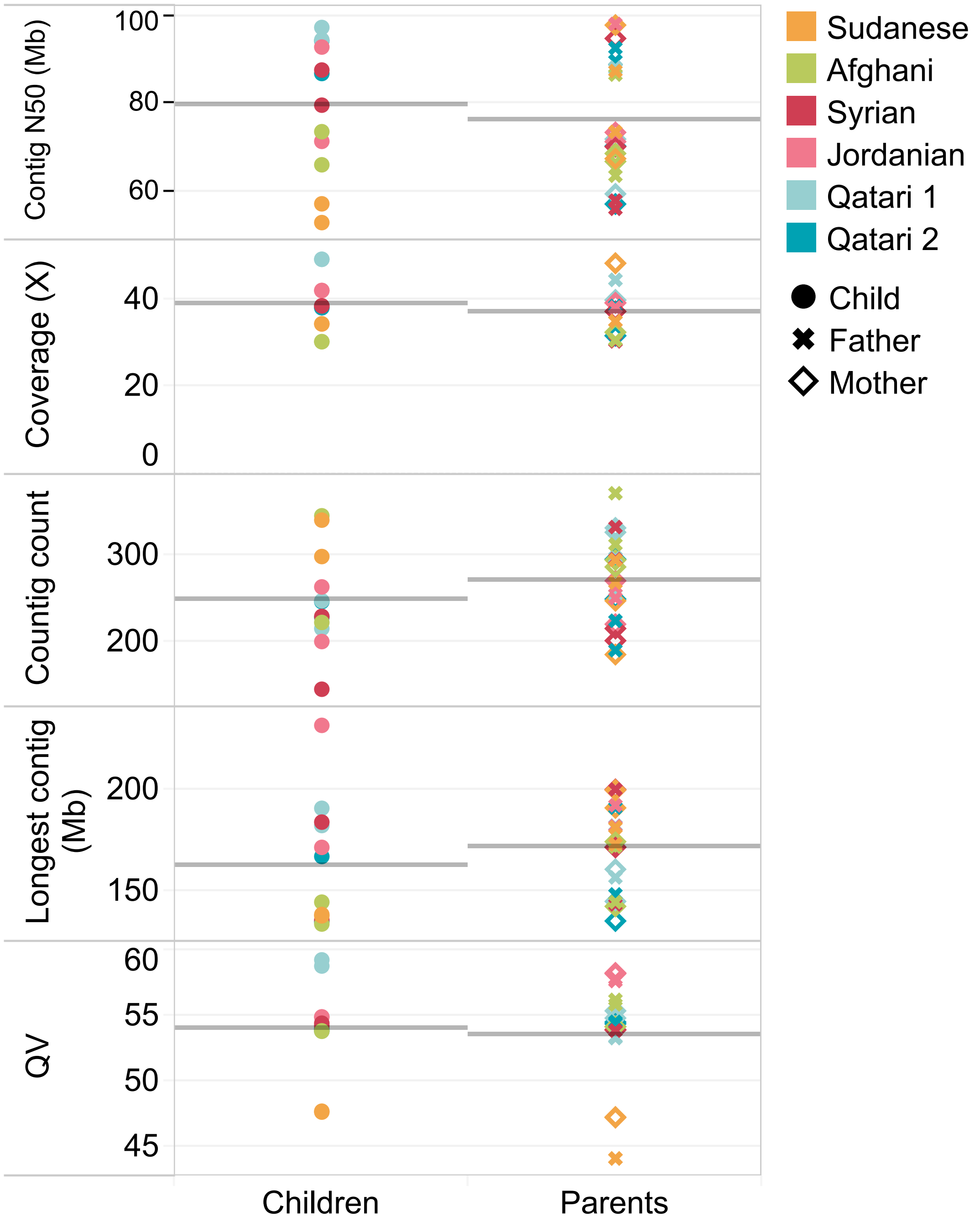

Contig N50 (Mb)
100
80
60
Coverage (X)
40
20
0
Countig count
300
200
Longest contig (Mb)
200
150
QV
60
55
50
45
Children
Parents
Sudanese
Afghani
Syrian
Jordanian
Qatari 1
Qatari 2
Child
Father
Mother

Supplementary Fig. 5: Hapmer blob plots for other child assemblies (Phasing QC). (a) Plots show separation of maternal (red) and paternal (blue) haplotypes, the blob size being proportional to contig size. **(b)** Complementary plot showing data for y axis > 2 M. Arrows point to the data points, for added clarity.

a

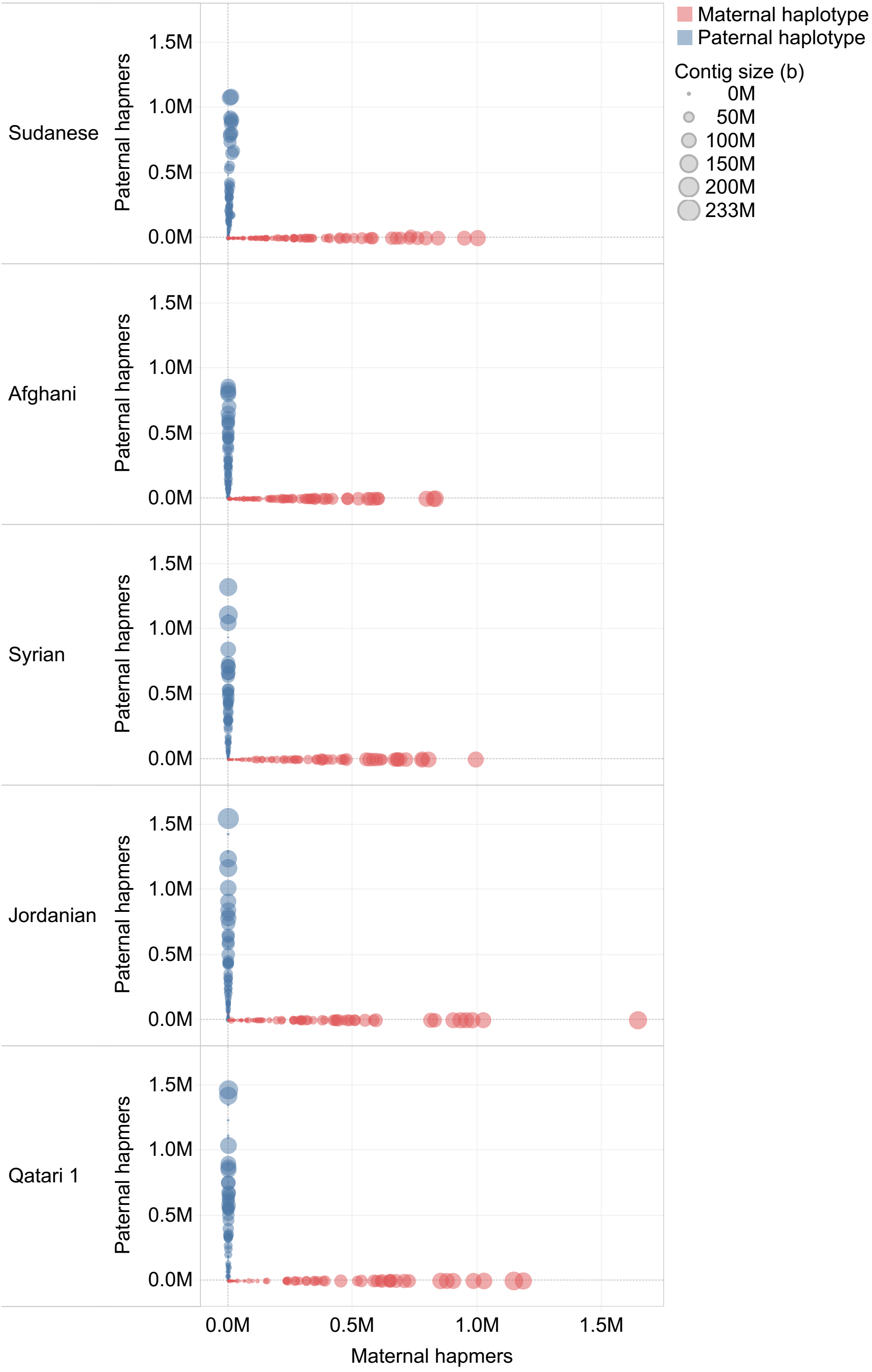

b

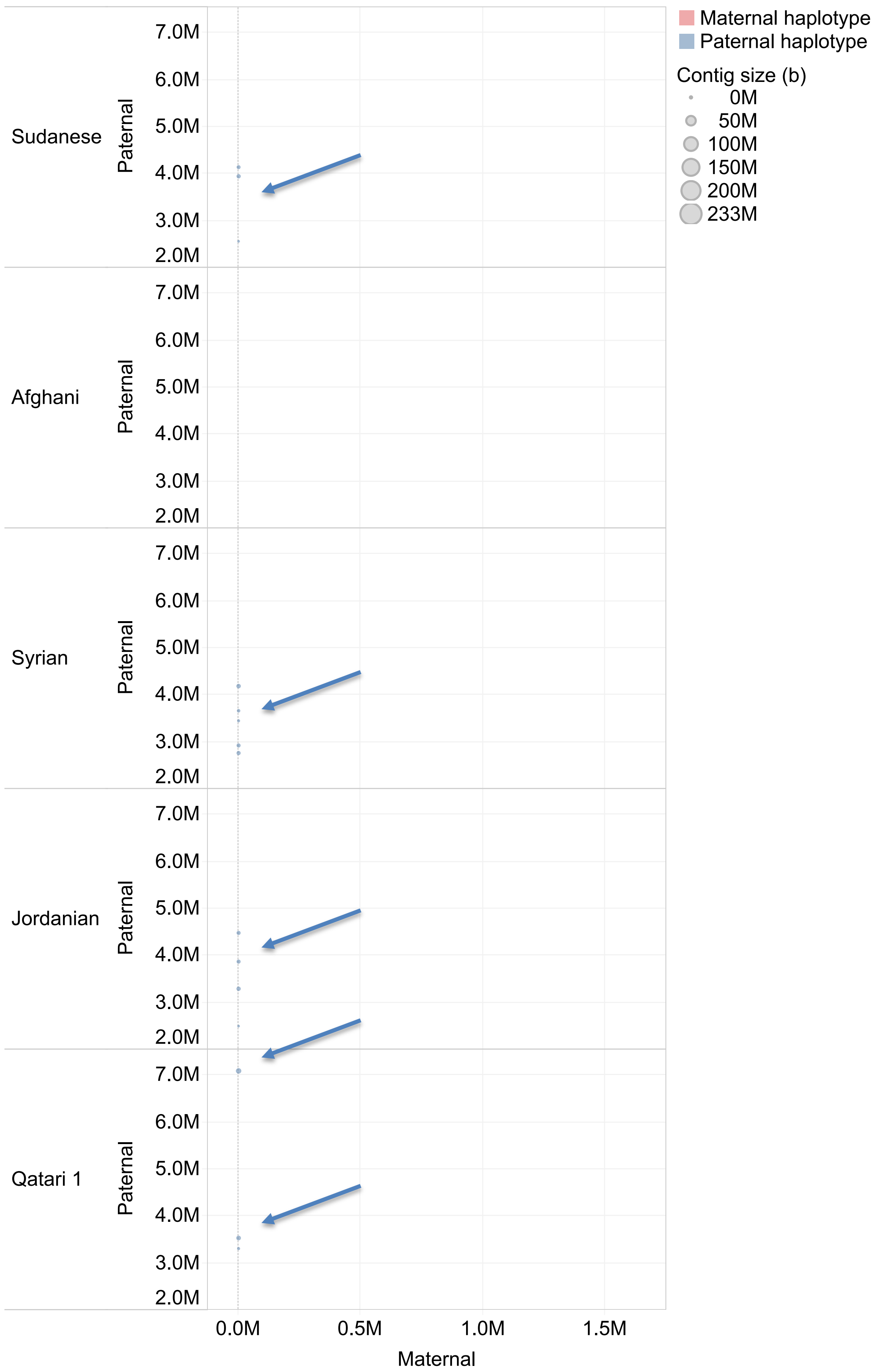
Maternal haplotype
Paternal haplotype
Contig size (b)
0M
50M
100M
150M
200M
233M
Sudanese
Afghani
Syrian
Jordanian
Qatari 1
Paternal
7.0M
6.0M
5.0M
4.0M
3.0M
2.0M
0.0M
0.5M
1.0M
1.5M
Maternal

Supplementary Fig. 6: Switch error plots for other child assemblies (Phasing QC). Phase block NG plots of haplotype-resolved assembly sorted by size.

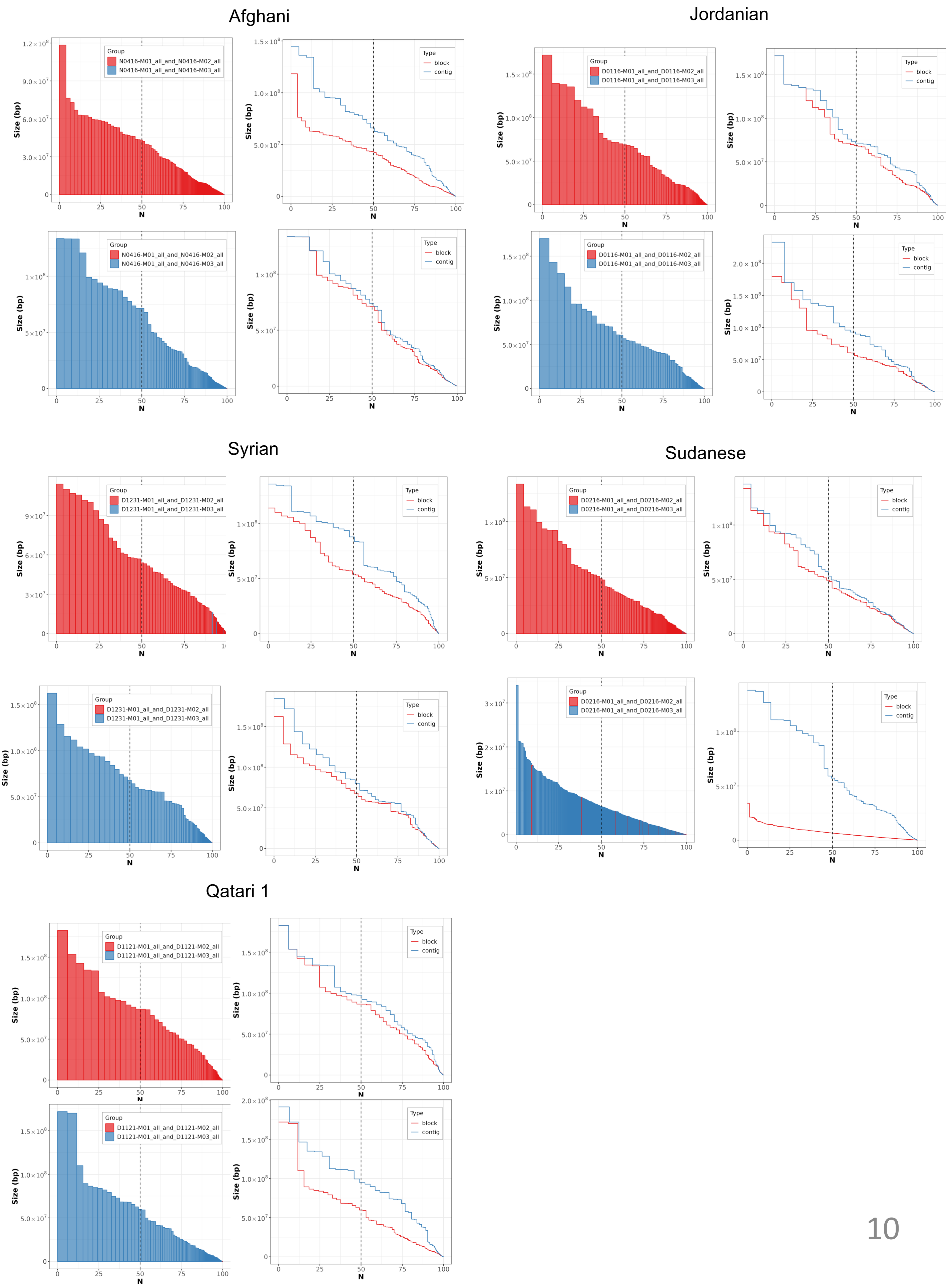

Supplementary Fig.7. Inherited hapmer plots for the child assemblies. k-mer multiplicity of the child read set colored by paternal (red) and maternal (blue) inheritance and those that are shared (green).

Afghani

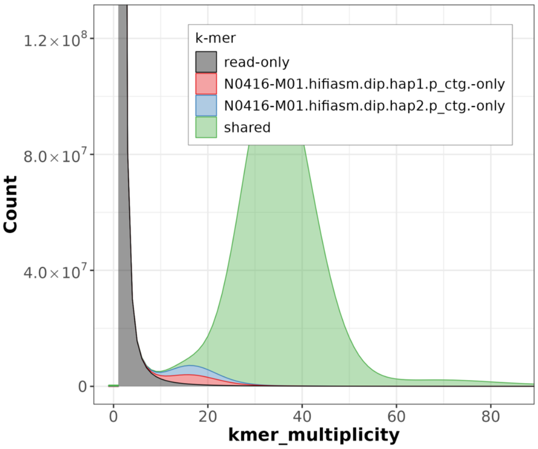

Jordanian

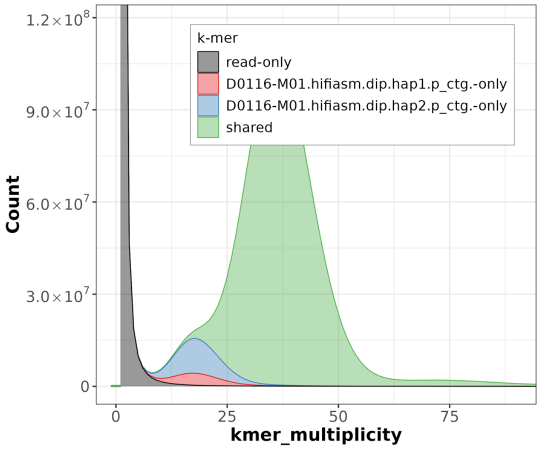

Syrian

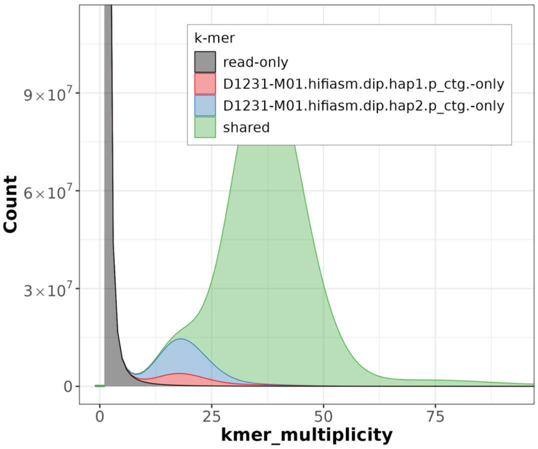

Qatari 1

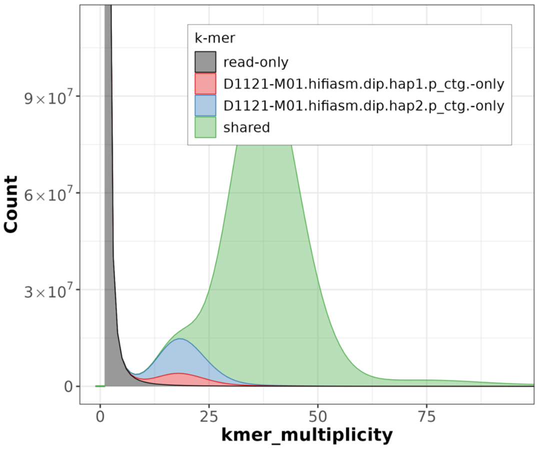

Qatari 2

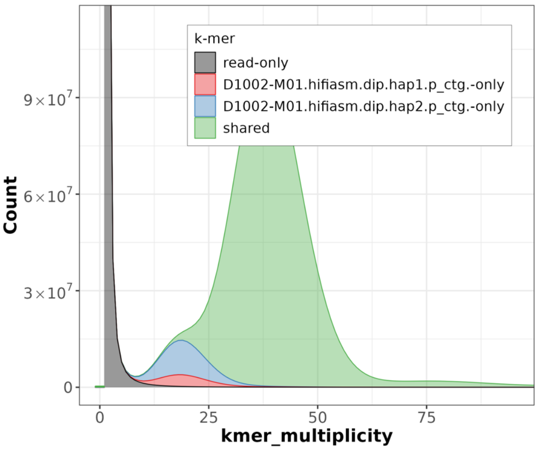

Sudanese

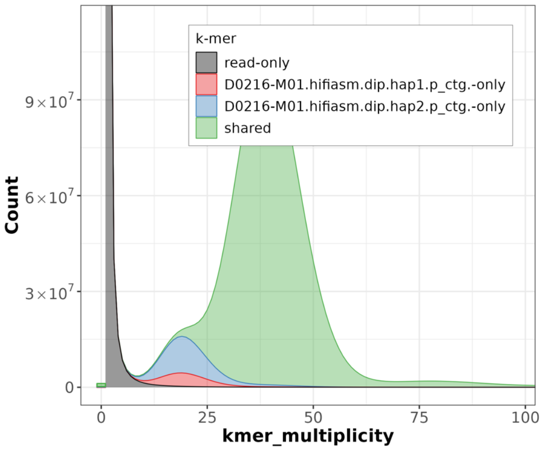

Supplementary Fig. 8: Number of mapped locations for contigs with alignment coverage >=0.9.

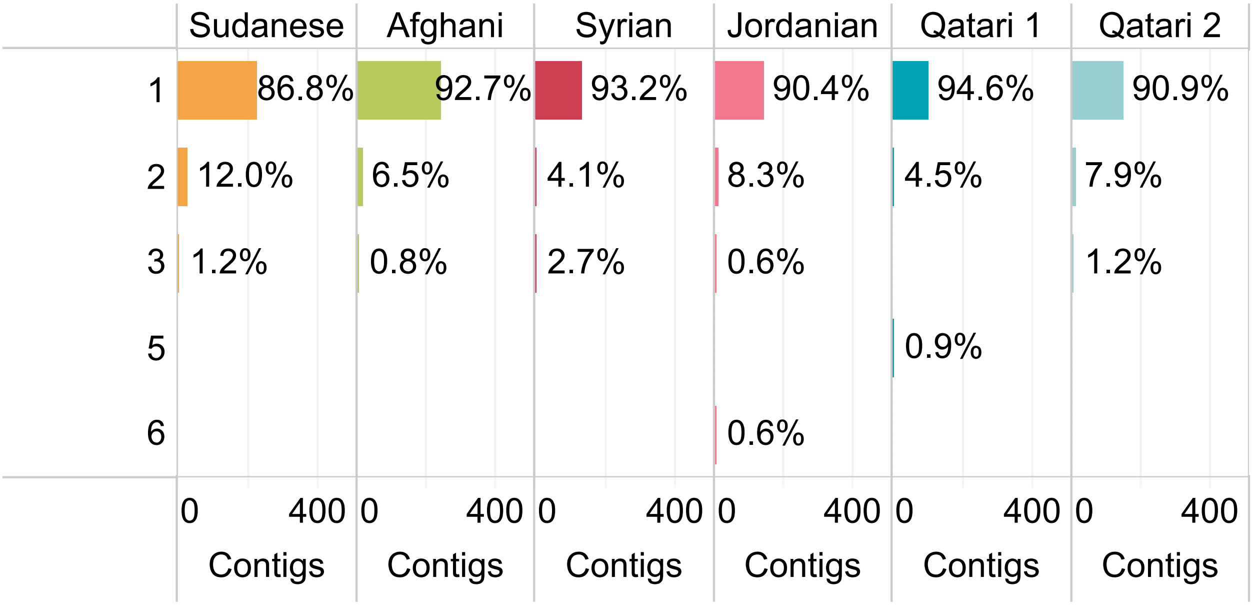

Supplementary Fig. 9: Other chromosome haplotypes spanned by a single contig in the child assemblies. Aligned to CHM13 and visualized on SafFire.

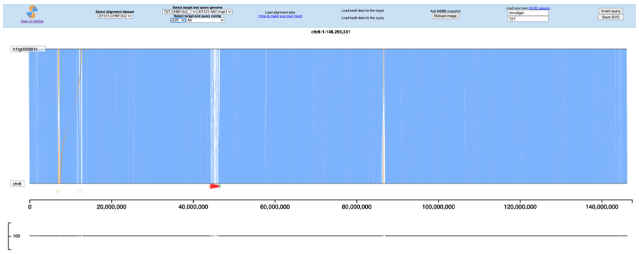

Qatari 1 hap1 :chr8

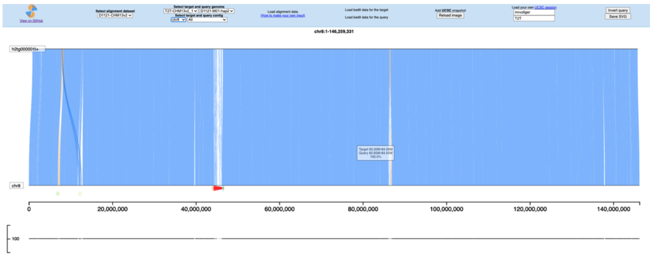

Qatari1 hap2 :chr8

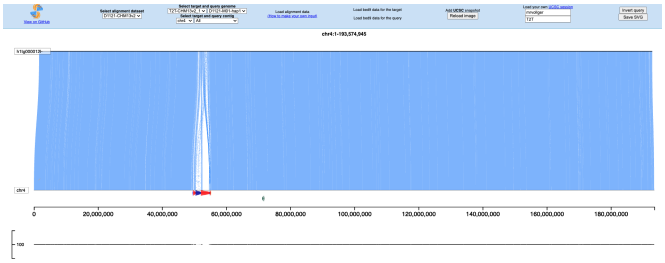

Qatari1 hap1 :chr4

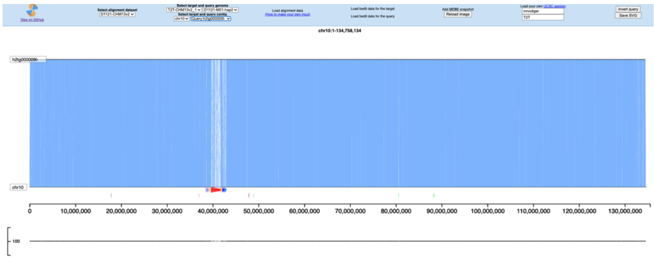

Qatari 1 hap2 :chr10

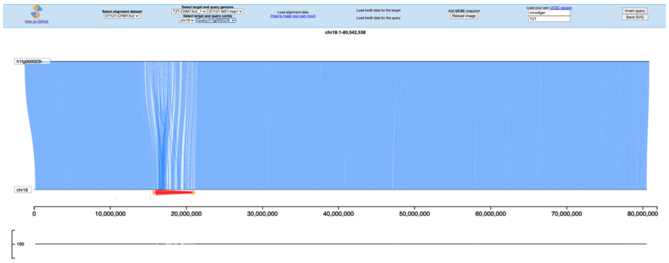

Qatari 1 hap1 :chr18

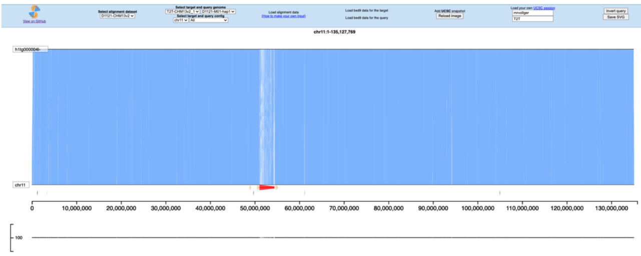

Qatari 1 hap1 :chr11

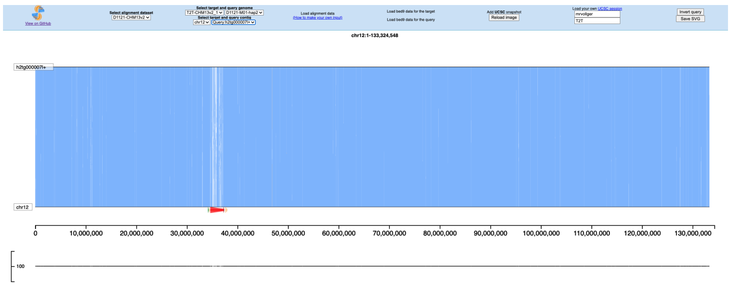

Qatari 1 hap2 :chr12

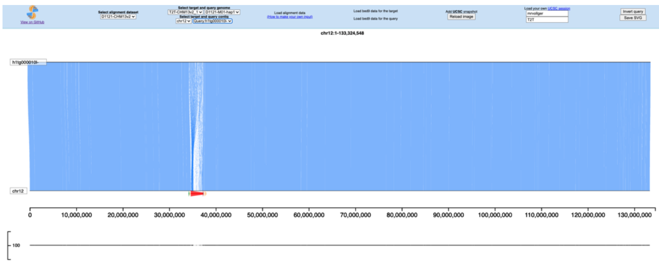

Qatari 1 hap1 :chr12

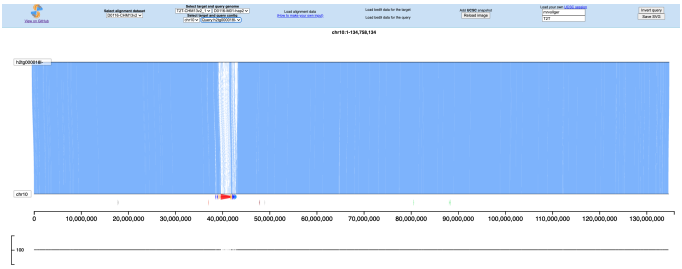

Jordanian hap2 :chr10

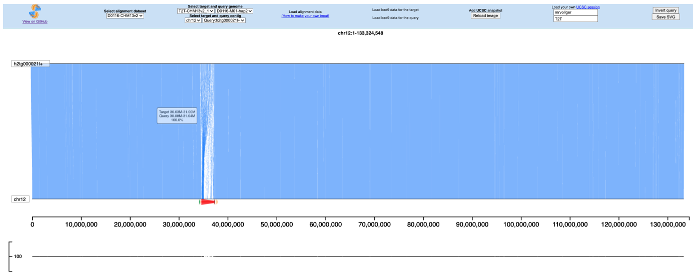

Jordanian hap 2 :chr12

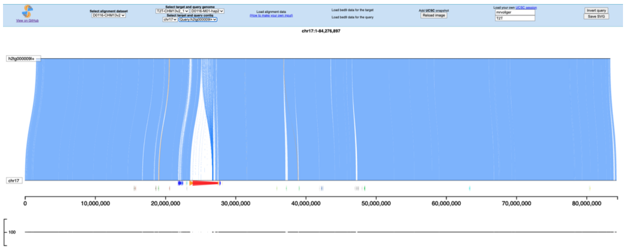

Jordanian hap2 :chr17

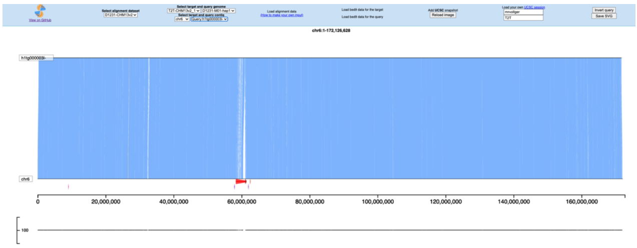

Syrian hap1 :chr6

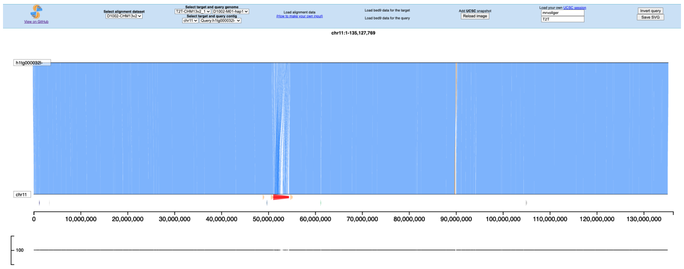

Qatar 2 hap1 :chr11

Supplementary Fig. 10: Novel sequences occurrence in various genetic features, as a function of comparison to other T2T genomes and patterns across chromosomes. (a) Total length of novel sequences in intergenic, intonic/UTR and exonic regions **(b)** Total length of novel sequences in segmental duplications (left) and stratified by occurrence in intergenic, intonic/UTR and exonic regions (right) **(c)** Novel sequences identified against CHM13 and HPRC upon incremental comparison with HG002 and CN1. **(d)** Length of novel sequences per chromosome. Dot size indicates the cumulative sequence length, while colors denote distinct assemblies. **(e)** Spread across the chromosomes highlighting the centromeric regions.

a

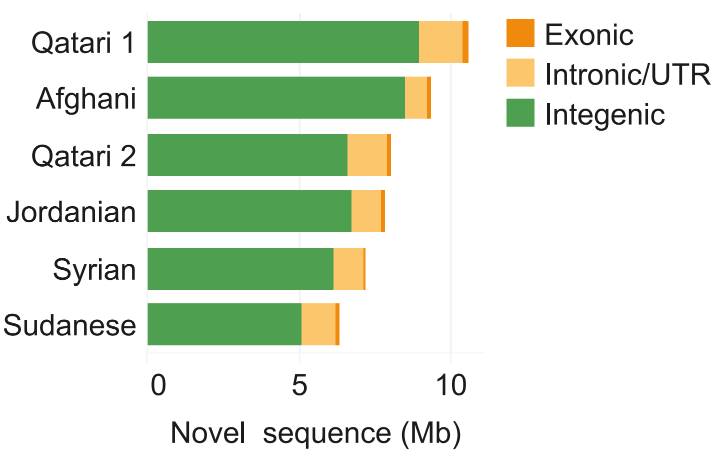

b

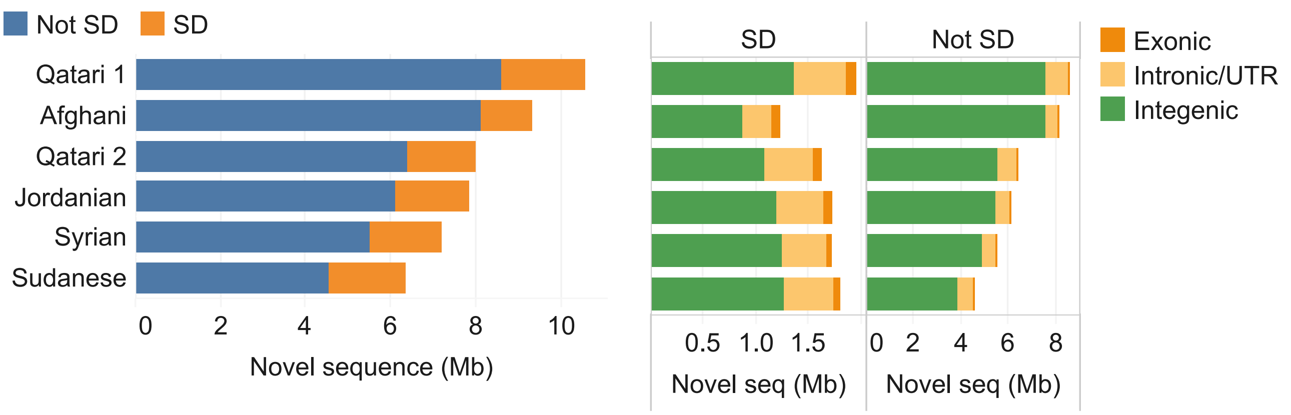

c

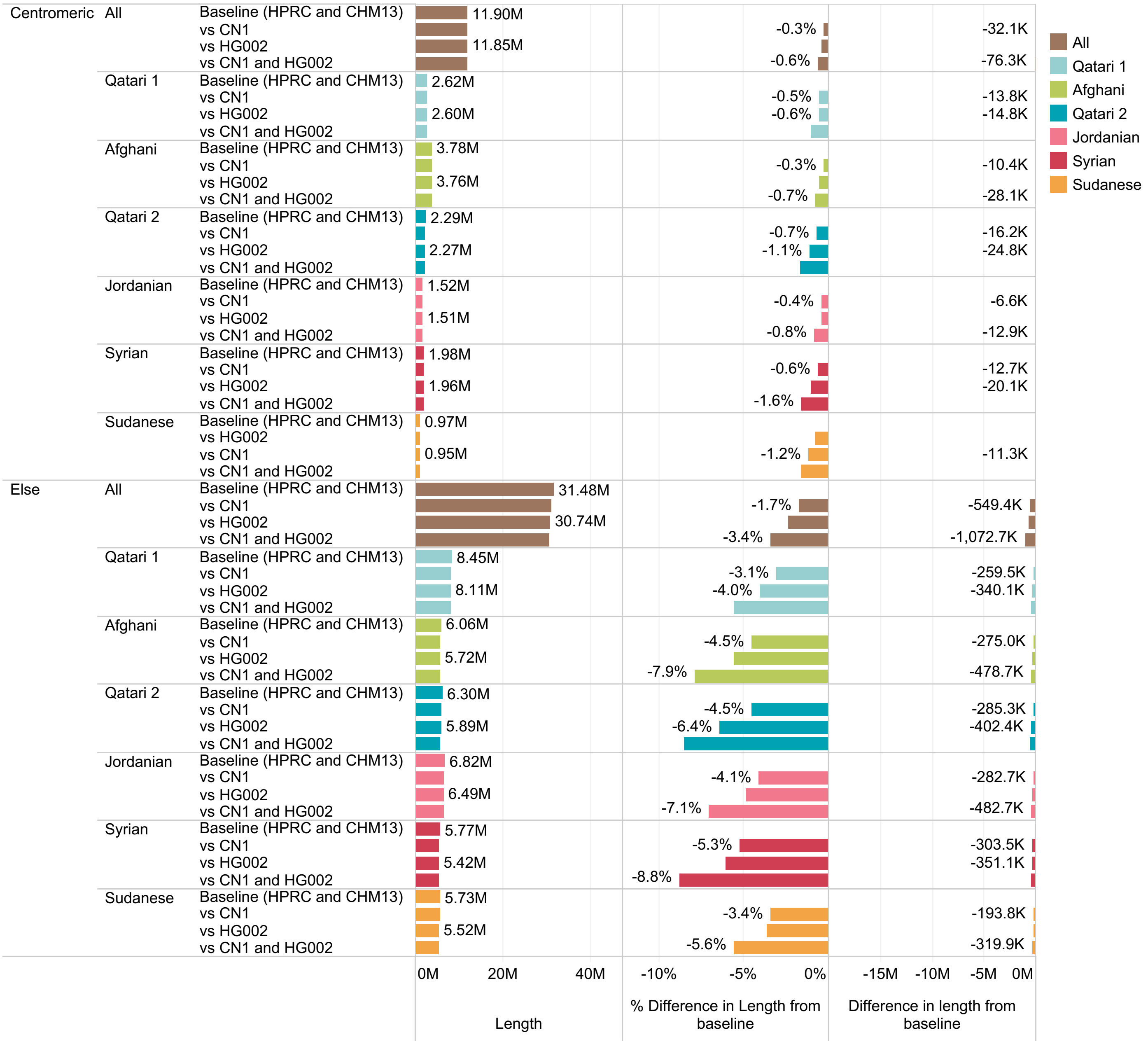

Centromeric
All
Baseline (HPRC and CHM13)
vs CN1
vs HG002
vs CN1 and HG002
11.90M
11.85M
-0.3%
-0.6%
-32.1K
-76.3K
Qatari 1
2.62M
2.60M
-0.5%
-0.6%
-13.8K
-14.8K
Afghani
3.78M
3.76M
-0.3%
-0.7%
-10.4K
-28.1K
Qatari 2
2.29M
2.27M
-0.7%
-1.1%
-16.2K
-24.8K
Jordanian
1.52M
1.51M
-0.4%
-0.8%
-6.6K
-12.9K
Syrian
1.98M
1.96M
-0.6%
-1.6%
-12.7K
-20.1K
Sudanese
0.97M
0.95M
-1.2%
-11.3K
Else
All
31.48M
30.74M
-1.7%
-3.4%
-549.4K
-1,072.7K
Qatari 1
8.45M
8.11M
-3.1%
-4.0%
-259.5K
-340.1K
Afghani
6.06M
5.72M
-4.5%
-7.9%
-275.0K
-478.7K
Qatari 2
6.30M
5.89M
-4.5%
-6.4%
-285.3K
-402.4K
Jordanian
6.82M
6.49M
-4.1%
-7.1%
-282.7K
-482.7K
Syrian
5.77M
5.42M
-5.3%
-8.8%
-303.5K
-351.1K
Sudanese
5.73M
5.52M
-3.4%
-5.6%
-193.8K
-319.9K
0M
20M
40M
Length
-10%
-5%
0%
% Difference in Length from baseline
-15M
-10M
-5M
0M
Difference in length from baseline
All
Qatari 1
Afghani
Qatari 2
Jordanian
Syrian
Sudanese

d

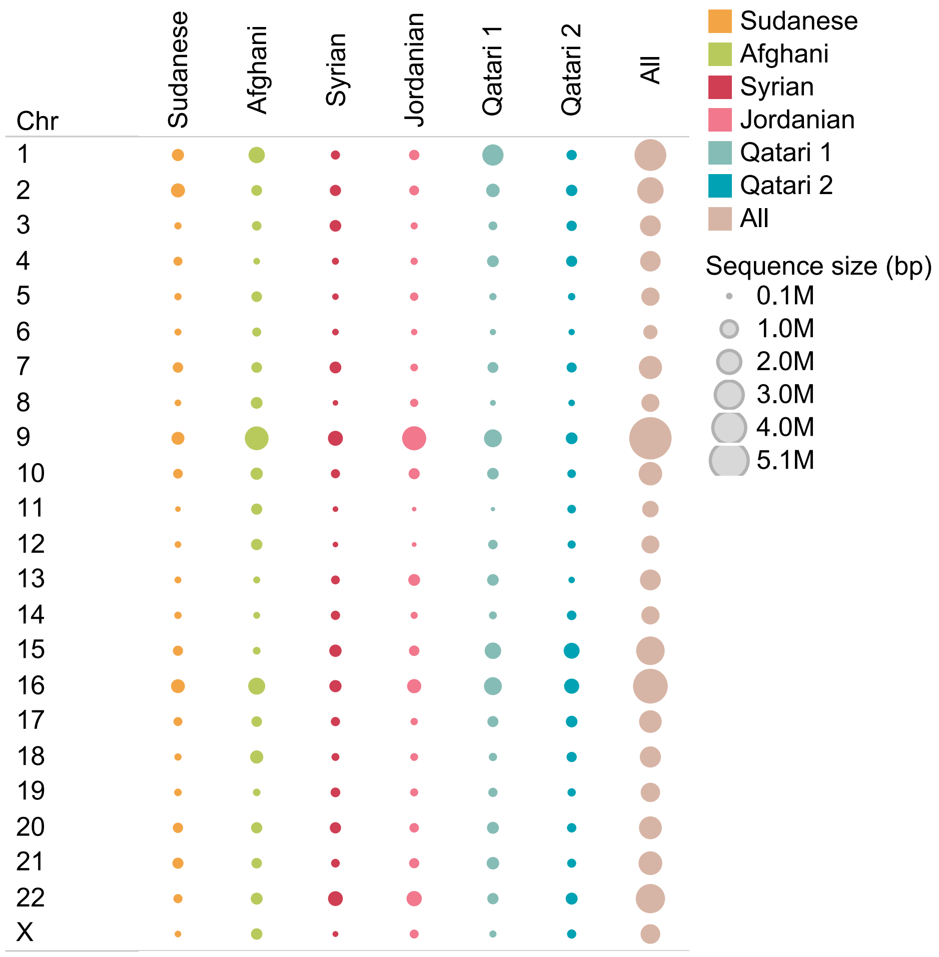
Chr
Sudanese
Afghani
Syrian
Jordanian
Qatari 1
Qatari 2
All
1
2
3
4
5
6
7
8
9
10
11
12
13
14
15
16
17
18
19
20
21
22
X
Sudanese
Afghani
Syrian
Jordanian
Qatari 1
Qatari 2
All
Sequence size (bp)
0.1M
1.0M
2.0M
3.0M
4.0M
5.1M

e

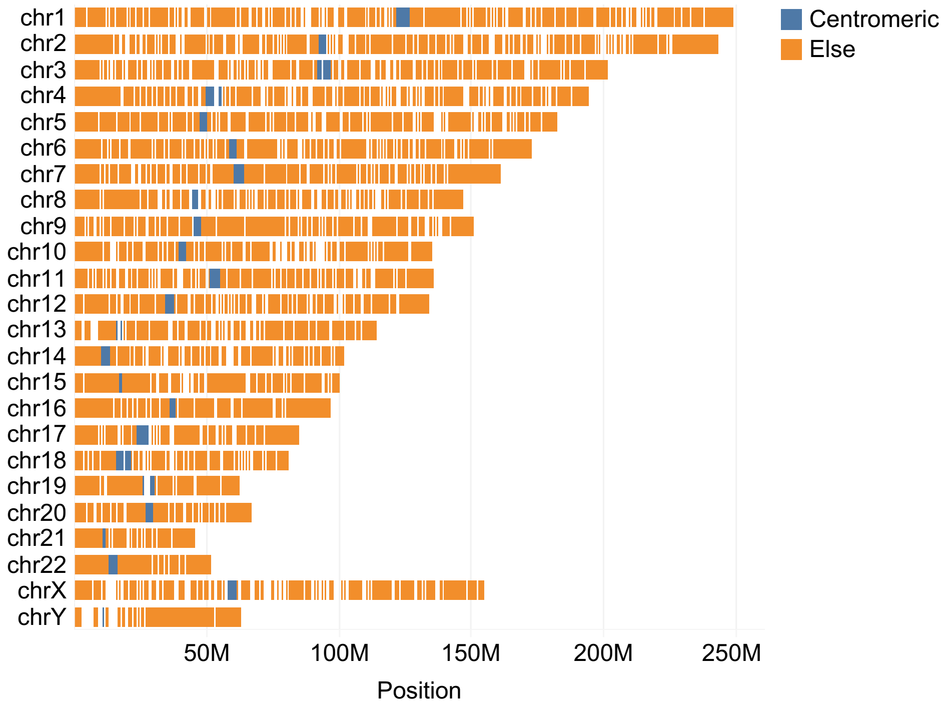
chr1
chr2
chr3
chr4
chr5
chr6
chr7
chr8
chr9
chr10
chr11
chr12
chr13
chr14
chr15
chr16
chr17
chr18
chr19
chr20
chr21
chr22
chrX
chrY
50M
100M
150M
200M
250M
Position
Centromeric
Else

Supplementary Fig. 11: Gene coverage of child assemblies relative to CHM13. (a) Number of genes annotated per chromosome haplotype for the child assemblies and CHM13. **(b and c)** Coverage of annotated genes for the six largest gene categories in chromosome X and Y respectively.

a

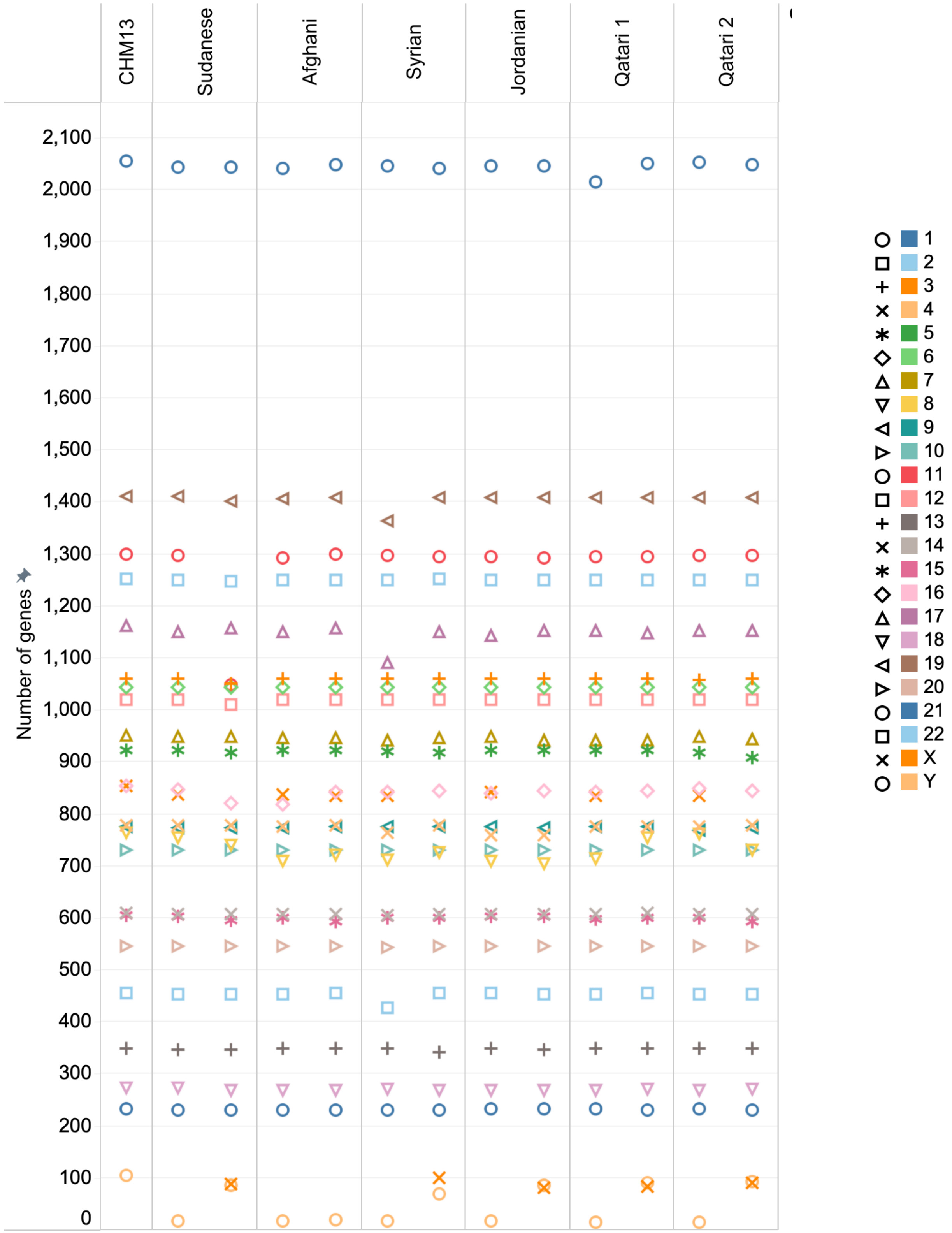

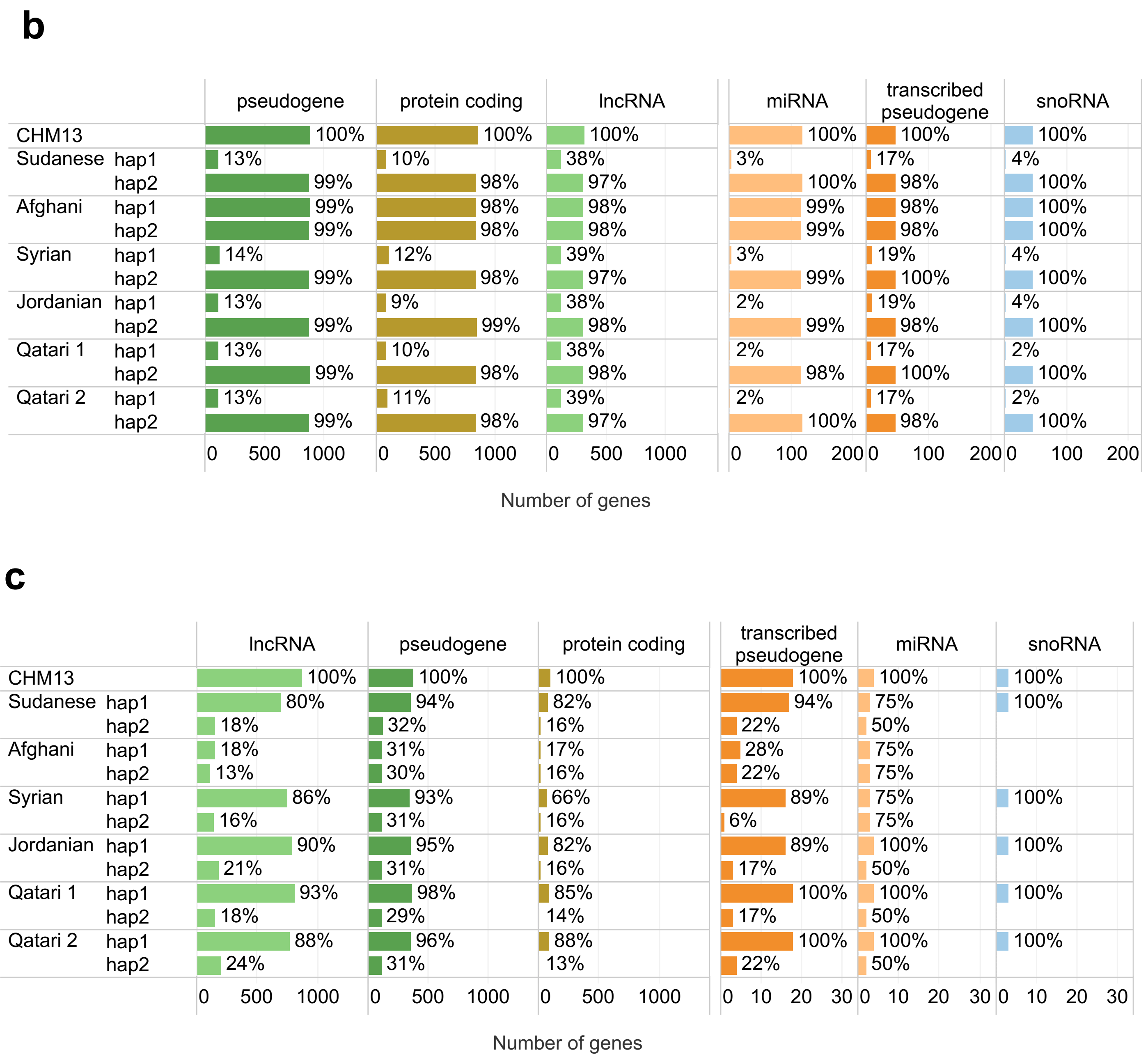
b
pseudogene
protein coding
lncRNA
miRNA
transcribed pseudogene
snoRNA
CHM13 100% 100% 100% 100% 100% 100%
Sudanese hap1 13% 10% 38% 3% 17% 4%
hap2 99% 98% 97% 100% 98% 100%
Afghani hap1 99% 98% 98% 99% 98% 100%
hap2 99% 98% 98% 99% 98% 100%
Syrian hap1 14% 12% 39% 3% 19% 4%
hap2 99% 98% 97% 99% 100% 100%
Jordanian hap1 13% 9% 38% 2% 19% 4%
hap2 99% 99% 98% 99% 98% 100%
Qatari 1 hap1 13% 10% 38% 2% 17% 2%
hap2 99% 98% 98% 98% 100% 100%
Qatari 2 hap1 13% 11% 39% 2% 17% 2%
hap2 99% 98% 97% 100% 98% 100%
0 500 1000
0 100 200
Number of genes
c
lncRNA
pseudogene
protein coding
transcribed pseudogene
miRNA
snoRNA
CHM13 100% 100% 100% 100% 100% 100%
Sudanese hap1 80% 94% 82% 94% 75% 100%
hap2 18% 32% 16% 22% 50%
Afghani hap1 18% 31% 17% 28% 75%
hap2 13% 30% 16% 22% 75%
Syrian hap1 86% 93% 66% 89% 75% 100%
hap2 16% 31% 16% 6% 75%
Jordanian hap1 90% 95% 82% 89% 100% 100%
hap2 21% 31% 16% 17% 50%
Qatari 1 hap1 93% 98% 85% 100% 100% 100%
hap2 18% 29% 14% 17% 50%
Qatari 2 hap1 88% 96% 88% 100% 100% 100%
hap2 24% 31% 13% 22% 50%
0 500 1000
0 10 20 30
Number of genes

Supplementary Fig. 12: *HLA* and *KIR* gene copies. Shown are annotated *HLA* and *KIR* genes across ME child assemblies, benchmarked against 220 reference haplotypes. Each dot denotes the presence of gene copies, with colors indicating the number of copies detected.

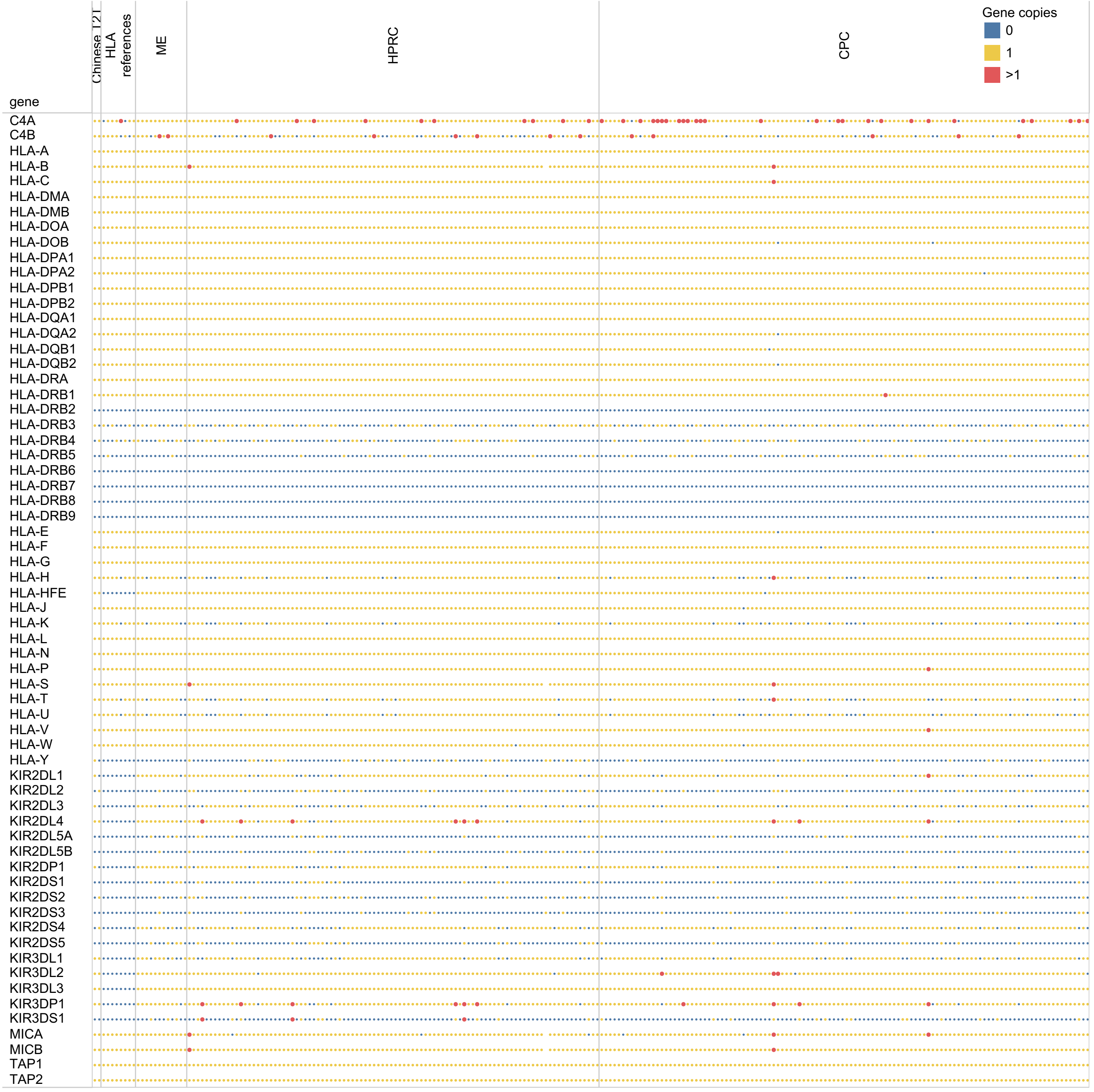

Supplementary Fig. 13. Allele frequencies of *HLA-A* alleles in the child assemblies. For each child subject, log allele frequency of each haplotype *HLA-A* allele is shown across various world populations. Alleles are categorized in each population by the CIWD catalogue v 3.0 as *C*, *I*, *WD* and *NA* denoting common, intermediate, well-documented and not applicable, respectively. The populations are defined by CIWD and denoted as follows: AFA (African/African American), API (Asian/Pacific Islands), EUR (European/European descent), ME (Middle East/North Coast of Africa), HIS (South or Central America/Hispanic/Latino), NAM (Native American populations) and UNK (unknown/not asked/multiple ancestries/other). Total is the overall population i.e., all groups combined.

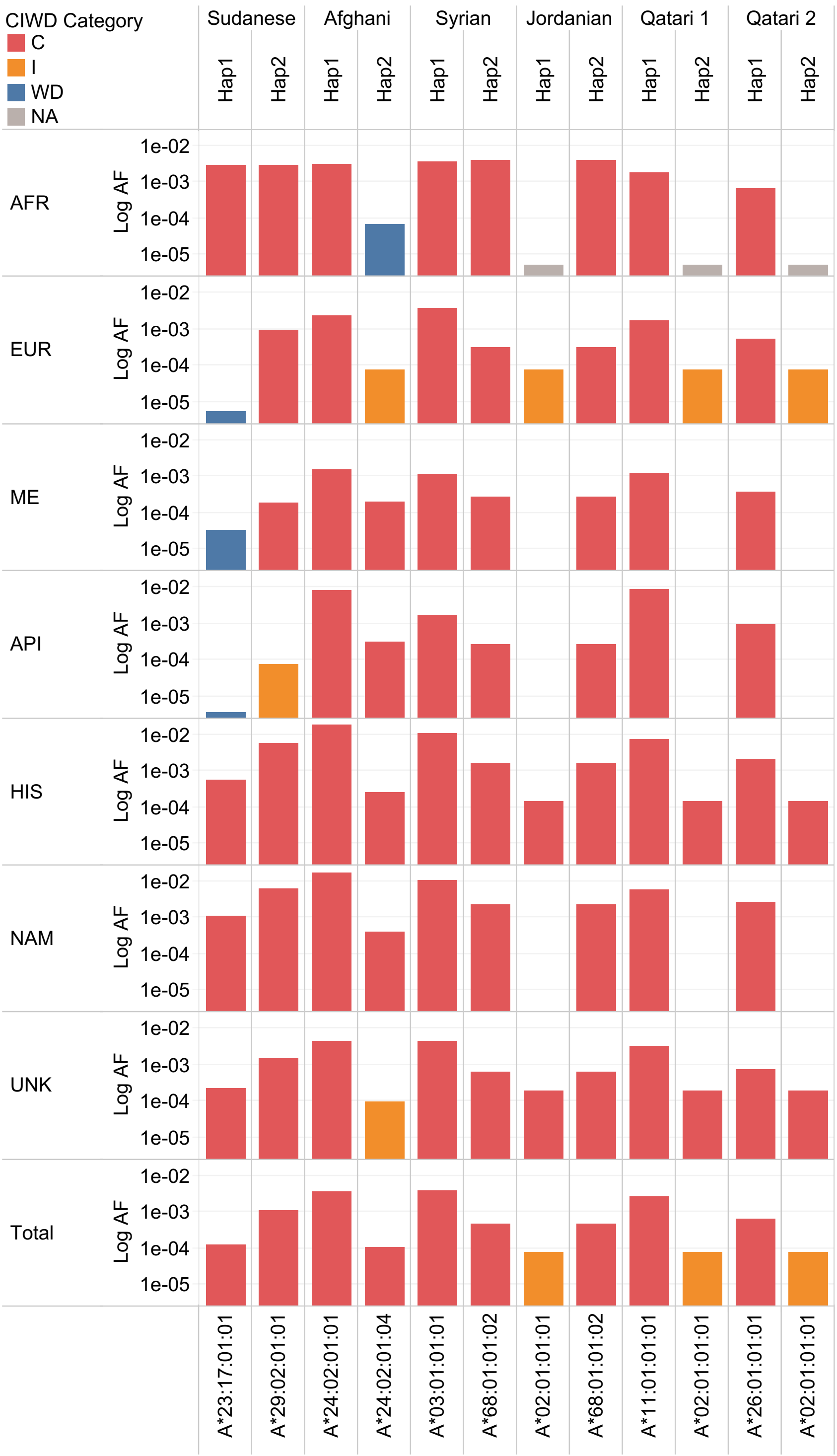

Supplementary Fig. 14. Novel *HLA* and *KIR* alleles with mutations in the CDS in the child assemblies. (a) Novel alleles are shown together with the corresponding genes and haplotypes for each subject **(b)** Per allele and subject, the number of known alleles with the shortest CDS distance and the number of CDS mutations that are either missense novel, missense known or synonymous. **(c)** Details of the novel alleles including the DNA alterations relative to known alleles with the closest CDS, as well as the population ancestry of the source samples in IPD for those related alleles.

a

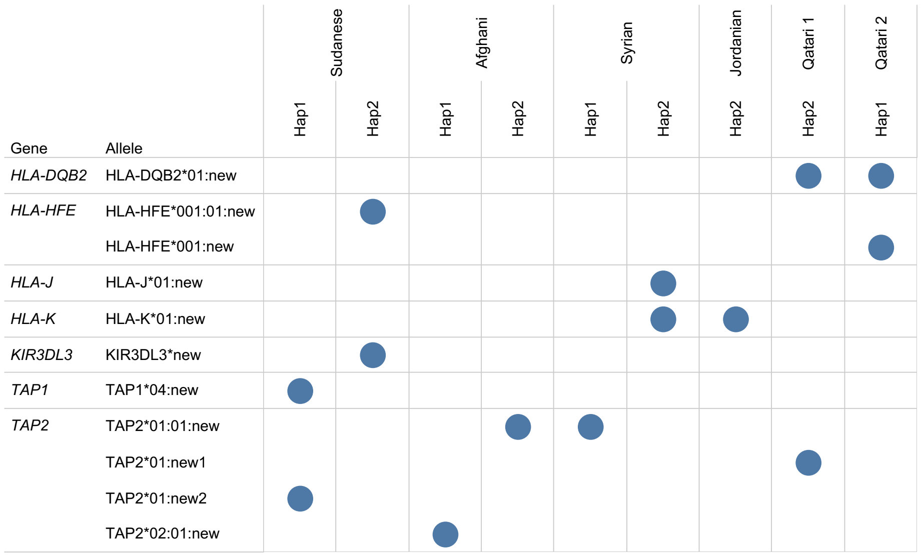

b

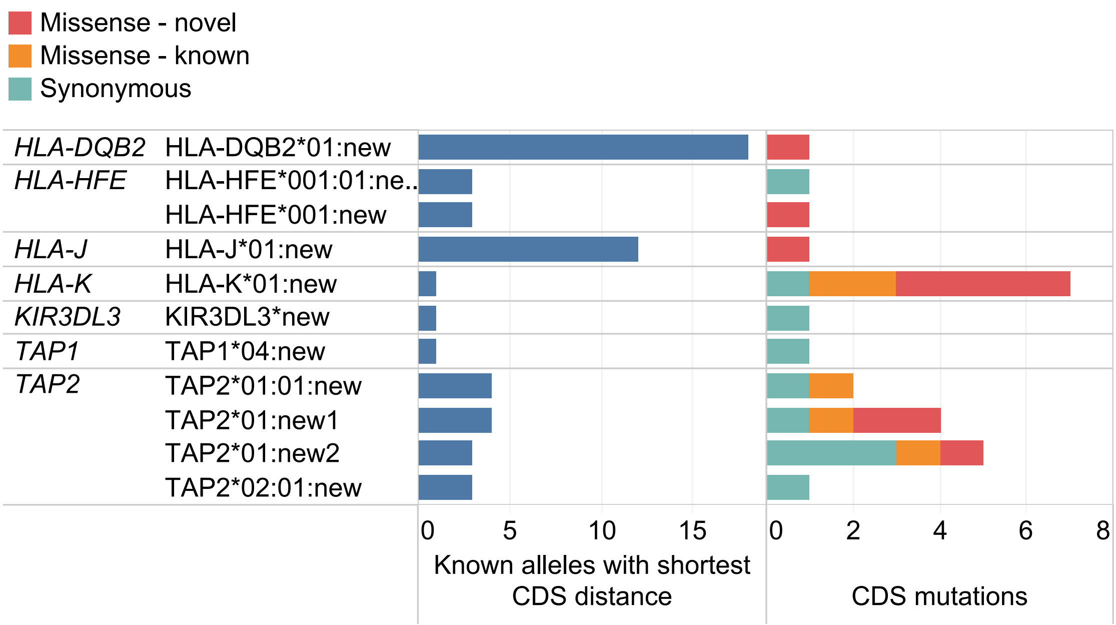

c

Ancestry of source samples in IPD for related alleles
- Sub-Sahran African
- East Asian
- European
- Null
- Native American

Allele	CDS Distance	Mutation	Novel DNA mutation	Alteration	Category	Related Allele	
HLA-DQB2*01:new	1	:7_ag:799	Yes	W(TGG)<_(TAG)	Missense - novel	HLA-DQB2*01:01:01:01	European, East Asian
						HLA-DQB2*01:01:01:02	European
						HLA-DQB2*01:01:01:03	European
						HLA-DQB2*01:01:01:04	European
						HLA-DQB2*01:01:01:05	Null
						HLA-DQB2*01:01:01:06	Null
						HLA-DQB2*01:01:01:07	East Asian
						HLA-DQB2*01:01:01:08	European
						HLA-DQB2*01:01:01:09	European
						HLA-DQB2*01:01:01:10	Sub-Sahran African
						HLA-DQB2*01:01:01:11	European
						HLA-DQB2*01:01:01:12	Null
						HLA-DQB2*01:01:01:13	European
						HLA-DQB2*01:01:01:14	Null
						HLA-DQB2*01:01:01:15	East Asian
						HLA-DQB2*01:01:01:16	Null
						HLA-DQB2*01:01:01:17	Null
						HLA-DQB2*01:01:01:18	Null
HLA-HFE*001:01:new	1	:1025_tc:21	Yes	Y(TAC)<Y(TAT)	Synonymous	HLA-HFE*001:01:01	European
						HLA-HFE*001:01:02	Null
						HLA-HFE*001:01:03	Null
HLA-HFE*001:new	1	:828_ag:218	Yes	E(GAG)<K(AAG)	Missense - novel	HLA-HFE*001:01:01	European
						HLA-HFE*001:01:02	Null
						HLA-HFE*001:01:03	Null
HLA-J*01:new	1	:931_ct:163	Yes	I(ATT)<T(ACT)	Missense - novel	HLA-J*01:01:01:01	European
						HLA-J*01:01:01:02	European
						HLA-J*01:01:01:03	Null
						HLA-J*01:01:01:04	European
						HLA-J*01:01:01:05	European, Native American
						HLA-J*01:01:01:11	European
						HLA-J*01:01:01:14	European
						HLA-J*01:01:01:15	European
						HLA-J*01:01:01:17	European
						HLA-J*01:01:01:27	East Asian
						HLA-J*01:01:01:28	European
						HLA-J*01:01:01:30	Null
HLA-K*01:new	7	:7_ct:123	No	L(TTA)<S(TCA)	Missense - known	HLA-K*01:02	European
		ag:8	No	N(AAC)<K(AAG)	Missense - known	HLA-K*01:02	European
		gc:238	No	N(AAC)<K(AAG)	Missense - novel	HLA-K*01:02	European
		gc:238-cg:465	Yes	R(CGC)<CCGGC(CCGGC)	Missense - novel	HLA-K*01:02	European
		gc:246	Yes	P(CCC)<P(CCG)	Synonymous	HLA-K*01:02	European
		tc:1	No	R(CGG)<_(TGA)	Missense - novel	HLA-K*01:02	European
		tc:1, ag:8	Yes	R(CGG)<_(TGA)	Missense - novel	HLA-K*01:02	European
KIR3DL3*new	1	:167_ct:1065	No	N(AAT)<N(AAC)	Synonymous	KIR3DL3*035	Null
TAP1*04:new	1	:158_ga:2088	Yes	P(CCA)<P(CCG)	Synonymous	TAP1*04:01	Null
TAP2*01:01:new	1	:1157_tg:903	No	G(GGG)<G(GGT)	Synonymous	TAP2*01:01:03:01	Null
						TAP2*01:01:03:02	Null
						TAP2*01:01:03:03	Null
		:1692_ga:368	No	T(ACT)<A(GCT)	Missense - known	TAP2*01:02	Null
TAP2*01:new1	2	:1157_tg:258	No	G(GGG)<G(GGT)	Synonymous	TAP2*01:01:03:01	Null
						TAP2*01:01:03:02	Null
						TAP2*01:01:03:03	Null
		:1416_ag:275	Yes	V(GTC)<I(ATC)	Missense - novel	TAP2*01:02	Null
		ag:644	Yes	V(GTC)<I(ATC)	Missense - novel	TAP2*01:01:03:01	Null
						TAP2*01:01:03:02	Null
						TAP2*01:01:03:03	Null
		ga:368	No	T(ACT)<A(GCT)	Missense - known	TAP2*01:02	Null
TAP2*01:new2	5	:657_ac:461	Yes	R(CGA)<R(AGA)	Synonymous	TAP2*01:01:03:01	Null
						TAP2*01:01:03:02	Null
						TAP2*01:01:03:03	Null
		ag	Yes	G(GGG)<G(GGA)	Synonymous	TAP2*01:01:03:01	Null
						TAP2*01:01:03:02	Null
						TAP2*01:01:03:03	Null
		ag:23	Yes	T(ACG)<T(ACA)	Synonymous	TAP2*01:01:03:01	Null
						TAP2*01:01:03:02	Null
						TAP2*01:01:03:03	Null
		ag:253	Yes	A(GCC)<T(ACC)	Missense - novel	TAP2*01:01:03:01	Null
						TAP2*01:01:03:02	Null
						TAP2*01:01:03:03	Null
		ag:662	Yes	V(GTT)<I(ATT)	Missense - known	TAP2*01:01:03:01	Null
						TAP2*01:01:03:02	Null
						TAP2*01:01:03:03	Null
TAP2*02:01:new	1	:1157_tg:954	No	G(GGG)<G(GGT)	Synonymous	TAP2*02:01:02:01	Null
						TAP2*02:01:02:02	Null
						TAP2*02:01:02:03	Null

Supplementary Fig. 15. Alignments of the Coding DNA sequences (CDS) and corresponding amino acid sequences for the novel alleles in the child subjects against reference sequences from IPD. For each locus, CDS alignment is colored by position-level percentage identity and the amino acid alignment is colored by residue type. Consensus plot is indicated at the bottom. The novel allele names are highlighted in grey, and their sequence are contoured with a discontinued red line. At the top of the alignments, DNA mutations are annotated with *N* (Novel) or *n* (known) while amino acid mutations are labeled as *M* (Missense) or *s* (synonymous).

HLA-DQB2

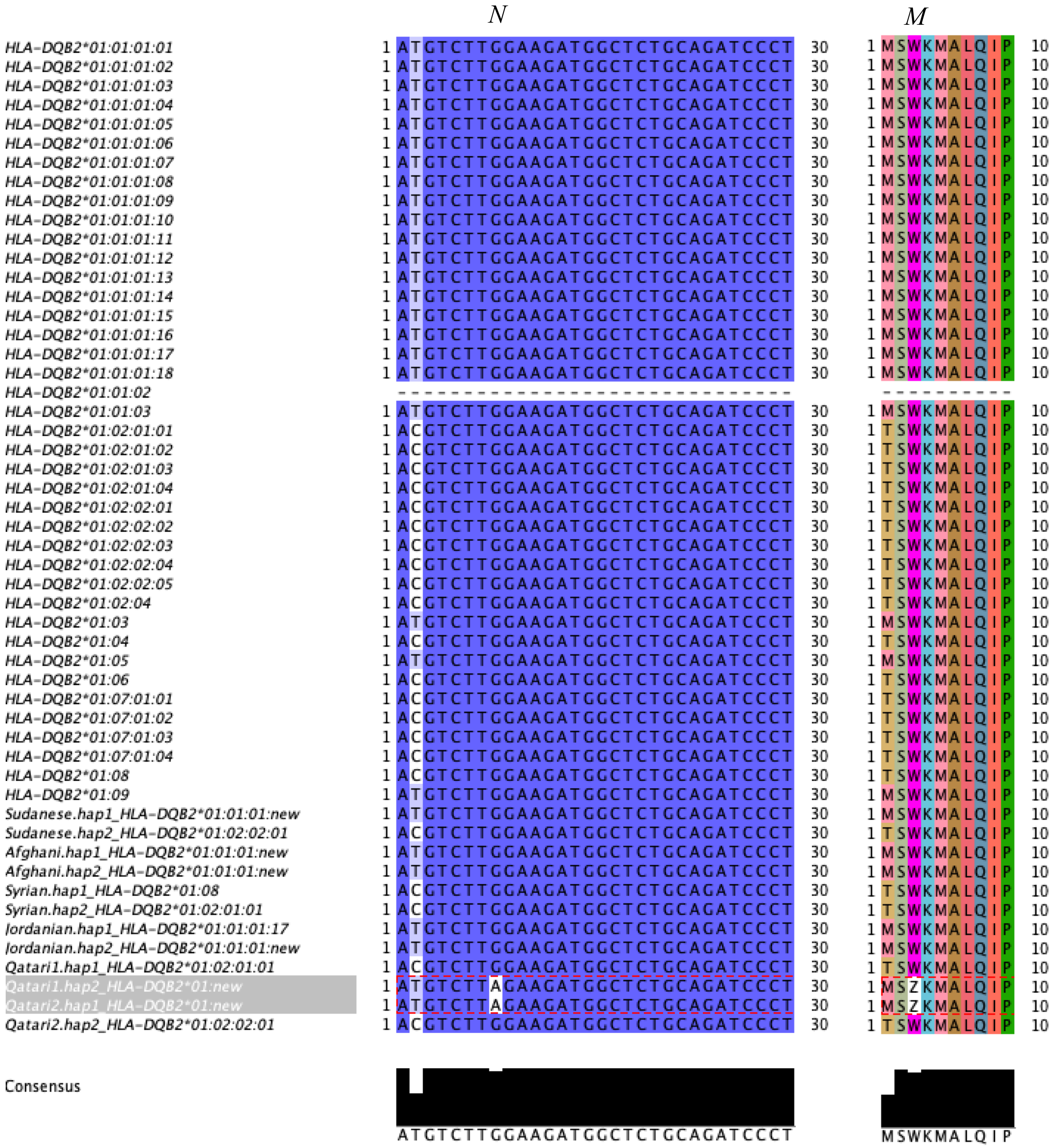

HLA-HFE

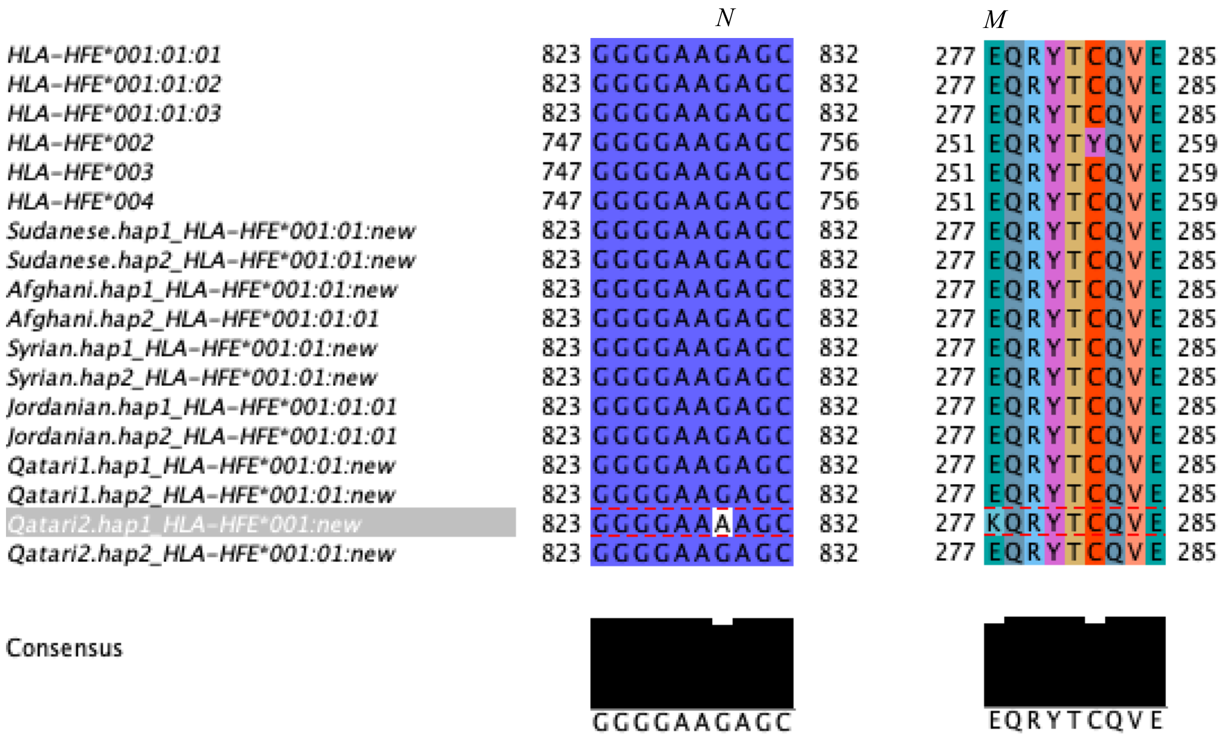
N
M
HLA-HFE*001:01:01 823 GGGGAAGAGC 832 277 EQRYTCQVE 285
HLA-HFE*001:01:02 823 GGGGAAGAGC 832 277 EQRYTCQVE 285
HLA-HFE*001:01:03 823 GGGGAAGAGC 832 277 EQRYTCQVE 285
HLA-HFE*002 747 GGGGAAGAGC 756 251 EQRYTYQVE 259
HLA-HFE*003 747 GGGGAAGAGC 756 251 EQRYTCQVE 259
HLA-HFE*004 747 GGGGAAGAGC 756 251 EQRYTCQVE 259
Sudanese.hap1_HLA-HFE*001:01:new 823 GGGGAAGAGC 832 277 EQRYTCQVE 285
Sudanese.hap2_HLA-HFE*001:01:new 823 GGGGAAGAGC 832 277 EQRYTCQVE 285
Afghani.hap1_HLA-HFE*001:01:new 823 GGGGAAGAGC 832 277 EQRYTCQVE 285
Afghani.hap2_HLA-HFE*001:01:01 823 GGGGAAGAGC 832 277 EQRYTCQVE 285
Syrian.hap1_HLA-HFE*001:01:new 823 GGGGAAGAGC 832 277 EQRYTCQVE 285
Syrian.hap2_HLA-HFE*001:01:new 823 GGGGAAGAGC 832 277 EQRYTCQVE 285
Jordanian.hap1_HLA-HFE*001:01:01 823 GGGGAAGAGC 832 277 EQRYTCQVE 285
Jordanian.hap2_HLA-HFE*001:01:01 823 GGGGAAGAGC 832 277 EQRYTCQVE 285
Qatari1.hap1_HLA-HFE*001:01:new 823 GGGGAAGAGC 832 277 EQRYTCQVE 285
Qatari1.hap2_HLA-HFE*001:01:new 823 GGGGAAGAGC 832 277 EQRYTCQVE 285
Qatari2.hap1_HLA-HFE*001:new 823 GGGGAAAAGC 832 277 KQRYTCQVE 285
Qatari2.hap2_HLA-HFE*001:01:new 823 GGGGAAGAGC 832 277 EQRYTCQVE 285
Consensus
GGGGAAGAGC
EQRYTCQVE

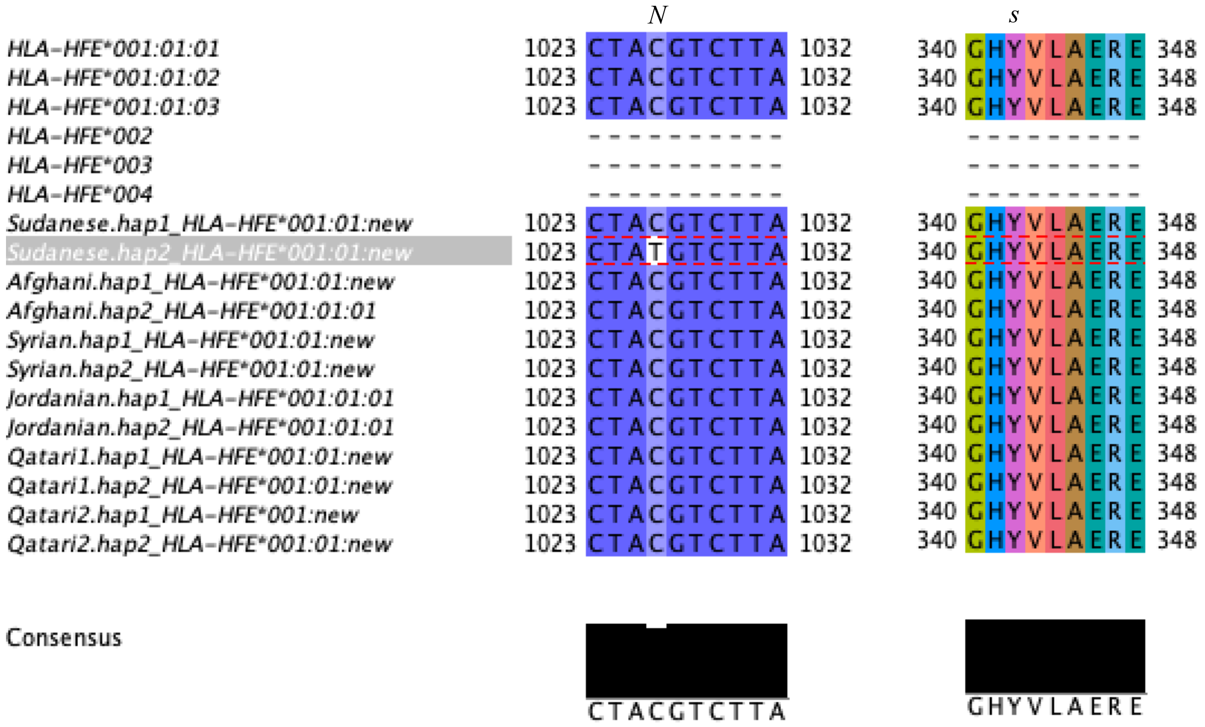
N
s
HLA-HFE*001:01:01 1023 CTACGTCTTA 1032 340 GHYVLAERE 348
HLA-HFE*001:01:02 1023 CTACGTCTTA 1032 340 GHYVLAERE 348
HLA-HFE*001:01:03 1023 CTACGTCTTA 1032 340 GHYVLAERE 348
HLA-HFE*002 ---------- ---------
HLA-HFE*003 ---------- ---------
HLA-HFE*004 ---------- ---------
Sudanese.hap1_HLA-HFE*001:01:new 1023 CTACGTCTTA 1032 340 GHYVLAERE 348
Sudanese.hap2_HLA-HFE*001:01:new 1023 CTATGTCTTA 1032 340 GHYVLAERE 348
Afghani.hap1_HLA-HFE*001:01:new 1023 CTACGTCTTA 1032 340 GHYVLAERE 348
Afghani.hap2_HLA-HFE*001:01:01 1023 CTACGTCTTA 1032 340 GHYVLAERE 348
Syrian.hap1_HLA-HFE*001:01:new 1023 CTACGTCTTA 1032 340 GHYVLAERE 348
Syrian.hap2_HLA-HFE*001:01:new 1023 CTACGTCTTA 1032 340 GHYVLAERE 348
Jordanian.hap1_HLA-HFE*001:01:01 1023 CTACGTCTTA 1032 340 GHYVLAERE 348
Jordanian.hap2_HLA-HFE*001:01:01 1023 CTACGTCTTA 1032 340 GHYVLAERE 348
Qatari1.hap1_HLA-HFE*001:01:new 1023 CTACGTCTTA 1032 340 GHYVLAERE 348
Qatari1.hap2_HLA-HFE*001:01:new 1023 CTACGTCTTA 1032 340 GHYVLAERE 348
Qatari2.hap1_HLA-HFE*001:new 1023 CTACGTCTTA 1032 340 GHYVLAERE 348
Qatari2.hap2_HLA-HFE*001:01:new 1023 CTACGTCTTA 1032 340 GHYVLAERE 348
Consensus
CTACGTCTTA
GHYVLAERE

HLA-J

Allele	Start	*N*	End	Start	*M*	End
HLA-J*01:01:01:01	927	TATCATTGCT	936	308	VGIIAGLVLL	317
HLA-J*01:01:01:02	927	TATCATTGCT	936	308	VGIIAGLVLL	317
HLA-J*01:01:01:03	927	TATCATTGCT	936	308	VGIIAGLVLL	317
HLA-J*01:01:01:04	927	TATCATTGCT	936	308	VGIIAGLVLL	317
HLA-J*01:01:01:05	927	TATCATTGCT	936	308	VGIIAGLVLL	317
HLA-J*01:01:01:06	927	TATCATTGCT	936	308	VGIIAGLVLL	317
HLA-J*01:01:01:07	929	TATCATTGCT	938	310	VSLLAWF---	316
HLA-J*01:01:01:08	928	TATCATTGCT	937	308	CGYHC-W---	313
HLA-J*01:01:01:09	920	TATCATTGCT	929	307	VSLLAWF---	313
HLA-J*01:01:01:10	921	TATCATTGCT	930	306	VGIIAGLVLL	315
HLA-J*01:01:01:11	927	TATCATTGCT	936	308	VGIIAGLVLL	317
HLA-J*01:01:01:12	927	TATCATTGCT	936	308	VGIIAGLVLL	317
HLA-J*01:01:01:13	920	TATCATTGCT	929	307	VSLLAWF---	313
HLA-J*01:01:01:14	927	TATCATTGCT	936	308	VGIIAGLVLL	317
HLA-J*01:01:01:15	927	TATCATTGCT	936	308	VGIIAGLVLL	317
HLA-J*01:01:01:16	928	TATCATTGCT	937	308	CGYHC-W---	313
HLA-J*01:01:01:17	927	TATCATTGCT	936	308	VGIIAGLVLL	317
HLA-J*01:01:01:18	928	TATCATTGCT	937	308	CGYHC-W---	313
HLA-J*01:01:01:19	929	TATCATTGCT	938	310	VSLLAWF---	316
HLA-J*01:01:01:20	928	TATCATTGCT	937	308	CGYHC-W---	313
HLA-J*01:01:01:21	928	TATCATTGCT	937	308	CGYHC-W---	313
HLA-J*01:01:01:22	927	TATCATTGCT	936	308	VGIIAGLVLL	317
HLA-J*01:01:01:23	927	TATCATTGCT	936	308	VGIIAGLVLL	317
HLA-J*01:01:01:24	927	TATCATTGCT	936	308	VGIIAGLVLL	317
HLA-J*01:01:01:25	927	TATCATTGCT	936	308	VGIIAGLVLL	317
HLA-J*01:01:01:26	927	TATCATTGCT	936	308	VGIIAGLVLL	317
HLA-J*01:01:01:27	927	TATCATTGCT	936	308	VGIIAGLVLL	317
HLA-J*01:01:01:28	927	TATCATTGCT	936	308	VGIIAGLVLL	317
HLA-J*01:01:01:29	920	TATCATTGCT	929	307	VSLLAWF---	313
HLA-J*01:01:01:30	927	TATCATTGCT	936	308	VGIIAGLVLL	317
HLA-J*01:01:01:31	927	TATCATTGCT	936	308	VGIIAGLVLL	317
HLA-J*01:01:01:32	927	TATCATTGCT	936	308	VGIIAGLVLL	317
HLA-J*02:01	926	TATCATTGCT	935	309	VSLLAWF---	315
Sudanese.hap1_HLA-J*01:01:01:15	927	TATCATTGCT	936	308	VGIIAGLVLL	317
Sudanese.hap2_HLA-J*01:01:01:01	927	TATCATTGCT	936	308	VGIIAGLVLL	317
Afghani.hap1_HLA-J*01:01:01:02	927	TATCATTGCT	936	308	VGIIAGLVLL	317
Afghani.hap2_HLA-J*01:01:01:02	927	TATCATTGCT	936	308	VGIIAGLVLL	317
Syrian.hap1_HLA-J*01:01:01:04	927	TATCATTGCT	936	308	VGIIAGLVLL	317
Syrian.hap2_HLA-J*01:new	927	TATCACTGCT	936	308	VGITAGLVLL	317
Jordanian.hap1_HLA-J*01:01:01:05	927	TATCATTGCT	936	308	VGIIAGLVLL	317
Jordanian.hap2_HLA-J*01:01:01:01	927	TATCATTGCT	936	308	VGIIAGLVLL	317
Qatari1.hap1_HLA-J*01:01:01:04	927	TATCATTGCT	936	308	VGIIAGLVLL	317
Qatari1.hap2_HLA-J*01:01:01:17	927	TATCATTGCT	936	308	VGIIAGLVLL	317
Qatari2.hap1_HLA-J*01:01:01:01	927	TATCATTGCT	936	308	VGIIAGLVLL	317
Qatari2.hap2_HLA-J*01:01:01:17	927	TATCATTGCT	936	308	VGIIAGLVLL	317
Consensus		TATCATTGCT			VGIIAGLVLL	

HLA-K

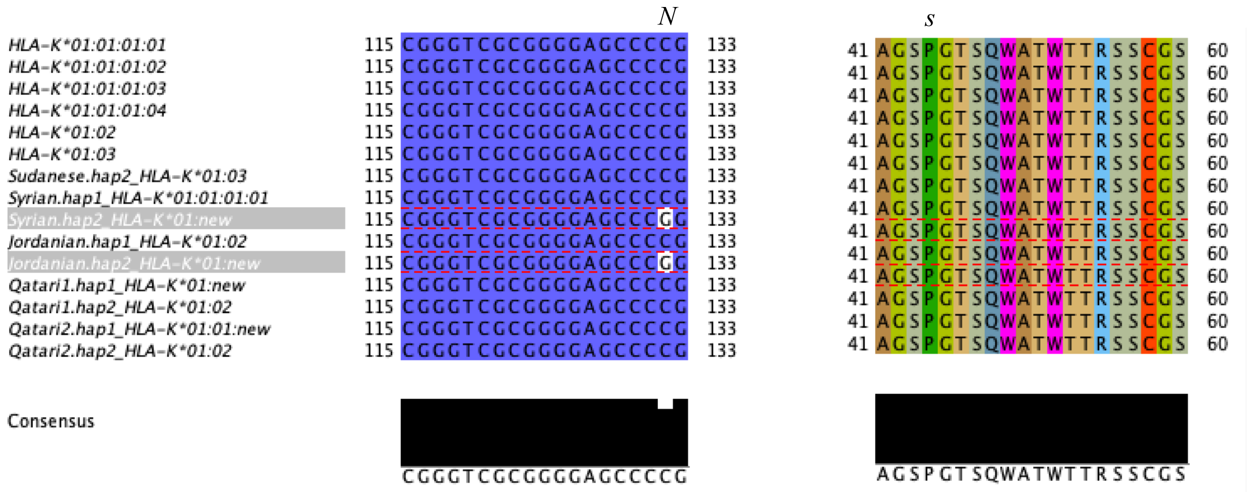
N
s
HLA-K*01:01:01:01 115 CGGGTCGCGGGGAGCCCCG 133
HLA-K*01:01:01:02 115 CGGGTCGCGGGGAGCCCCG 133
HLA-K*01:01:01:03 115 CGGGTCGCGGGGAGCCCCG 133
HLA-K*01:01:01:04 115 CGGGTCGCGGGGAGCCCCG 133
HLA-K*01:02 115 CGGGTCGCGGGGAGCCCCG 133
HLA-K*01:03 115 CGGGTCGCGGGGAGCCCCG 133
Sudanese.hap2_HLA-K*01:03 115 CGGGTCGCGGGGAGCCCCG 133
Syrian.hap1_HLA-K*01:01:01:01 115 CGGGTCGCGGGGAGCCCCG 133
Syrian.hap2_HLA-K*01:new 115 CGGGTCGCGGGGAGCCCGG 133
Jordanian.hap1_HLA-K*01:02 115 CGGGTCGCGGGGAGCCCCG 133
Jordanian.hap2_HLA-K*01:new 115 CGGGTCGCGGGGAGCCCGG 133
Qatari1.hap1_HLA-K*01:new 115 CGGGTCGCGGGGAGCCCCG 133
Qatari1.hap2_HLA-K*01:02 115 CGGGTCGCGGGGAGCCCCG 133
Qatari2.hap1_HLA-K*01:01:new 115 CGGGTCGCGGGGAGCCCCG 133
Qatari2.hap2_HLA-K*01:02 115 CGGGTCGCGGGGAGCCCCG 133
41 AGSPGTSQWATWTTRSSCGS 60
Consensus
CGGGTCGCGGGGAGCCCCG
AGSPGTSQWATWTTRSSCGS

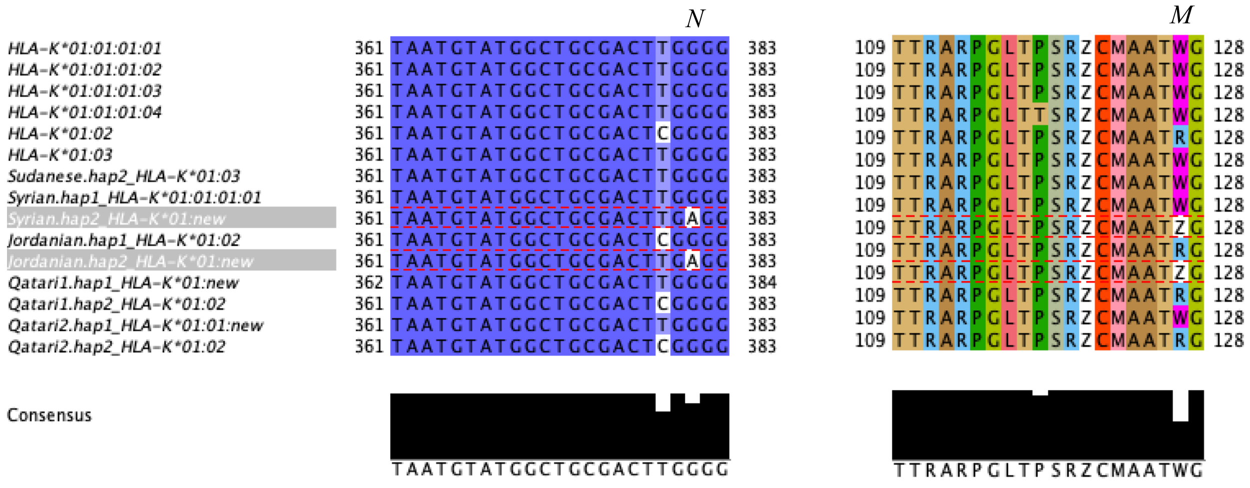
N
M
HLA-K*01:01:01:01 361 TAATGTATGGCTGCGACTTGGGG 383
HLA-K*01:01:01:02 361 TAATGTATGGCTGCGACTTGGGG 383
HLA-K*01:01:01:03 361 TAATGTATGGCTGCGACTTGGGG 383
HLA-K*01:01:01:04 361 TAATGTATGGCTGCGACTTGGGG 383
HLA-K*01:02 361 TAATGTATGGCTGCGACTCGGGG 383
HLA-K*01:03 361 TAATGTATGGCTGCGACTTGGGG 383
Sudanese.hap2_HLA-K*01:03 361 TAATGTATGGCTGCGACTTGGGG 383
Syrian.hap1_HLA-K*01:01:01:01 361 TAATGTATGGCTGCGACTTGGGG 383
Syrian.hap2_HLA-K*01:new 361 TAATGTATGGCTGCGACTTGAGG 383
Jordanian.hap1_HLA-K*01:02 361 TAATGTATGGCTGCGACTCGGGG 383
Jordanian.hap2_HLA-K*01:new 361 TAATGTATGGCTGCGACTTGAGG 383
Qatari1.hap1_HLA-K*01:new 362 TAATGTATGGCTGCGACTTGGGG 384
Qatari1.hap2_HLA-K*01:02 361 TAATGTATGGCTGCGACTCGGGG 383
Qatari2.hap1_HLA-K*01:01:new 361 TAATGTATGGCTGCGACTTGGGG 383
Qatari2.hap2_HLA-K*01:02 361 TAATGTATGGCTGCGACTCGGGG 383
109 TTRARPGLTPSRZCMAATWG 128
109 TTRARPGLTPSRZCMAATWG 128
109 TTRARPGLTPSRZCMAATWG 128
109 TTRARPGLTTSRZCMAATWG 128
109 TTRARPGLTPSRZCMAATRG 128
109 TTRARPGLTPSRZCMAATWG 128
109 TTRARPGLTPSRZCMAATWG 128
109 TTRARPGLTPSRZCMAATWG 128
109 TTRARPGLTPSRZCMAATZG 128
109 TTRARPGLTPSRZCMAATRG 128
109 TTRARPGLTPSRZCMAATZG 128
109 TTRARPGLTPSRZCMAATRG 128
109 TTRARPGLTPSRZCMAATWG 128
109 TTRARPGLTPSRZCMAATRG 128
Consensus
TAATGTATGGCTGCGACTTGGGG
TTRARPGLTPSRZCMAATWG

KIR3DL3

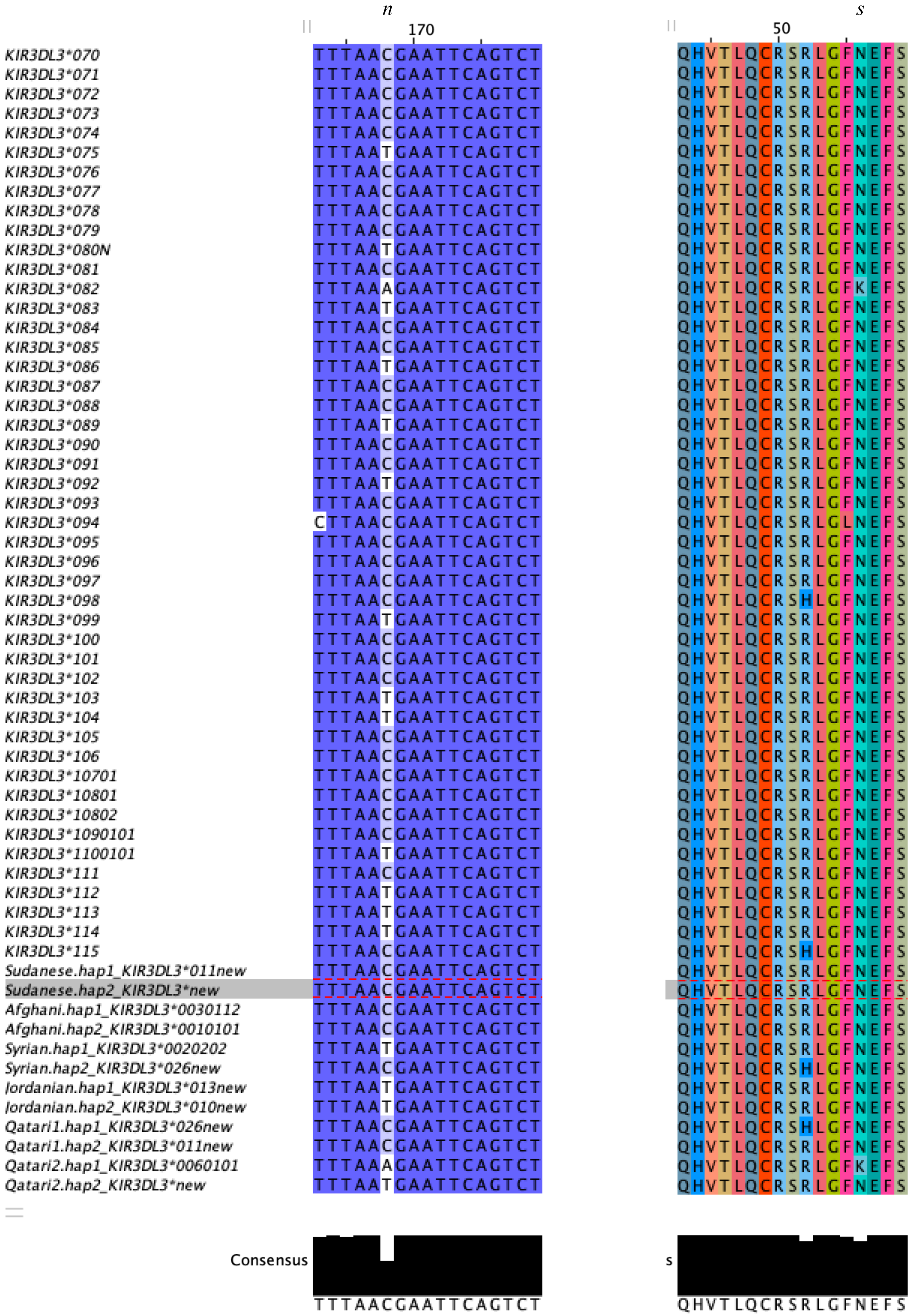
n
170
s
50
KIR3DL3*070 TTTAACGAATTCAGTCT QHVTLQCRSRLGFNEFS
KIR3DL3*071 TTTAACGAATTCAGTCT QHVTLQCRSRLGFNEFS
KIR3DL3*072 TTTAACGAATTCAGTCT QHVTLQCRSRLGFNEFS
KIR3DL3*073 TTTAACGAATTCAGTCT QHVTLQCRSRLGFNEFS
KIR3DL3*074 TTTAACGAATTCAGTCT QHVTLQCRSRLGFNEFS
KIR3DL3*075 TTTAATGAATTCAGTCT QHVTLQCRSRLGFNEFS
KIR3DL3*076 TTTAACGAATTCAGTCT QHVTLQCRSRLGFNEFS
KIR3DL3*077 TTTAACGAATTCAGTCT QHVTLQCRSRLGFNEFS
KIR3DL3*078 TTTAACGAATTCAGTCT QHVTLQCRSRLGFNEFS
KIR3DL3*079 TTTAACGAATTCAGTCT QHVTLQCRSRLGFNEFS
KIR3DL3*080N TTTAATGAATTCAGTCT QHVTLQCRSRLGFNEFS
KIR3DL3*081 TTTAACGAATTCAGTCT QHVTLQCRSRLGFNEFS
KIR3DL3*082 TTTAAAGAATTCAGTCT QHVTLQCRSRLGFKEFS
KIR3DL3*083 TTTAATGAATTCAGTCT QHVTLQCRSRLGFNEFS
KIR3DL3*084 TTTAACGAATTCAGTCT QHVTLQCRSRLGFNEFS
KIR3DL3*085 TTTAACGAATTCAGTCT QHVTLQCRSRLGFNEFS
KIR3DL3*086 TTTAATGAATTCAGTCT QHVTLQCRSRLGFNEFS
KIR3DL3*087 TTTAACGAATTCAGTCT QHVTLQCRSRLGFNEFS
KIR3DL3*088 TTTAACGAATTCAGTCT QHVTLQCRSRLGFNEFS
KIR3DL3*089 TTTAATGAATTCAGTCT QHVTLQCRSRLGFNEFS
KIR3DL3*090 TTTAACGAATTCAGTCT QHVTLQCRSRLGFNEFS
KIR3DL3*091 TTTAACGAATTCAGTCT QHVTLQCRSRLGFNEFS
KIR3DL3*092 TTTAATGAATTCAGTCT QHVTLQCRSRLGFNEFS
KIR3DL3*093 TTTAACGAATTCAGTCT QHVTLQCRSRLGFNEFS
KIR3DL3*094 CTTAACGAATTCAGTCT QHVTLQCRSRLGLNEFS
KIR3DL3*095 TTTAACGAATTCAGTCT QHVTLQCRSRLGFNEFS
KIR3DL3*096 TTTAACGAATTCAGTCT QHVTLQCRSRLGFNEFS
KIR3DL3*097 TTTAACGAATTCAGTCT QHVTLQCRSRLGFNEFS
KIR3DL3*098 TTTAACGAATTCAGTCT QHVTLQCRSHLGFNEFS
KIR3DL3*099 TTTAATGAATTCAGTCT QHVTLQCRSRLGFNEFS
KIR3DL3*100 TTTAACGAATTCAGTCT QHVTLQCRSRLGFNEFS
KIR3DL3*101 TTTAACGAATTCAGTCT QHVTLQCRSRLGFNEFS
KIR3DL3*102 TTTAACGAATTCAGTCT QHVTLQCRSRLGFNEFS
KIR3DL3*103 TTTAATGAATTCAGTCT QHVTLQCRSRLGFNEFS
KIR3DL3*104 TTTAATGAATTCAGTCT QHVTLQCRSRLGFNEFS
KIR3DL3*105 TTTAACGAATTCAGTCT QHVTLQCRSRLGFNEFS
KIR3DL3*106 TTTAACGAATTCAGTCT QHVTLQCRSRLGFNEFS
KIR3DL3*10701 TTTAACGAATTCAGTCT QHVTLQCRSRLGFNEFS
KIR3DL3*10801 TTTAACGAATTCAGTCT QHVTLQCRSRLGFNEFS
KIR3DL3*10802 TTTAACGAATTCAGTCT QHVTLQCRSRLGFNEFS
KIR3DL3*1090101 TTTAACGAATTCAGTCT QHVTLQCRSRLGFNEFS
KIR3DL3*1100101 TTTAATGAATTCAGTCT QHVTLQCRSRLGFNEFS
KIR3DL3*111 TTTAACGAATTCAGTCT QHVTLQCRSRLGFNEFS
KIR3DL3*112 TTTAATGAATTCAGTCT QHVTLQCRSRLGFNEFS
KIR3DL3*113 TTTAATGAATTCAGTCT QHVTLQCRSRLGFNEFS
KIR3DL3*114 TTTAATGAATTCAGTCT QHVTLQCRSRLGFNEFS
KIR3DL3*115 TTTAACGAATTCAGTCT QHVTLQCRSHLGFNEFS
Sudanese.hap1_KIR3DL3*011new TTTAACGAATTCAGTCT QHVTLQCRSRLGFNEFS
Sudanese.hap2_KIR3DL3*new TTTAACGAATTCAGTCT QHVTLQCRSRLGFNEFS
Afghani.hap1_KIR3DL3*0030112 TTTAACGAATTCAGTCT QHVTLQCRSRLGFNEFS
Afghani.hap2_KIR3DL3*0010101 TTTAACGAATTCAGTCT QHVTLQCRSRLGFNEFS
Syrian.hap1_KIR3DL3*0020202 TTTAATGAATTCAGTCT QHVTLQCRSRLGFNEFS
Syrian.hap2_KIR3DL3*026new TTTAACGAATTCAGTCT QHVTLQCRSHLGFNEFS
Jordanian.hap1_KIR3DL3*013new TTTAATGAATTCAGTCT QHVTLQCRSRLGFNEFS
Jordanian.hap2_KIR3DL3*010new TTTAATGAATTCAGTCT QHVTLQCRSRLGFNEFS
Qatari1.hap1_KIR3DL3*026new TTTAACGAATTCAGTCT QHVTLQCRSHLGFNEFS
Qatari1.hap2_KIR3DL3*011new TTTAACGAATTCAGTCT QHVTLQCRSRLGFNEFS
Qatari2.hap1_KIR3DL3*0060101 TTTAAAGAATTCAGTCT QHVTLQCRSRLGFKEFS
Qatari2.hap2_KIR3DL3*new TTTAATGAATTCAGTCT QHVTLQCRSRLGFNEFS
Consensus
TTTAACGAATTCAGTCT
s
QHVTLQCRSRLGFNEFS

TAP1

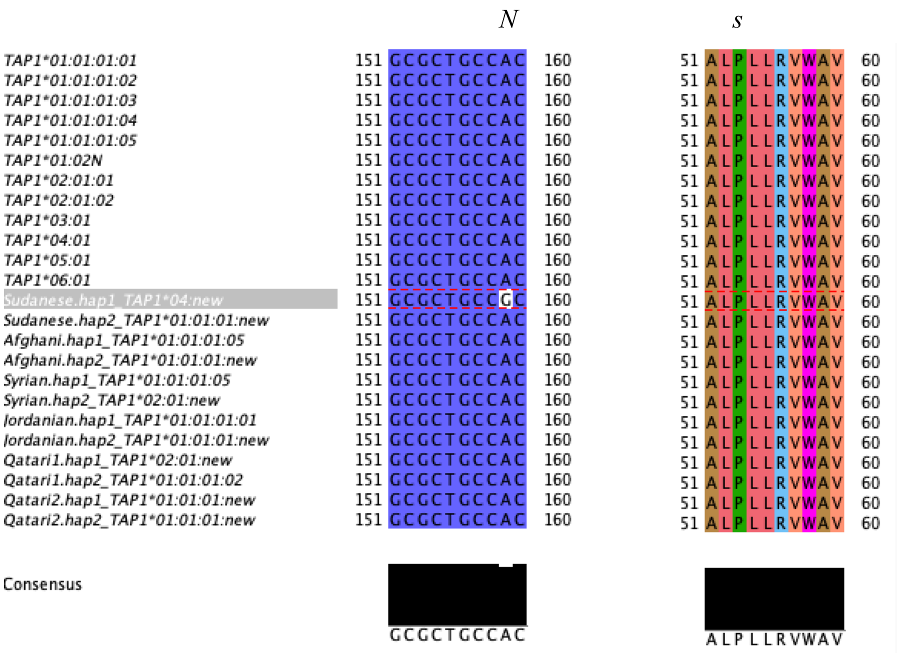

N
s
TAP1*01:01:01:01 151 GCGCTGCCAC 160 51 ALPLLRVWAV 60
TAP1*01:01:01:02 151 GCGCTGCCAC 160 51 ALPLLRVWAV 60
TAP1*01:01:01:03 151 GCGCTGCCAC 160 51 ALPLLRVWAV 60
TAP1*01:01:01:04 151 GCGCTGCCAC 160 51 ALPLLRVWAV 60
TAP1*01:01:01:05 151 GCGCTGCCAC 160 51 ALPLLRVWAV 60
TAP1*01:02N 151 GCGCTGCCAC 160 51 ALPLLRVWAV 60
TAP1*02:01:01 151 GCGCTGCCAC 160 51 ALPLLRVWAV 60
TAP1*02:01:02 151 GCGCTGCCAC 160 51 ALPLLRVWAV 60
TAP1*03:01 151 GCGCTGCCAC 160 51 ALPLLRVWAV 60
TAP1*04:01 151 GCGCTGCCAC 160 51 ALPLLRVWAV 60
TAP1*05:01 151 GCGCTGCCAC 160 51 ALPLLRVWAV 60
TAP1*06:01 151 GCGCTGCCAC 160 51 ALPLLRVWAV 60
Sudanese.hap1_TAP1*04:new 151 GCGCTGCCGC 160 51 ALPLLRVWAV 60
Sudanese.hap2_TAP1*01:01:01:new 151 GCGCTGCCAC 160 51 ALPLLRVWAV 60
Afghani.hap1_TAP1*01:01:01:05 151 GCGCTGCCAC 160 51 ALPLLRVWAV 60
Afghani.hap2_TAP1*01:01:01:new 151 GCGCTGCCAC 160 51 ALPLLRVWAV 60
Syrian.hap1_TAP1*01:01:01:05 151 GCGCTGCCAC 160 51 ALPLLRVWAV 60
Syrian.hap2_TAP1*02:01:new 151 GCGCTGCCAC 160 51 ALPLLRVWAV 60
Jordanian.hap1_TAP1*01:01:01:01 151 GCGCTGCCAC 160 51 ALPLLRVWAV 60
Jordanian.hap2_TAP1*01:01:01:new 151 GCGCTGCCAC 160 51 ALPLLRVWAV 60
Qatari1.hap1_TAP1*02:01:new 151 GCGCTGCCAC 160 51 ALPLLRVWAV 60
Qatari1.hap2_TAP1*01:01:01:02 151 GCGCTGCCAC 160 51 ALPLLRVWAV 60
Qatari2.hap1_TAP1*01:01:01:new 151 GCGCTGCCAC 160 51 ALPLLRVWAV 60
Qatari2.hap2_TAP1*01:01:01:new 151 GCGCTGCCAC 160 51 ALPLLRVWAV 60
Consensus GCGCTGCCAC ALPLLRVWAV

TAP2

		n			*s*	
*TAP2*01:01:01*	1151	ACTTGGGGGT	1160	381	RVLHLGVQML	390
*TAP2*01:01:02*	1151	ACTTGGGGGT	1160	381	RVLHLGVQML	390
*TAP2*01:01:03:01*	1151	ACTTGGGGGT	1160	381	RVLHLGVQML	390
*TAP2*01:01:03:02*	1151	ACTTGGGGGT	1160	381	RVLHLGVQML	390
*TAP2*01:01:03:03*	1151	ACTTGGGGGT	1160	381	RVLHLGVQML	390
*TAP2*01:02*	1151	ACTTGGGTGT	1160	381	RVLHLGVQML	390
*TAP2*01:03*	961	ACTTGGGTGT	970	317	RVLHLGVQML	326
*TAP2*01:04*	1151	ACTTGGGGGT	1160	381	RVLHLGVQML	390
*TAP2*02:01:01*	1151	ACTTGGGGGT	1160	381	RVLHLGVQML	390
*TAP2*02:01:02:01*	1151	ACTTGGGGGT	1160	381	RVLHLGVQML	390
*TAP2*02:01:02:02*	1151	ACTTGGGGGT	1160	381	RVLHLGVQML	390
*TAP2*02:01:02:03*	1151	ACTTGGGGGT	1160	381	RVLHLGVQML	390
*Sudanese.hap1_TAP2*01:new*	1151	ACTTGGGGGT	1160	381	RVLHLGVQML	390
*Sudanese.hap2_TAP2*02:01:02:new*	1151	ACTTGGGGGT	1160	381	RVLHLGVQML	390
*Afghani.hap1_TAP2*02:01:new*	1151	ACTTGGGTGT	1160	381	RVLHLGVQML	390
*Afghani.hap2_TAP2*01:01:new*	1151	ACTTGGGTGT	1160	381	RVLHLGVQML	390
*Syrian.hap1_TAP2*01:01:new*	1151	ACTTGGGTGT	1160	381	RVLHLGVQML	390
*Syrian.hap2_TAP2*01:01:03:new*	1151	ACTTGGGGGT	1160	381	RVLHLGVQML	390
*Jordanian.hap1_TAP2*01:01:03:new*	1151	ACTTGGGGGT	1160	381	RVLHLGVQML	390
*Jordanian.hap2_TAP2*02:01:new*	1151	ACTTGGGGGT	1160	381	RVLHLGVQML	390
*Qatari1.hap1_TAP2*01:01:03:new*	1151	ACTTGGGGGT	1160	381	RVLHLGVQML	390
*Qatari1.hap2_TAP2*01:new*	1151	ACTTGGGTGT	1160	381	RVLHLGVQML	390
*Qatari2.hap1_TAP2*01:01:03:new*	1151	ACTTGGGGGT	1160	381	RVLHLGVQML	390
*Qatari2.hap2_TAP2*02:01:02:new*	1151	ACTTGGGGGT	1160	381	RVLHLGVQML	390

Consensus

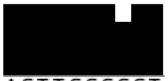

ACTTGGGGGT

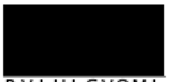

RVLHLGVQML

		N			*M*	
*TAP2*01:01:01*	1411	CAAGACGTCT	1420	471	QDVSFAYPNR	480
*TAP2*01:01:02*	1411	CAAGACGTCT	1420	471	QDVSFAYPNR	480
*TAP2*01:01:03:01*	1411	CAAGACGTCT	1420	471	QDVSFAYPNR	480
*TAP2*01:01:03:02*	1411	CAAGACGTCT	1420	471	QDVSFAYPNR	480
*TAP2*01:01:03:03*	1411	CAAGACGTCT	1420	471	QDVSFAYPNR	480
*TAP2*01:02*	1411	CAAGACGTCT	1420	471	QDVSFAYPNR	480
*TAP2*01:03*	1221	CAAGACGTCT	1230	407	QDVSFAYPNR	416
*TAP2*01:04*	1411	CAAGACGTCT	1420	471	QDVSFAYPNR	480
*TAP2*02:01:01*	1411	CAAGACGTCT	1420	471	QDVSFAYPNR	480
*TAP2*02:01:02:01*	1411	CAAGACGTCT	1420	471	QDVSFAYPNR	480
*TAP2*02:01:02:02*	1411	CAAGACGTCT	1420	471	QDVSFAYPNR	480
*TAP2*02:01:02:03*	1411	CAAGACGTCT	1420	471	QDVSFAYPNR	480
*Sudanese.hap1_TAP2*01:new*	1411	CAAGACGTCT	1420	471	QDVSFAYPNR	480
*Sudanese.hap2_TAP2*02:01:02:new*	1411	CAAGACGTCT	1420	471	QDVSFAYPNR	480
*Afghani.hap1_TAP2*02:01:new*	1411	CAAGACGTCT	1420	471	QDVSFAYPNR	480
*Afghani.hap2_TAP2*01:01:new*	1411	CAAGACGTCT	1420	471	QDVSFAYPNR	480
*Syrian.hap1_TAP2*01:01:new*	1411	CAAGACGTCT	1420	471	QDVSFAYPNR	480
*Syrian.hap2_TAP2*01:01:03:new*	1411	CAAGACGTCT	1420	471	QDVSFAYPNR	480
*Jordanian.hap1_TAP2*01:01:03:new*	1411	CAAGACGTCT	1420	471	QDVSFAYPNR	480
*Jordanian.hap2_TAP2*02:01:new*	1411	CAAGACGTCT	1420	471	QDVSFAYPNR	480
*Qatari1.hap1_TAP2*01:01:03:new*	1411	CAAGACGTCT	1420	471	QDVSFAYPNR	480
*Qatari1.hap2_TAP2*01:new*	1411	CAAGACATCT	1420	471	QDISFAYPNR	480
*Qatari2.hap1_TAP2*01:01:03:new*	1411	CAAGACGTCT	1420	471	QDVSFAYPNR	480
*Qatari2.hap2_TAP2*02:01:02:new*	1411	CAAGACGTCT	1420	471	QDVSFAYPNR	480

Consensus

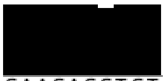

CAAGACGTCT

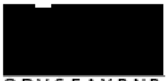

QDVSFAYPNR

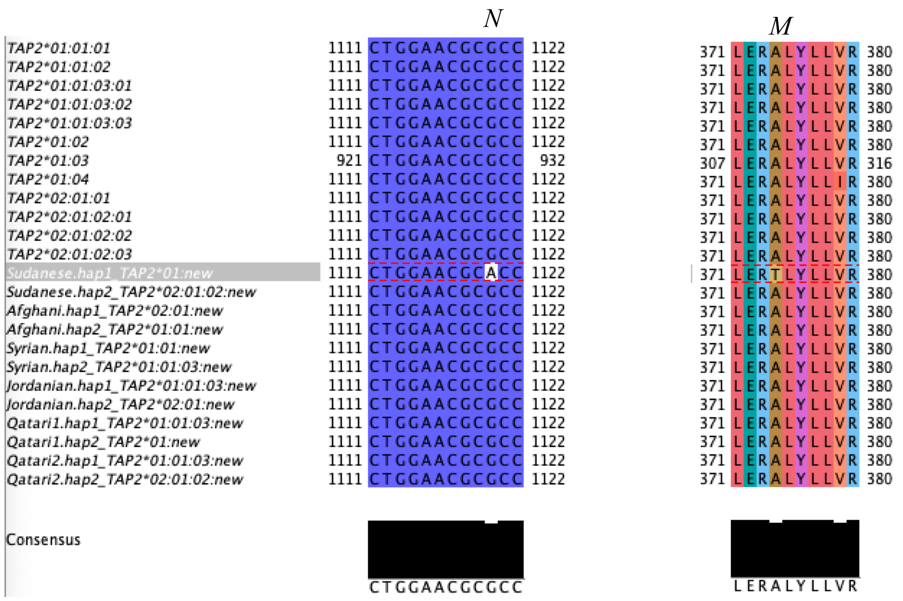
N
M
TAP2*01:01:01 1111 CTGGAACGCGCC 1122 371 LERALYLLVR 380
TAP2*01:01:02 1111 CTGGAACGCGCC 1122 371 LERALYLLVR 380
TAP2*01:01:03:01 1111 CTGGAACGCGCC 1122 371 LERALYLLVR 380
TAP2*01:01:03:02 1111 CTGGAACGCGCC 1122 371 LERALYLLVR 380
TAP2*01:01:03:03 1111 CTGGAACGCGCC 1122 371 LERALYLLVR 380
TAP2*01:02 1111 CTGGAACGCGCC 1122 371 LERALYLLVR 380
TAP2*01:03 921 CTGGAACGCGCC 932 307 LERALYLLVR 316
TAP2*01:04 1111 CTGGAACGCGCC 1122 371 LERALYLLIR 380
TAP2*02:01:01 1111 CTGGAACGCGCC 1122 371 LERALYLLVR 380
TAP2*02:01:02:01 1111 CTGGAACGCGCC 1122 371 LERALYLLVR 380
TAP2*02:01:02:02 1111 CTGGAACGCGCC 1122 371 LERALYLLVR 380
TAP2*02:01:02:03 1111 CTGGAACGCGCC 1122 371 LERALYLLVR 380
Sudanese.hap1_TAP2*01:new 1111 CTGGAACGCACC 1122 371 LERTLYLLVR 380
Sudanese.hap2_TAP2*02:01:02:new 1111 CTGGAACGCGCC 1122 371 LERALYLLVR 380
Afghani.hap1_TAP2*02:01:new 1111 CTGGAACGCGCC 1122 371 LERALYLLVR 380
Afghani.hap2_TAP2*01:01:new 1111 CTGGAACGCGCC 1122 371 LERALYLLVR 380
Syrian.hap1_TAP2*01:01:new 1111 CTGGAACGCGCC 1122 371 LERALYLLVR 380
Syrian.hap2_TAP2*01:01:03:new 1111 CTGGAACGCGCC 1122 371 LERALYLLVR 380
Jordanian.hap1_TAP2*01:01:03:new 1111 CTGGAACGCGCC 1122 371 LERALYLLVR 380
Jordanian.hap2_TAP2*02:01:new 1111 CTGGAACGCGCC 1122 371 LERALYLLVR 380
Qatari1.hap1_TAP2*01:01:03:new 1111 CTGGAACGCGCC 1122 371 LERALYLLVR 380
Qatari1.hap2_TAP2*01:new 1111 CTGGAACGCGCC 1122 371 LERALYLLVR 380
Qatari2.hap1_TAP2*01:01:03:new 1111 CTGGAACGCGCC 1122 371 LERALYLLVR 380
Qatari2.hap2_TAP2*02:01:02:new 1111 CTGGAACGCGCC 1122 371 LERALYLLVR 380
Consensus
CTGGAACGCGCC
LERALYLLVR

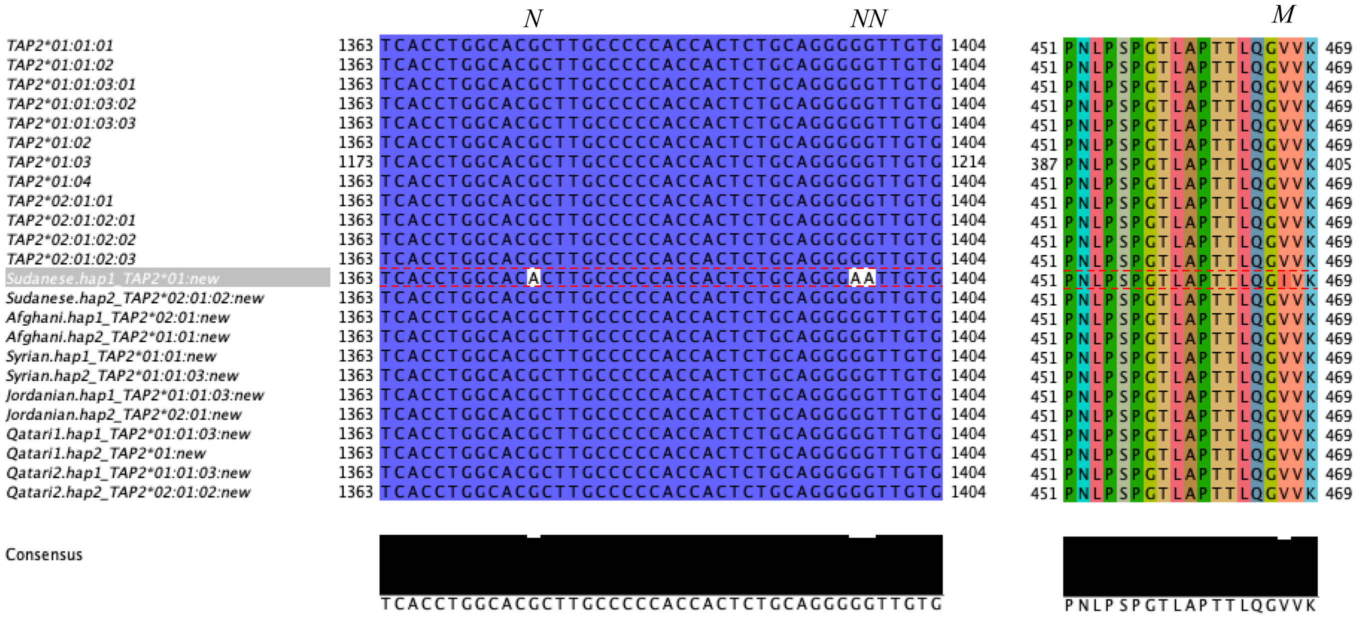
N
NN
M
TAP2*01:01:01 1363 TCACCTGGCACGCTTGCCCCCACCACTCTGCAGGGGGTTGTG 1404 451 PNLPSPGTLAPTTLQGVVK 469
TAP2*01:01:02 1363 TCACCTGGCACGCTTGCCCCCACCACTCTGCAGGGGGTTGTG 1404 451 PNLPSPGTLAPTTLQGVVK 469
TAP2*01:01:03:01 1363 TCACCTGGCACGCTTGCCCCCACCACTCTGCAGGGGGTTGTG 1404 451 PNLPSPGTLAPTTLQGVVK 469
TAP2*01:01:03:02 1363 TCACCTGGCACGCTTGCCCCCACCACTCTGCAGGGGGTTGTG 1404 451 PNLPSPGTLAPTTLQGVVK 469
TAP2*01:01:03:03 1363 TCACCTGGCACGCTTGCCCCCACCACTCTGCAGGGGGTTGTG 1404 451 PNLPSPGTLAPTTLQGVVK 469
TAP2*01:02 1363 TCACCTGGCACGCTTGCCCCCACCACTCTGCAGGGGGTTGTG 1404 451 PNLPSPGTLAPTTLQGVVK 469
TAP2*01:03 1173 TCACCTGGCACGCTTGCCCCCACCACTCTGCAGGGGGTTGTG 1214 387 PNLPSPGTLAPTTLQGVVK 405
TAP2*01:04 1363 TCACCTGGCACGCTTGCCCCCACCACTCTGCAGGGGGTTGTG 1404 451 PNLPSPGTLAPTTLQGVVK 469
TAP2*02:01:01 1363 TCACCTGGCACGCTTGCCCCCACCACTCTGCAGGGGGTTGTG 1404 451 PNLPSPGTLAPTTLQGVVK 469
TAP2*02:01:02:01 1363 TCACCTGGCACGCTTGCCCCCACCACTCTGCAGGGGGTTGTG 1404 451 PNLPSPGTLAPTTLQGVVK 469
TAP2*02:01:02:02 1363 TCACCTGGCACGCTTGCCCCCACCACTCTGCAGGGGGTTGTG 1404 451 PNLPSPGTLAPTTLQGVVK 469
TAP2*02:01:02:03 1363 TCACCTGGCACGCTTGCCCCCACCACTCTGCAGGGGGTTGTG 1404 451 PNLPSPGTLAPTTLQGVVK 469
Sudanese.hap1_TAP2*01:new 1363 TCACCTGGCACACTTGCCCCCACCACTCTGCAGGGAATTGTG 1404 451 PNLPSPGTLAPTTLQGIVK 469
Sudanese.hap2_TAP2*02:01:02:new 1363 TCACCTGGCACGCTTGCCCCCACCACTCTGCAGGGGGTTGTG 1404 451 PNLPSPGTLAPTTLQGVVK 469
Afghani.hap1_TAP2*02:01:new 1363 TCACCTGGCACGCTTGCCCCCACCACTCTGCAGGGGGTTGTG 1404 451 PNLPSPGTLAPTTLQGVVK 469
Afghani.hap2_TAP2*01:01:new 1363 TCACCTGGCACGCTTGCCCCCACCACTCTGCAGGGGGTTGTG 1404 451 PNLPSPGTLAPTTLQGVVK 469
Syrian.hap1_TAP2*01:01:new 1363 TCACCTGGCACGCTTGCCCCCACCACTCTGCAGGGGGTTGTG 1404 451 PNLPSPGTLAPTTLQGVVK 469
Syrian.hap2_TAP2*01:01:03:new 1363 TCACCTGGCACGCTTGCCCCCACCACTCTGCAGGGGGTTGTG 1404 451 PNLPSPGTLAPTTLQGVVK 469
Jordanian.hap1_TAP2*01:01:03:new 1363 TCACCTGGCACGCTTGCCCCCACCACTCTGCAGGGGGTTGTG 1404 451 PNLPSPGTLAPTTLQGVVK 469
Jordanian.hap2_TAP2*02:01:new 1363 TCACCTGGCACGCTTGCCCCCACCACTCTGCAGGGGGTTGTG 1404 451 PNLPSPGTLAPTTLQGVVK 469
Qatari1.hap1_TAP2*01:01:03:new 1363 TCACCTGGCACGCTTGCCCCCACCACTCTGCAGGGGGTTGTG 1404 451 PNLPSPGTLAPTTLQGVVK 469
Qatari1.hap2_TAP2*01:new 1363 TCACCTGGCACGCTTGCCCCCACCACTCTGCAGGGGGTTGTG 1404 451 PNLPSPGTLAPTTLQGVVK 469
Qatari2.hap1_TAP2*01:01:03:new 1363 TCACCTGGCACGCTTGCCCCCACCACTCTGCAGGGGGTTGTG 1404 451 PNLPSPGTLAPTTLQGVVK 469
Qatari2.hap2_TAP2*02:01:02:new 1363 TCACCTGGCACGCTTGCCCCCACCACTCTGCAGGGGGTTGTG 1404 451 PNLPSPGTLAPTTLQGVVK 469
Consensus
TCACCTGGCACGCTTGCCCCCACCACTCTGCAGGGGGTTGTG
PNLPSPGTLAPTTLQGVVK

Supplementary Fig. 16. Phylogenetic trees of *HLA* and *KIR* genes with novel alleles having mutation in the CDS region. Colored circles indicate various child samples. Novel alleles are labeled showing those with mutations in the CDS region (black squares) and those with mutations in other part of the sequence (grey squares).

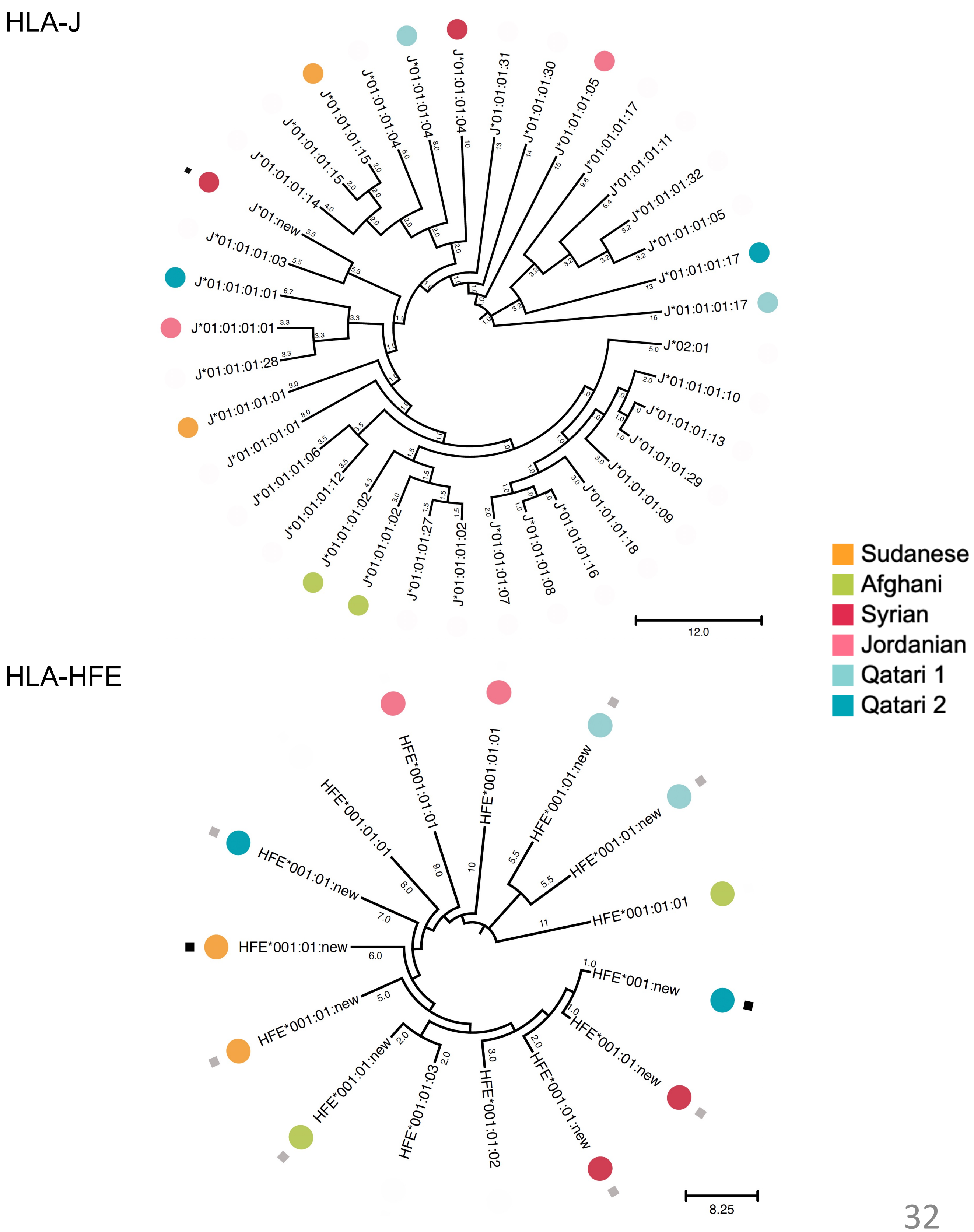

HLA-K

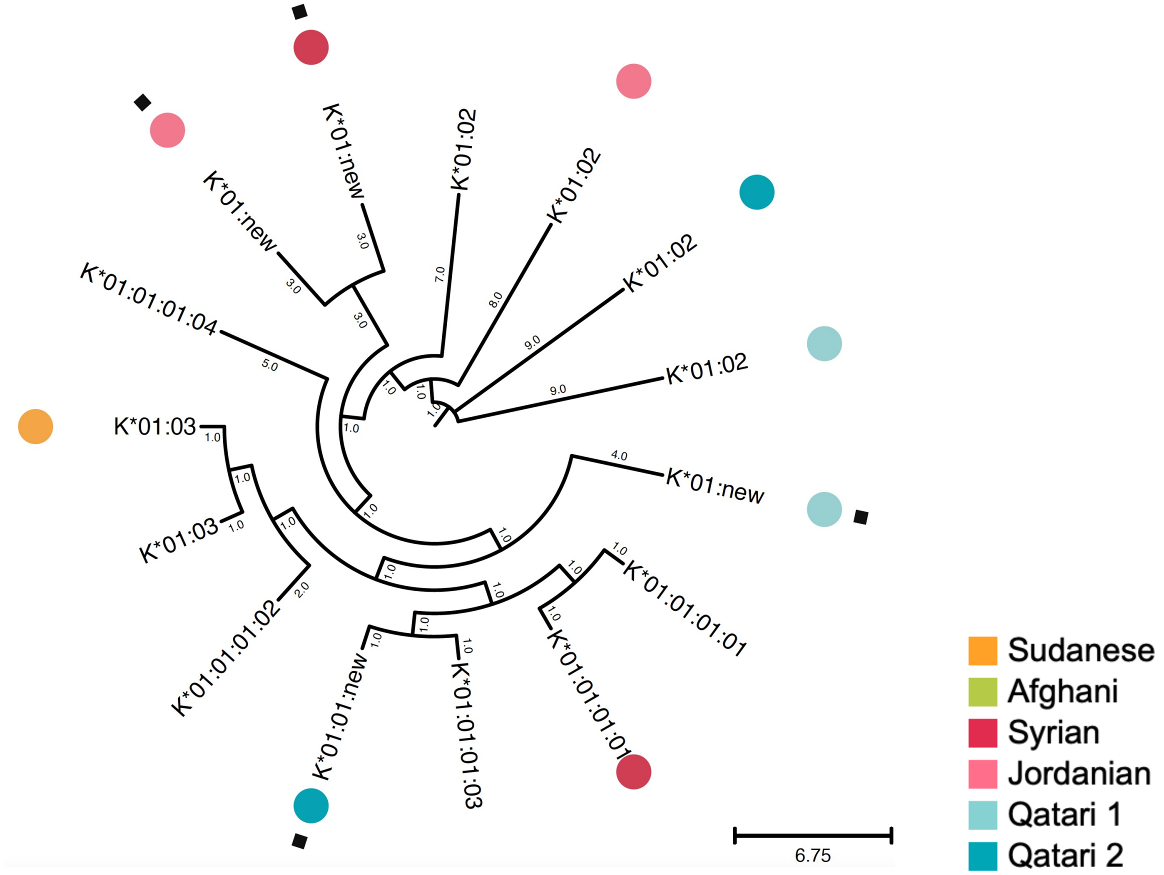

KIR3DL3

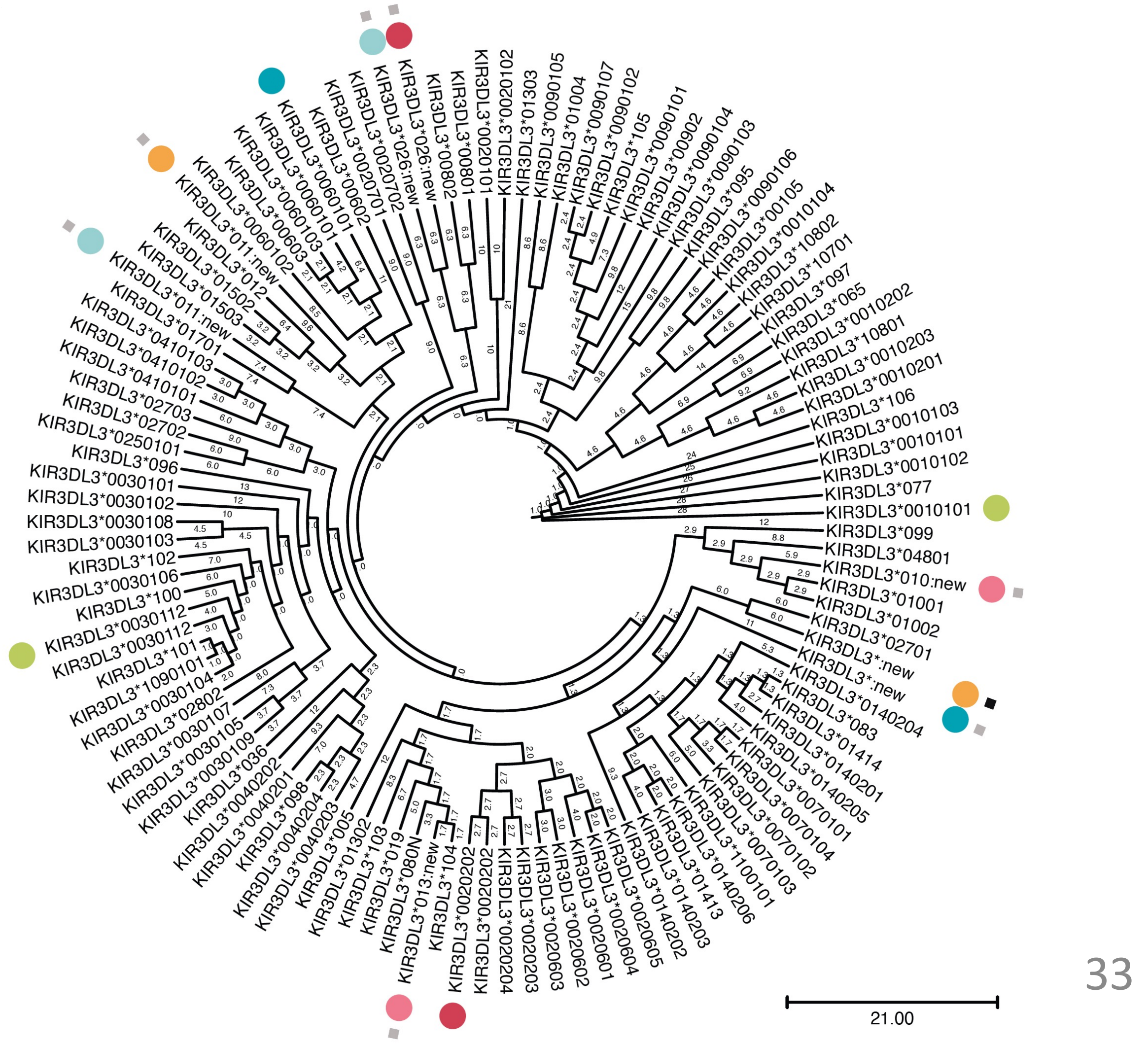

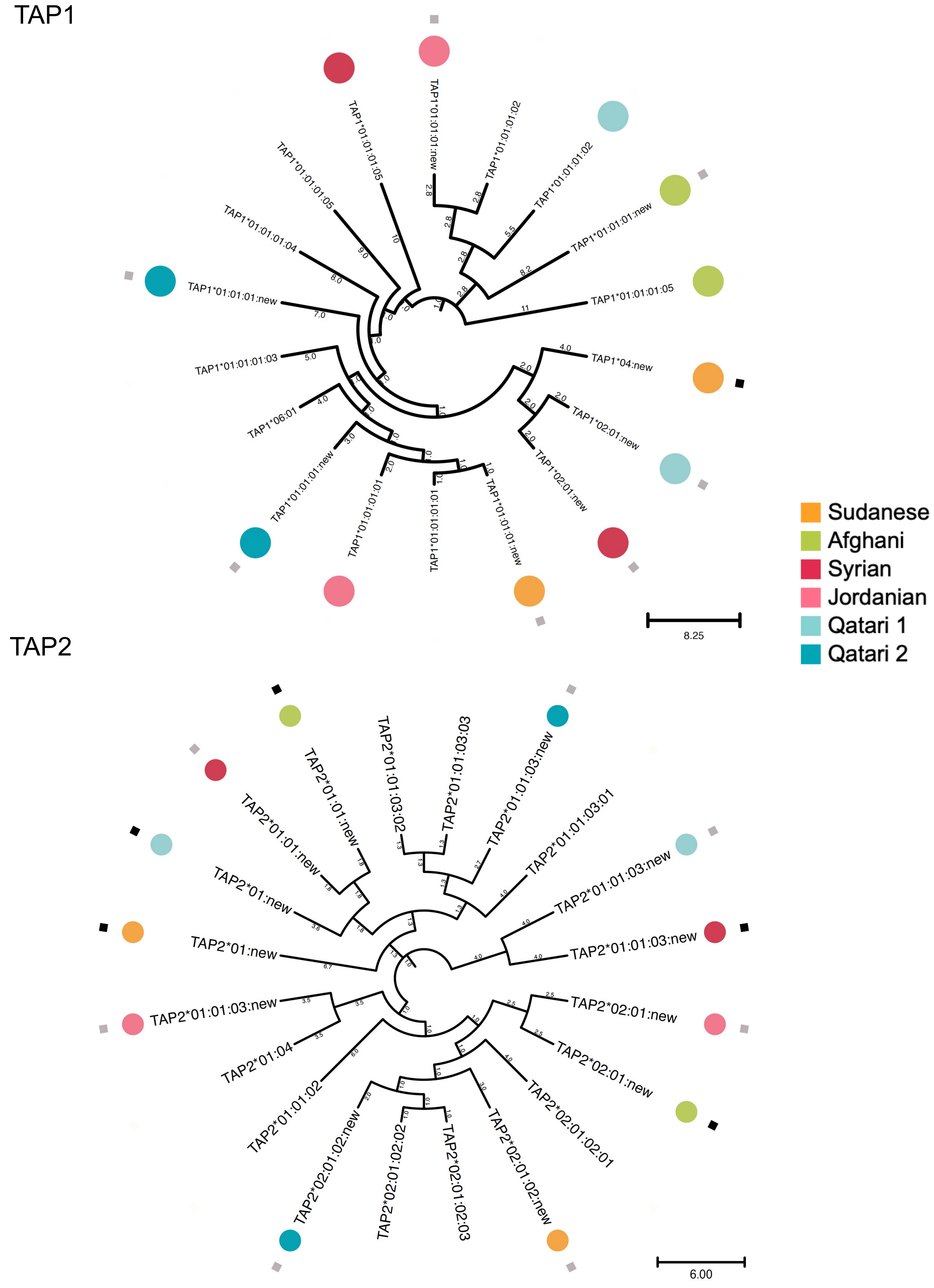
TAP1
TAP1*01:01:01:new
TAP1*01:01:01:02
TAP1*01:01:01:02
TAP1*01:01:01:new
TAP1*01:01:01:05
TAP1*04:new
TAP1*02:01:new
TAP1*02:01:new
TAP1*01:01:01:new
TAP1*01:01:01:01
TAP1*01:01:01:01
TAP1*01:01:01:new
TAP1*06:01
TAP1*01:01:01:03
TAP1*01:01:01:new
TAP1*01:01:01:04
TAP1*01:01:01:05
TAP1*01:01:01:05
8.25
TAP2
TAP2*01:01:03:02
TAP2*01:01:03:03
TAP2*01:01:03:new
TAP2*01:01:03:01
TAP2*01:01:03:new
TAP2*01:01:03:new
TAP2*02:01:new
TAP2*02:01:new
TAP2*02:01:02:01
TAP2*02:01:02:new
TAP2*02:01:02:03
TAP2*02:01:02:02
TAP2*02:01:02:new
TAP2*01:01:02
TAP2*01:04
TAP2*01:01:03:new
TAP2*01:new
TAP2*01:new
TAP2*01:01:new
TAP2*01:01:new
6.00
Sudanese
Afghani
Syrian
Jordanian
Qatari 1
Qatari 2

Supplementary Fig. 17: Variant calls using read-based and assembly-based methods. The plot shows a comparison of variant counts identified by DeepVariant (read-based method) and PAV (assembly-based method) against CHM13 and GRCh38.

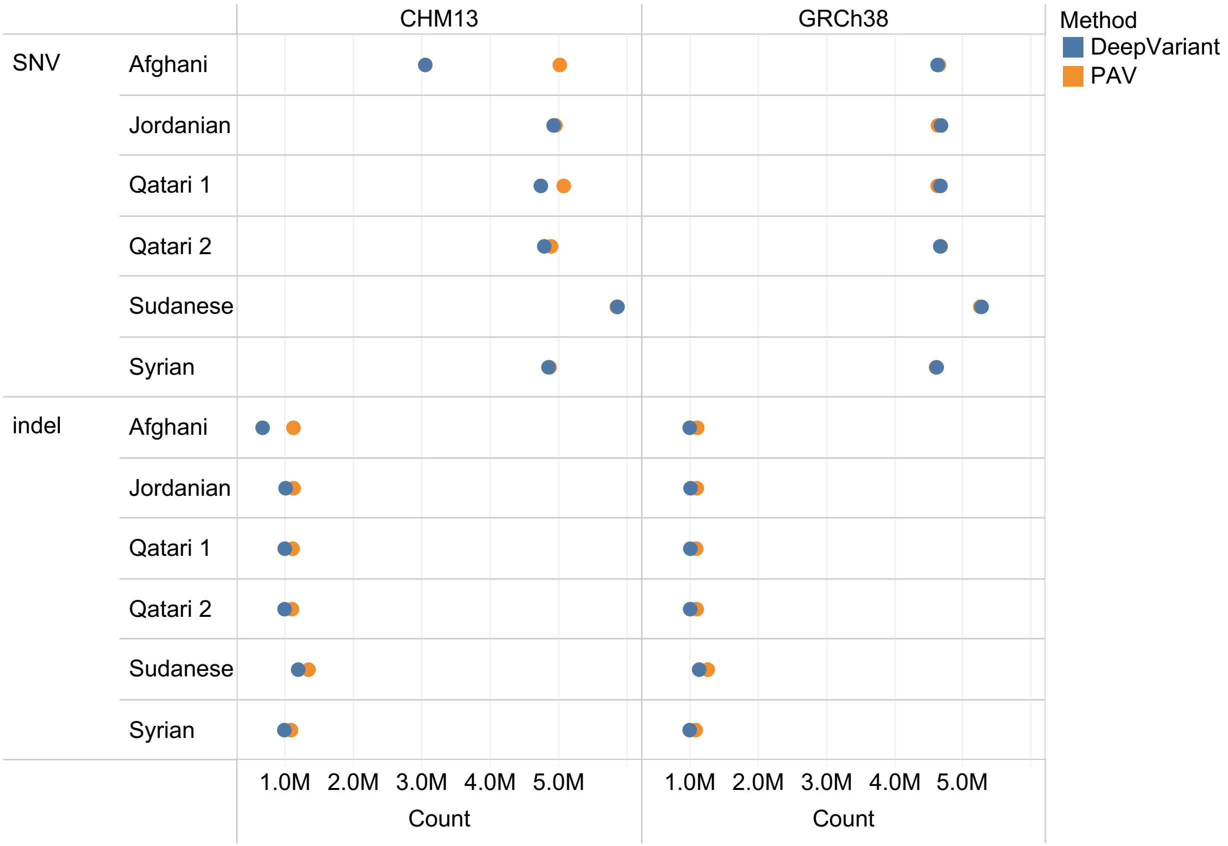

Supplementary Fig. 18: Variant calls against CHM13 for child assemblies. (a) SNV and indel calls. **(b)** SV calls.

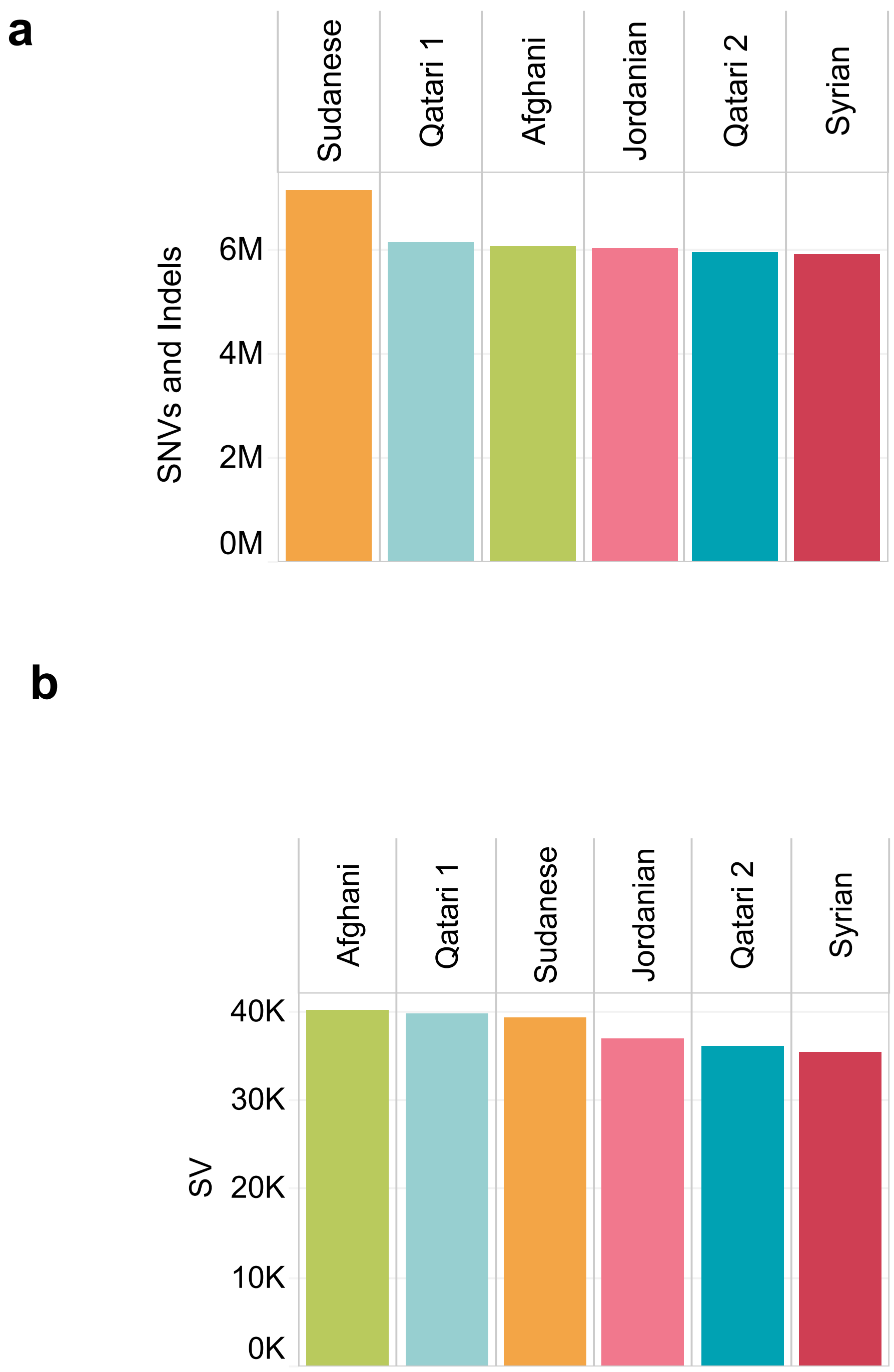

Supplementary Fig. 19: Variant calls binned per local ancestry.

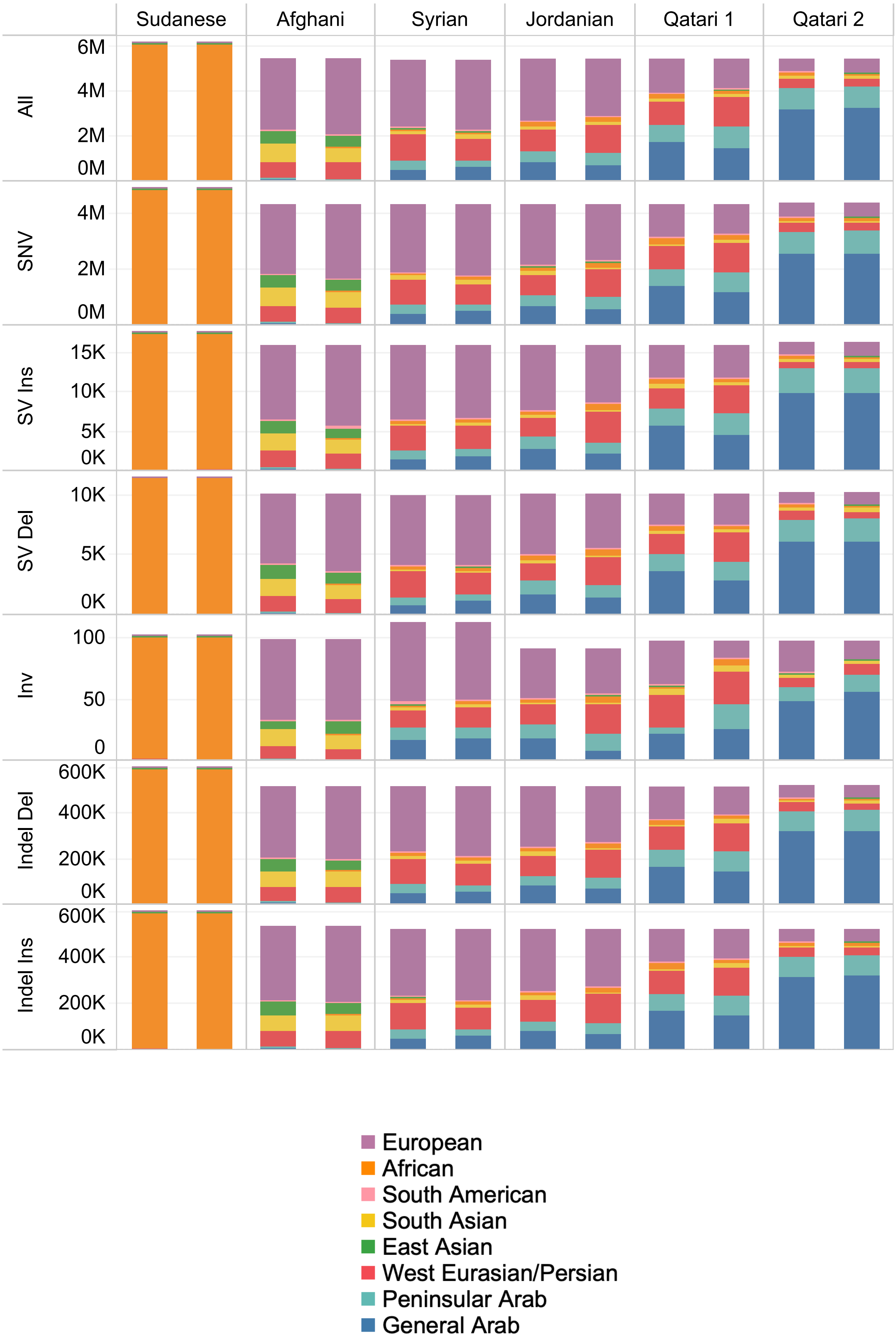

Supplementary Fig. 20: Genetic variation as a function of local ancestry (a) SNV, Indel and SV insertions and deletions per 1Mb of local ancestry segment across chromosome 10. Bar plot showing number of variant calls per 1 Mbp on chr10 across haplotypes. The color represents different ancestries, and the size of each bar corresponds to the length of the ancestry stretch. The horizontal dotted line for each SV type indicates the average count of variant per Mb. **(b)** Bar plot showing median counts of variants per MB relative to African segments in the same subjects aggregated by family, for various ancestries.

a

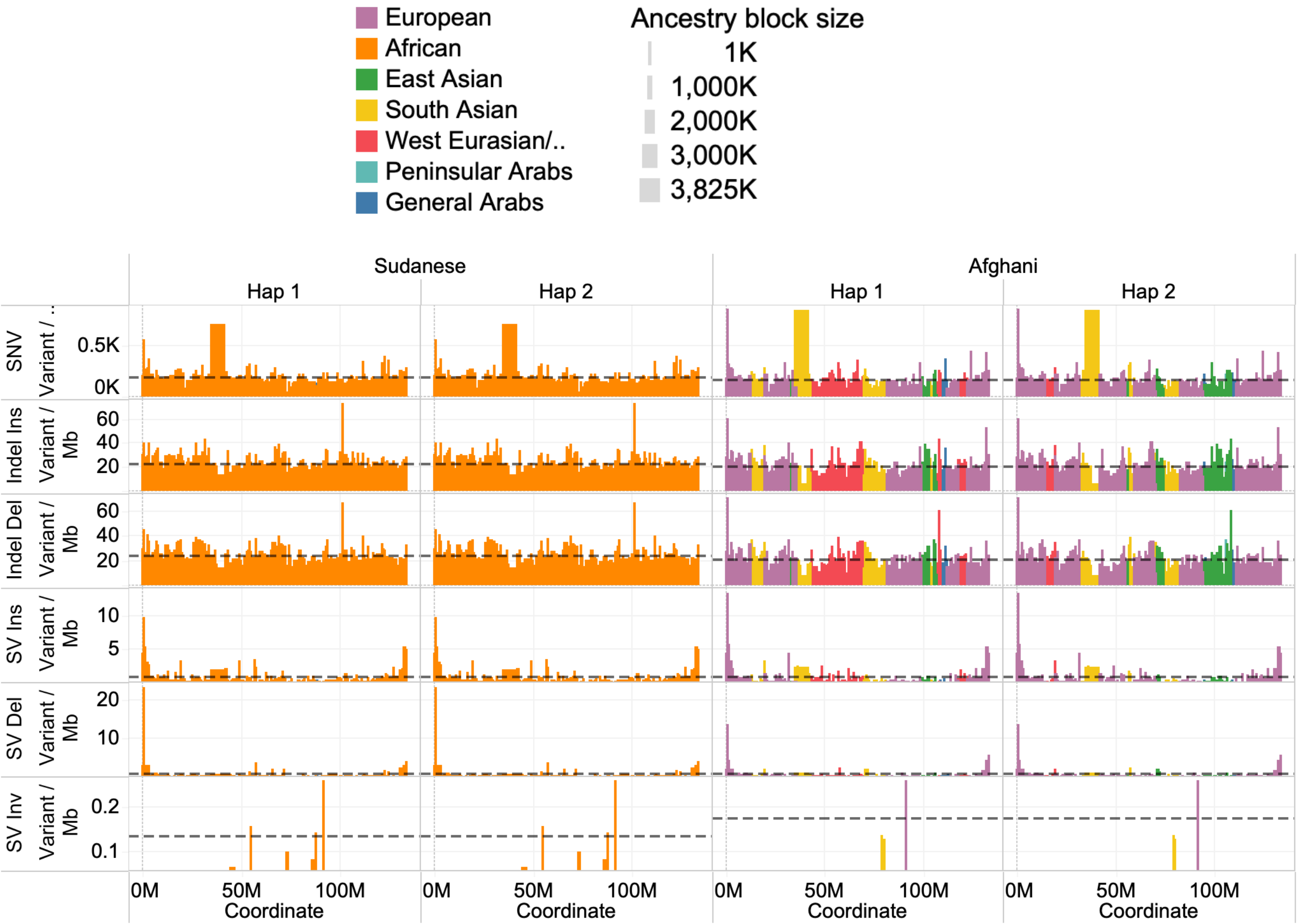

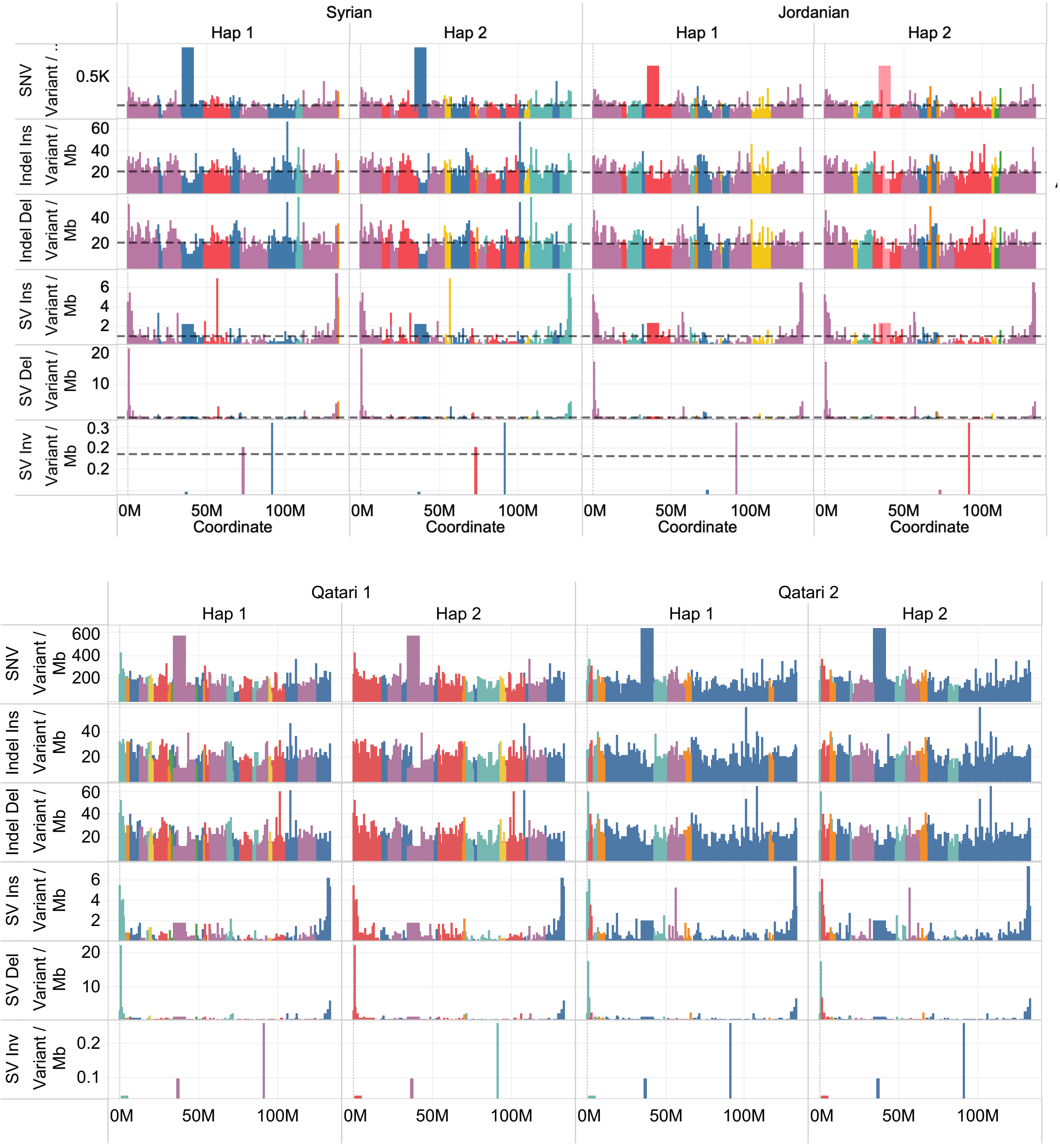

Syrian
Jordanian
Hap 1
Hap 2
SNV Variant / ..
0.5K
Indel Ins Variant / Mb
60
40
20
Indel Del Variant / Mb
40
20
SV Ins Variant / Mb
6
4
2
SV Del Variant / Mb
20
10
SV Inv Variant / Mb
0.3
0.2
0.2
0M
50M
100M
Coordinate
Qatari 1
Qatari 2
SNV Variant / Mb
600
400
200
Indel Ins Variant / Mb
40
20
Indel Del Variant / Mb
60
40
20
SV Ins Variant / Mb
6
4
2
SV Del Variant / Mb
20
10
SV Inv Variant / Mb
0.2
0.1

b

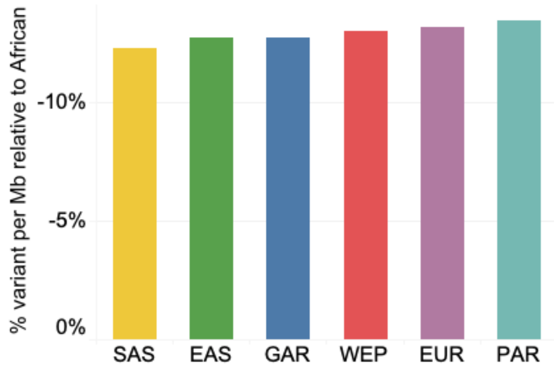

% variant per Mb relative to African
-10%
-5%
0%
SAS
EAS
GAR
WEP
EUR
PAR

Supplementary Fig. S21: Runs of Homozygosity (ROH) per gene type.
Total count of genes per gene type in the ROH segments.

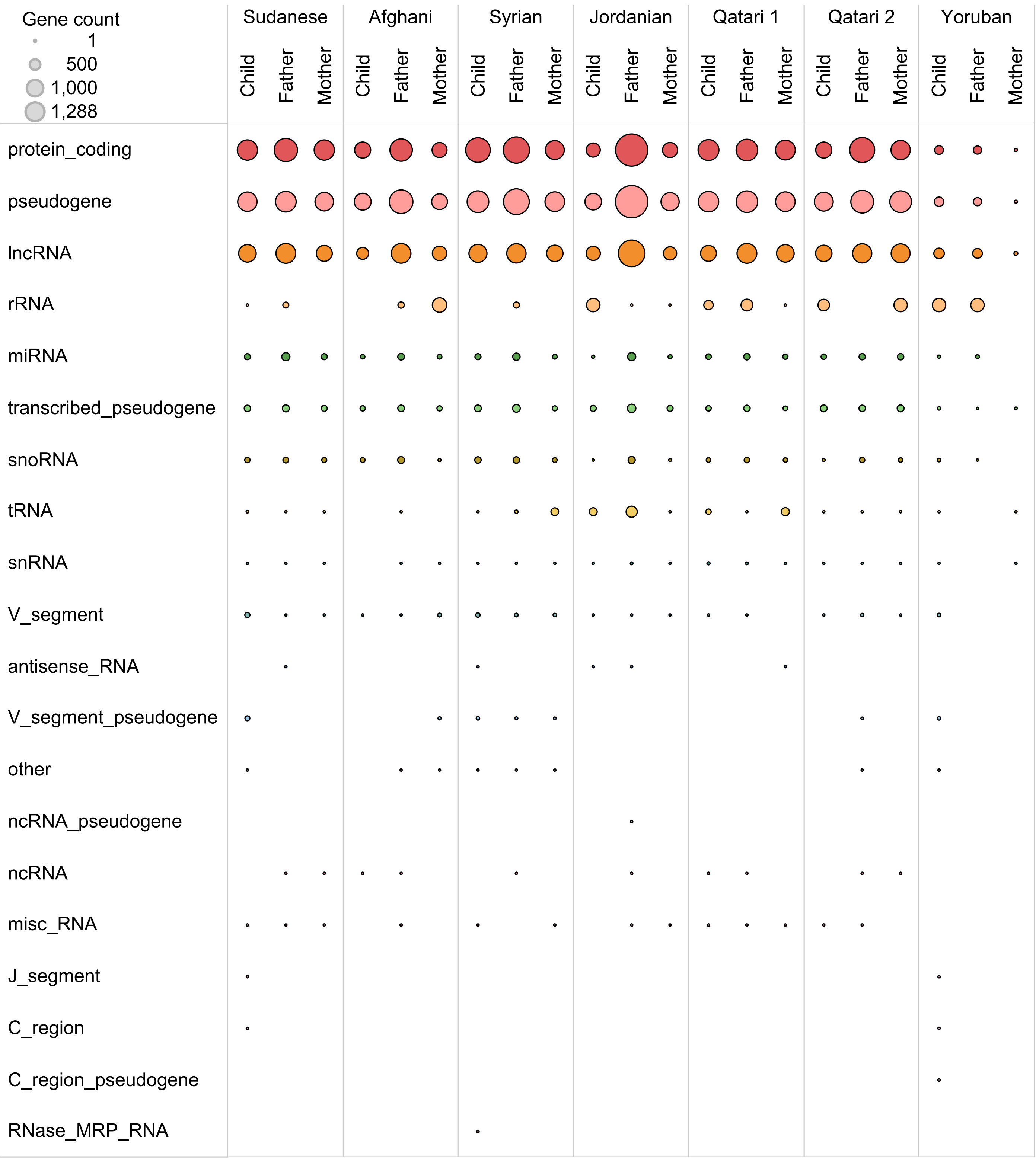

Supplementary Fig. 22: Correlation between ROH size and count of encompassed genes.

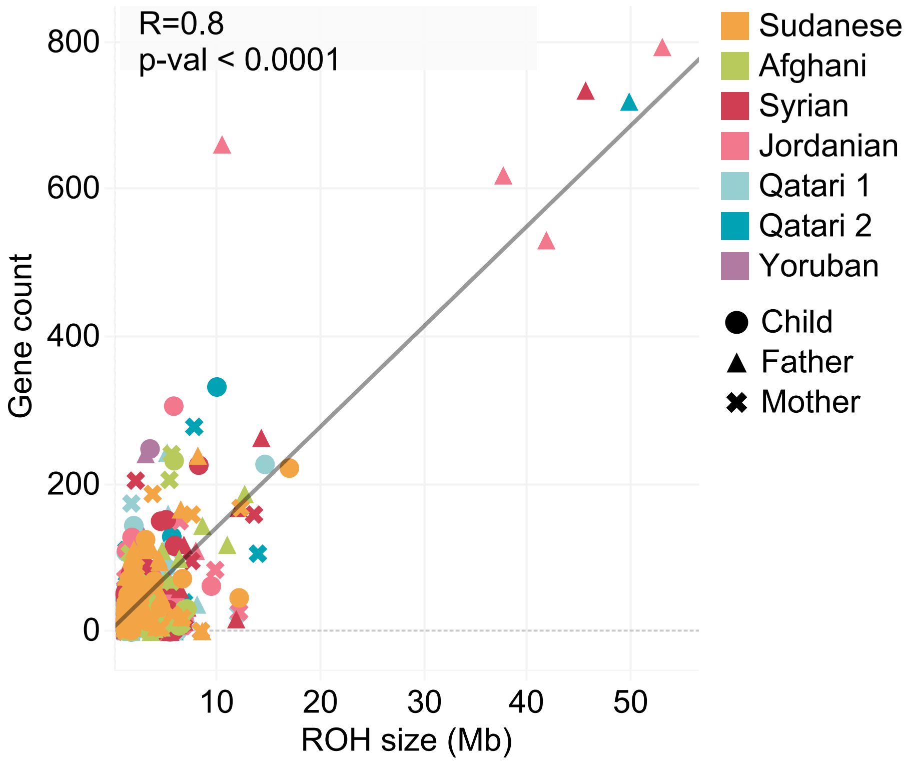

Supplementary Fig. 23: Candidate disease variants short listed from family trio analysis. (a) Count of variants per type and mode of inheritance **(b)** Count of variants per subject and variant type **(c)** Count of variants per subject, variant type and mode of inheritance.

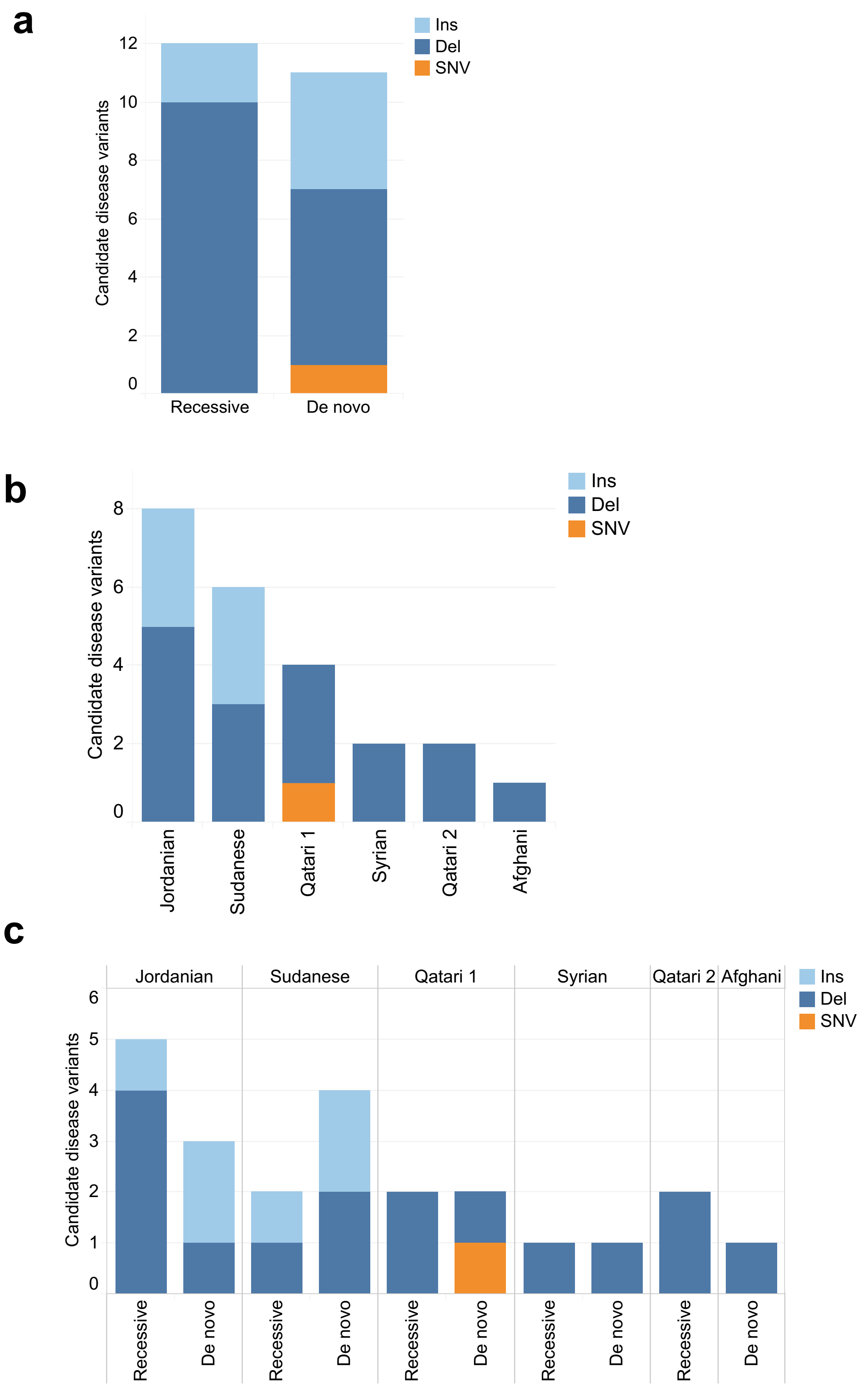

Supplementary Fig. 24: Prioritized variants from classical read-based approach found to be false positive *de novo*. Panels show IGV view of alignments of long reads against either GRCh38 or CHM13, for the mother, father and child for a given trio for *AKT3, TRPM3* and *KMD4B*. For *KMD4B,* alignments are also shown against Jordanian father and HG002. Bar plots indicate coverage. Purple denotes insertion, while other colors denote different bases as per IGV default color scheme.

AKT3

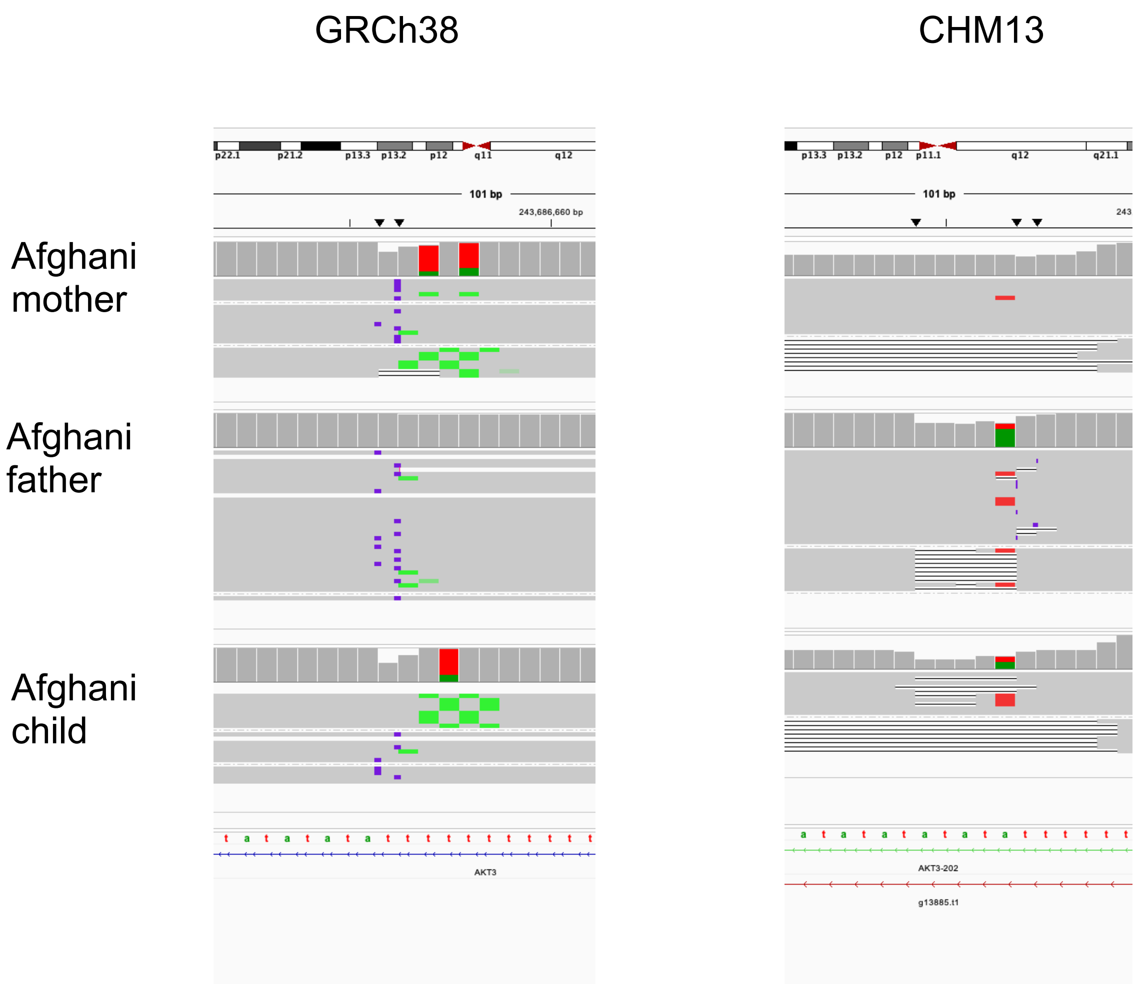

TRPM3

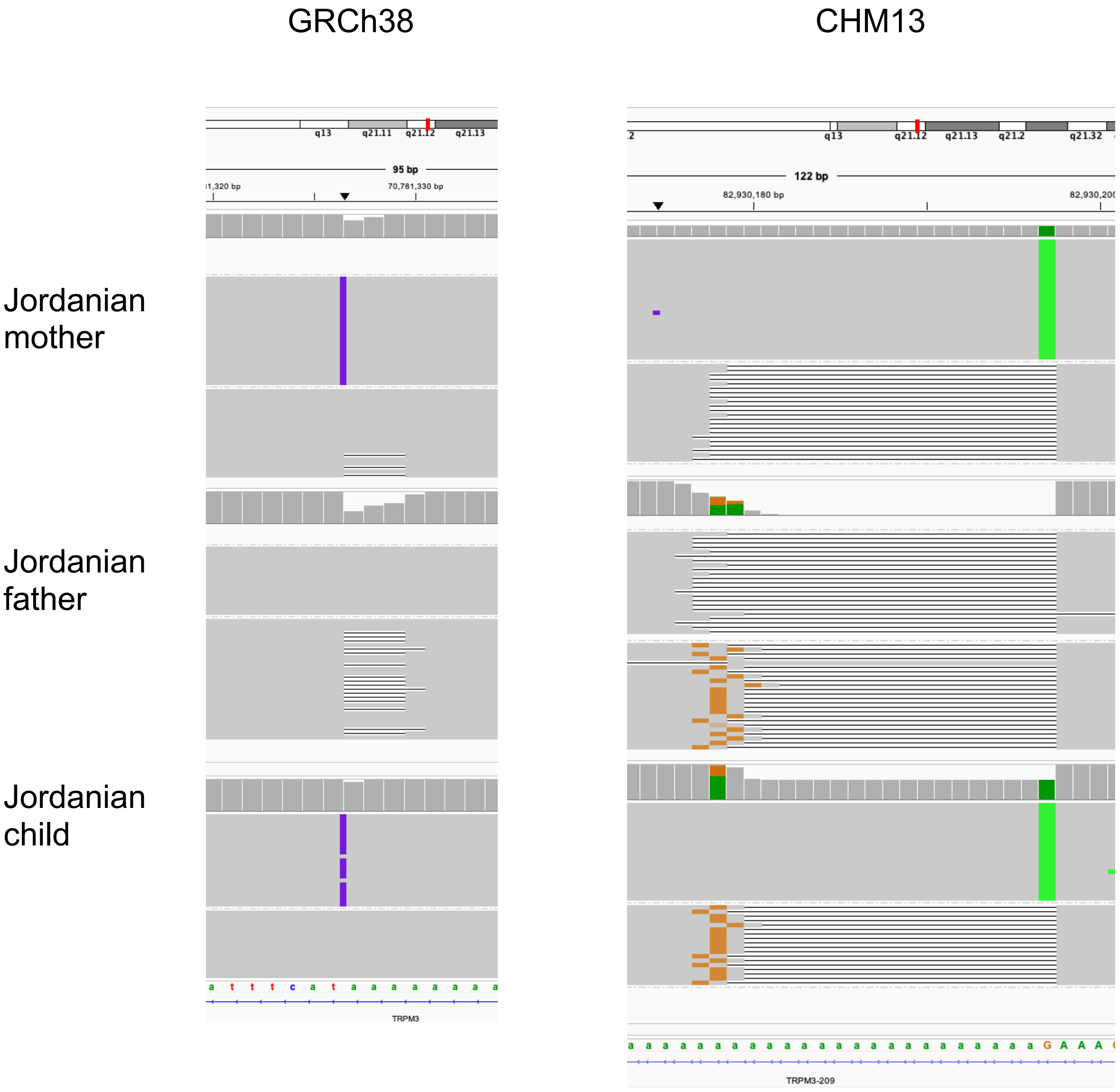
GRCh38
CHM13
Jordanian mother
Jordanian father
Jordanian child
q13
q21.11
q21.12
q21.13
95 bp
70,781,330 bp
a t t t c a t a a a a a a a a
TRPM3
q21.2
q21.32
122 bp
82,930,180 bp
a a a a a a a a a a a a a a a a a a a a a a a a G A A A
TRPM3-209

KMD4B

GRCh38

CHM13

Syrian mother

Syrian father

Syrian child

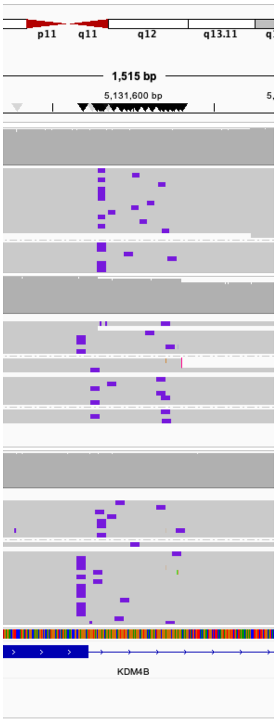

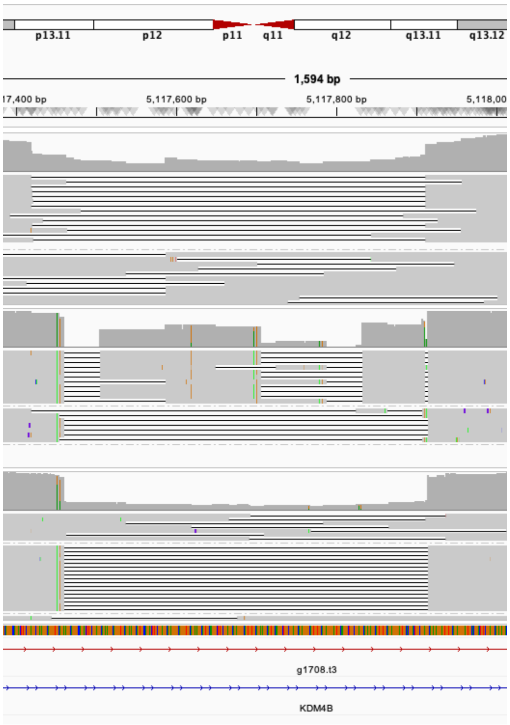

Jordanian father

HG002

Syrian mother

Syrian father

Syrian child

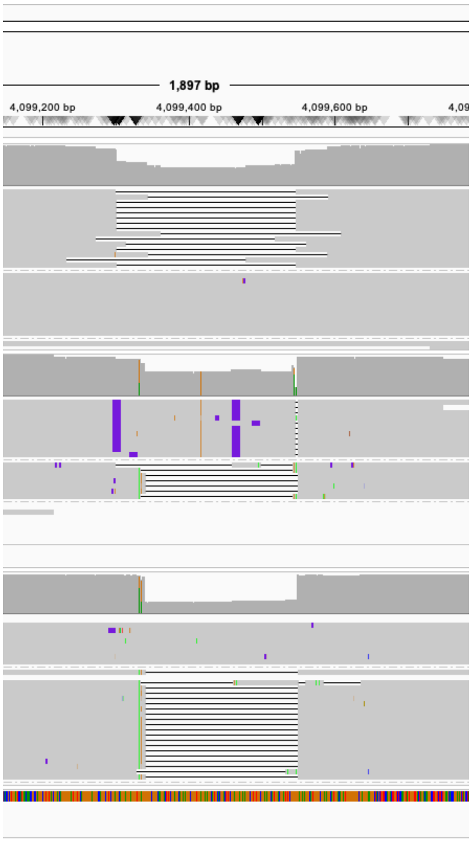

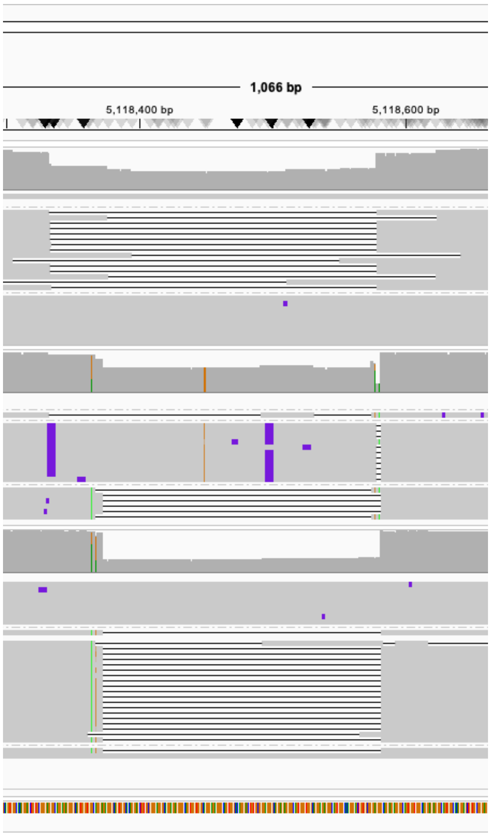

Supplementary Fig. 25: Count of singletons for ME query samples called against MER and other genome references including GRCh38. Difference in count of singletons in the replacement chromosomes relative to MER.

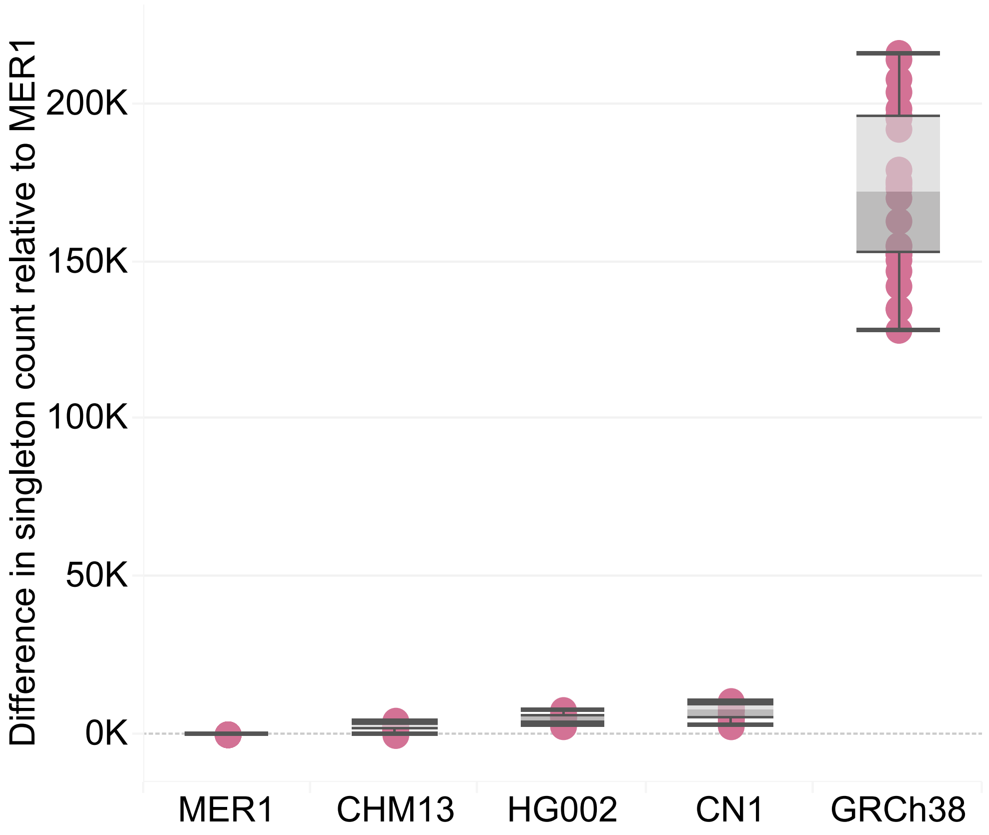

Supplement: Supplementary file 1 — Supplementary Figs. 1–25. [file 41588_2025_2173_MOESM1_ESM.pdf]
