# Supplementary material for: Near-complete Middle Eastern genomes refine autozygosity and enhance disease-causing and population-specific variant discovery
Source: Nat Genet. 2025 May 5;57(5):1119–31. doi: 10.1038/s41588-025-02173-7 (PMC12081309; doi:10.1038/s41588-025-02173-7)
Supplement: Supplementary file 2 — Reporting Summary [file 41588_2025_2173_MOESM2_ESM.pdf]

nature portfolio

Corresponding author(s): Younes Mokrab

Last updated by author(s): Mar 4, 2025

Reporting Summary

Nature Portfolio wishes to improve the reproducibility of the work that we publish. This form provides structure for consistency and transparency in reporting. For further information on Nature Portfolio policies, see our Editorial Policies and the Editorial Policy Checklist.

Statistics

For all statistical analyses, confirm that the following items are present in the figure legend, table legend, main text, or Methods section.

n/a	Confirmed	
☐	☒	The exact sample size (*n*) for each experimental group/condition, given as a discrete number and unit of measurement
☐	☒	A statement on whether measurements were taken from distinct samples or whether the same sample was measured repeatedly
☐	☒	The statistical test(s) used AND whether they are one- or two-sided<br>*Only common tests should be described solely by name; describe more complex techniques in the Methods section.*
☐	☒	A description of all covariates tested
☐	☒	A description of any assumptions or corrections, such as tests of normality and adjustment for multiple comparisons
☐	☒	A full description of the statistical parameters including central tendency (e.g. means) or other basic estimates (e.g. regression coefficient) AND variation (e.g. standard deviation) or associated estimates of uncertainty (e.g. confidence intervals)
☐	☒	For null hypothesis testing, the test statistic (e.g. *F*, *t*, *r*) with confidence intervals, effect sizes, degrees of freedom and *P* value noted<br>*Give P values as exact values whenever suitable.*
☒	☐	For Bayesian analysis, information on the choice of priors and Markov chain Monte Carlo settings
☒	☐	For hierarchical and complex designs, identification of the appropriate level for tests and full reporting of outcomes
☐	☒	Estimates of effect sizes (e.g. Cohen's *d*, Pearson's *r*), indicating how they were calculated

Our web collection on statistics for biologists contains articles on many of the points above.

Software and code

Policy information about availability of computer code

Data collection	Illumina HiSeq X Software v3, SMRT Link v10.1
Data analysis	SAMtools v1.17, mosdepth v0.3.2, FastQC v0.11.9, MultiQC v1.21, SMRTlink v. 10.1, trioHiFiasm 0.16.1, BBmap v38.69, Merqury v1.3, seqtk v1.4, Flagger v0.3.3, Minimap2 v2.9, SafFire v0.2, rustybam v0.1.31, pbmm2 v1.13.0, DeepVariant v1.5.0, PAV v2.3.3, BWA-MEM v 0.7.15 , GATK v4.0.9.0, bcftools v1.17, PLINK v2.00a2LM 64-bit Intel (7 Jan 2019), RFMix v2.0.0, EAGLE v2.4.1, truvari v4.1.0, Liftoff v1.6.3, Immuannot (feb2024), Clustal Omega v1.2.4, TreeViewer v2.2.0 , Automap v1.2, IGV v2.9.4, awk v4.0.2, bedtools v2.3, Jalview 2.11.3, Exomiser v14, DRAGEN 4.2. annotSV v3.4.2 ,Jannovar v0.41 , yak v0.1

For manuscripts utilizing custom algorithms or software that are central to the research but not yet described in published literature, software must be made available to editors and reviewers. We strongly encourage code deposition in a community repository (e.g. GitHub). See the Nature Portfolio guidelines for submitting code & software for further information.

Data

Policy information about availability of data

All manuscripts must include a data availability statement. This statement should provide the following information, where applicable:

- Accession codes, unique identifiers, or web links for publicly available datasets
- A description of any restrictions on data availability
- For clinical datasets or third party data, please ensure that the statement adheres to our policy

The assemblies and sequencing data generated in this study is deposited to dbGAP (Accession ID phs003917.v1.p1). Previously published data used in the study is accessible as follows: 1000 Genomes Project https://hgdownload.cse.ucsc.edu/gbdb/hg19/1000Genomes/phase3, Qatar Biobank/Qatar Genome Project Accession ID: QF-QGP-RES-PUB-007 https://www.qatarbiobank.org.qa/research/how-apply, CHM13 assembly https://s3-us-west-2.amazonaws.com/human-pangenomics/T2T/CHM13/assemblies/analysis_set/chm13v2.0.fa.gz, CHM13 annotation https://ftp.ncbi.nlm.nih.gov/genomes/all/GCF/009/914/755/GCF_009914755.1_T2T-CHM13v2.0/GCF_009914755.1_T2T-CHM13v2.0_genomic.gtf.gz, https://s3-us-west-2.amazonaws.com/human-pangenomics/T2T/CHM13/assemblies/annotation/chm13v2.0_RepeatMasker_4.1.2p1.2022Apr14.bed, https://s3-us-west-2.amazonaws.com/human-pangenomics/T2T/CHM13/assemblies/annotation/chm13v2.0_SD.full.bed, https://s3-us-west-2.amazonaws.com/human-pangenomics/T2T/CHM13/assemblies/annotation/chm13v2.0_censat_v2.1.bed ,GRCh38 assembly https://hgdownload.soe.ucsc.edu/goldenPath/hg38/bigZips/p13/hg38.p13.chromFa.tar.gz, https://s3-us-west-2.amazonaws.com/human-pangenomics/T2T/HG002/assemblies/hg002v1.0.1.fasta.gz, immuannot IPD/KIR dataset https://zenodo.org/records/8372992/files/Data-2023Oct27.tar.gz?download=1 , CIWD v3.0 catalogue https://www.ihiw18.org/component-immunogenetics/download-common-and-well-documented-alleles-3-0/ , gnomad v4.1.0 https://gnomad.broadinstitute.org , CN1 assembly https://genome.zju.edu.cn/files/v1.0.1/CN1_pat.v1.0.1.fasta.gz ,https://genome.zju.edu.cn/files/v1.0.1/CN1_mat.v1.0.1.fasta.gz , https://storage.googleapis.com/gcp-public-data--gnomad/release/4.0/constraint/gnomad.v4.0.constraint_metrics.tsv

Research involving human participants, their data, or biological material

Policy information about studies with human participants or human data. See also policy information about sex, gender (identity/presentation), and sexual orientation and race, ethnicity and racism.

Reporting on sex and gender	Sex of the study subjects was reported as commonly practiced in genomic research
Reporting on race, ethnicity, or other socially relevant groupings	Nationality and genetic ancestry based on admixture analysis and principal component analysis was reported.
Population characteristics	The main study subjects consist of six parent-child family trios (n=18) from the local population in Qatar whereby children were ascertained for neuro developmental disorders and their genetic diagnosis was unresolved. The families are from various nationalities from the greater Middle Eastern region: Sudan, Jordan, Syria, Qatar and Afghanistan. Furthermore, previously published datasets from Qatar Genome Project/Qatar Biobank and 1000 Genomes project and internal Middle Eastern subjects were used. The former consists of Predominantly healthy individuals recruited from the general population at Qatar , aged 18 or over and nationals. 56.7 % of the participants were females whereas 43.3% were males. The latter consists of participants from 26 world populations from the five world continents.
Recruitment	The six families were recruited at Sidra Medicine. Any bias in the selection of the families does not impact the results because each family is analyzed as an independent unit.
Ethics oversight	Informed consent and assent were obtained for all subjects and the study was approved by The Sidra Medicine Ethics Committee.

Note that full information on the approval of the study protocol must also be provided in the manuscript.

Field-specific reporting

Please select the one below that is the best fit for your research. If you are not sure, read the appropriate sections before making your selection.

☒ Life sciences ☐ Behavioural & social sciences ☐ Ecological, evolutionary & environmental sciences

For a reference copy of the document with all sections, see nature.com/documents/nr-reporting-summary-flat.pdf

Life sciences study design

All studies must disclose on these points even when the disclosure is negative.

Sample size	18 subjects were recruited in this study. As comparison reference datasets, we used phase 1 of Qatar Genome Project consisting of 6,216 subjects, 2,504 subjects from 1000 Genomes project phase 3 and 1693 Middle Eastern subjects from internal dataset at Sidra Medicine. No statistical method was used to predetermine the number oft he families because it is a family based study to do genome assembly not a cohort based. Our sample sizes are typical of what is used in the field.
Data exclusions	Standard thresholds for quality measures were used when running various tools for data processing and downstream analyses as mentioned in the manuscript, unless indicated otherwise.

Replication	Experiments were computational so replication is not applicable. For reproducibility describe all of the data sets codes/workflows in the manuscript.
Randomization	Randomization is not applicable to this study because we did not perform any experiments with treatment or control groups that would necessitate randomization between the subjects
Blinding	Blinding is not applicable to this study because we did not perform any experiments with treatment or control groups that would necessitate blinding

Reporting for specific materials, systems and methods

We require information from authors about some types of materials, experimental systems and methods used in many studies. Here, indicate whether each material, system or method listed is relevant to your study. If you are not sure if a list item applies to your research, read the appropriate section before selecting a response.

Materials & experimental systems

n/a	Involved in the study	
☒	☐	Antibodies
☒	☐	Eukaryotic cell lines
☒	☐	Palaeontology and archaeology
☒	☐	Animals and other organisms
☐	☒	Clinical data
☒	☐	Dual use research of concern
☒	☐	Plants

Methods

n/a	Involved in the study	
☒	☐	ChIP-seq
☒	☐	Flow cytometry
☒	☐	MRI-based neuroimaging

Clinical data

Policy information about clinical studies

All manuscripts should comply with the ICMJE guidelines for publication of clinical research and a completed CONSORT checklist must be included with all submissions.

Clinical trial registration	N/A
Study protocol	N/A
Data collection	N/A
Outcomes	N/A

Plants

Seed stocks	*Report on the source of all seed stocks or other plant material used. If applicable, state the seed stock centre and catalogue number. If plant specimens were collected from the field, describe the collection location, date and sampling procedures.*
Novel plant genotypes	*Describe the methods by which all novel plant genotypes were produced. This includes those generated by transgenic approaches, gene editing, chemical/radiation-based mutagenesis and hybridization. For transgenic lines, describe the transformation method, the number of independent lines analyzed and the generation upon which experiments were performed. For gene-edited lines, describe the editor used, the endogenous sequence targeted for editing, the targeting guide RNA sequence (if applicable) and how the editor was applied.*
Authentication	*Describe any authentication procedures for each seed stock used or novel genotype generated. Describe any experiments used to assess the effect of a mutation and, where applicable, how potential secondary effects (e.g. second site T-DNA insertions, mosiacism, off-target gene editing) were examined.*
